# Systematic Analysis Strategy Based on Network Pharmacology to Investigate the Potential Mechanism of *Fritillaria thunbergii* Miq. against Idiopathic Pulmonary Fibrosis

**DOI:** 10.1155/2022/2996878

**Published:** 2022-11-28

**Authors:** Gonghao Xu, Siwen Feng, Rui Sun, Qi Ding, Yuanyuan Shi

**Affiliations:** ^1^School of Life Sciences, Beijing University of Chinese Medicine, Beijing 100029, China; ^2^Shenzhen Research Institute, Beijing University of Chinese Medicine, Shenzhen 518118, China; ^3^School of Chinese Materia Medica, Beijing University of Chinese Medicine, Beijing 100029, China

## Abstract

Idiopathic pulmonary fibrosis (IPF) is a long-term, distressing, and age-related interstitial lung disease characterized by a complicated etiology and irreversible progression. *Fritillaria thunbergii* Miq. (Zhe Beimu, ZBM) is frequently used for its heat-clearing and phlegm-resolving properties in herbal compounds for the treatment of IPF. However, the specific mechanisms underlying the effects of ZBM against IPF have not yet been reported. In this study, we applied a systematic analysis strategy based on network pharmacology to explore the probable core targets and major pathways of ZBM against IPF. In addition, molecular docking simulation and quantitative real-time polymerase chain reaction (qRT-PCR) were performed to preliminarily investigate the possible mechanisms underlying the therapeutic effects of ZBM on IPF. We collected a total of 86 components of ZBM and used network pharmacology analysis to screen nine presumptive targets of ZBM against IPF. The molecular-docking results indicated that the components of ZBM exhibited good binding activity with presumptive targets. The qRT-PCR results also suggested that ZBM may partly alleviate IPF by regulating the expression of presumptive targets. This study laid the foundation for further clinical applications of ZBM and the development of IPF-related therapeutic products.

## 1. Introduction

Idiopathic pulmonary fibrosis (IPF) is a long-term, distressing, and age-related interstitial lung disease characterized by the overactivation of lung fibroblasts and overexpression of extracellular matrix components, which are associated with the remodeling of the lung structure and an irreversible decline in lung function [1–3]. In Europe alone, more than 40,000 people are diagnosed annually with IPF, and the morbidity and mortality caused by IPF have shown an upward trend [4, 5]. With a median survival of only 2-3 years, the quality of survival of IPF patients is discouraging, creating substantial economic and survival pressures for the patients and imposing a huge burden on the society and the medical system [6]. After more than a decade of clinical trials, only two drugs, nintedanib and pirfenidone, have received marketing approval from the Food and Drug Administration for the treatment of IPF. Although these two drugs have shown positive effects in alleviating the decline in lung function in IPF patients, their effects on improving the survival rate or quality of life of patients with IPF are quite limited. Moreover, despite the significance of lung transplantation in improving survival in selected IPF patients, its applicability is greatly limited by the physical condition of IPF patients [7, 8]. Therefore, further development of novel therapeutic strategies for IPF patients is essential.

China, with thousands of years of history in the practice and development of traditional Chinese medicine (TCM), has established its own unique TCM-based treatment system. With the deepening of TCM research, this medical system has gained increasing recognition among clinicians and researchers due to its promising therapeutic effects and relatively mild side effects [9, 10]. The dry bulbs of *Fritillaria thunbergii* Miq. (Zhe Beimu, ZBM), which are mainly produced in Zhejiang, China, have shown wide clinical use in TCM [11, 12]. In clinical practice, ZBM is considered to have the ability to clear away heat, resolve phlegm, relieve cough, remove toxicity, reduce swelling, and dissipate knots, allowing its application for the treatment of lung abscess, lung atrophy, cough, and dyspnea [13, 14]. Modern pharmacological studies have shown that ZBM has antioxidative, anticancer, anti-inflammatory, antitussive, expectorant, antithyroid, and neuroprotective activities [15–17]. In addition, ZBM is frequently used as a heat-clearing and phlegm-resolving agent in TCM for the treatment of IPF [18–20]. However, since previous studies have emphasized the roles of single components and their targeted pathways, the overall mechanism underlying the therapeutic effects of ZBM on IPF requires elucidation [21, 22].

Unlike research on modern drugs, which are identified by targeting specific proteins, TCM research is based on systematic analysis strategies to elucidate the mechanisms of drugs, thereby accounting for the complex composition and extensive coverage of these drugs. With recent improvements in systems biology and biological network equilibrium theory, network pharmacology has gradually emerged as a new model of drug molecule design characterized by “network target and multicausal analysis” [23, 24]. Network pharmacology emphasizes research on the relationships between active ingredients and therapeutic objects from the perspective of the overall connection of biological relationships. Its research philosophy coincides with the holistic theory of TCM [25].

**Figure 1:**
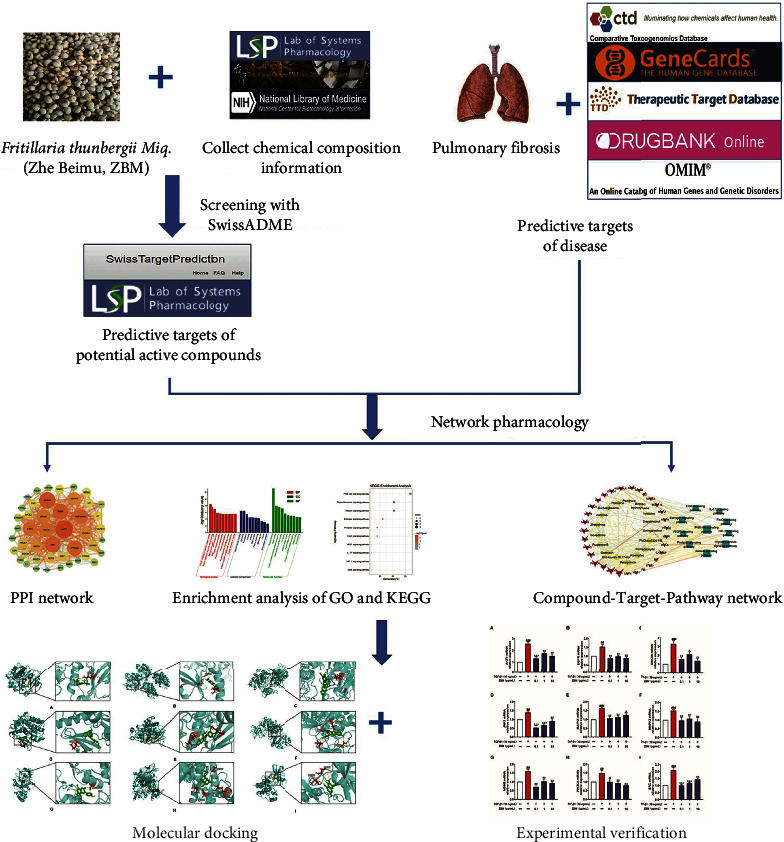
Graphical abstract. This study is based on network pharmacology, combined with molecular docking simulation and experimental verification to investigate the potential mechanism of ZBM against IPF.

In this study, the main compounds of ZBM were collected using the TCM systems pharmacology (TCMSP) database, TCM Database@Taiwan, and the relevant literature. Secondly, compounds with superior pharmacokinetic properties and druggable properties were screened by the SwissADME analysis tool, and network pharmacology analyses were conducted to predict crucial targets and possible mechanisms of ZBM against IPF. In addition, the affinity between components and targets was analyzed by molecular-docking simulations, which verified the reliability of the network pharmacology analysis. Finally, the partial mechanism of ZBM against IPF was verified by experiments. This study provides a new direction for research strategies to elucidate the mechanisms of TCM against IPF from a holistic perspective. The graphic abstract for the study is presented in Figure 1.

## 2. Materials and Methods

### 2.1. Establishment of the Chemical Composition Library of ZBM

“*Fritillariae Thunbrgii* Bulbus” was used as a keyword to obtain chemical information from the TCMSP database, an open system pharmacology database for capturing the relationships among drugs, targets, and diseases [26] (https://old.tcmsp-e.com/tcmsp.php; obtained on April 16, 2022). Similarly, “*Fritillariae Thunbrgii* Bulbus” was used as the search term to collect the relevant chemical composition information in the TCM Database@Taiwan, a noncommercial database for providing downloads of TCM small molecules for virtual screening [27] (https://tcm.cmu.edu.tw/; obtained on April 16, 2022). Additional chemical composition information was obtained from relevant review articles [14, 15]. The chemical composition library of ZBM was established by sorting and classifying the compound information obtained from the databases and literature and eliminating duplicate values and some compounds for which PubChem CIDs were not available. Detailed information regarding the library is provided in the Supporting Information (Supplementary Material S1-Chemical Composition Library of ZBM).

### 2.2. Screening of Compounds with Superior Properties

The SwissADME analysis tool was employed to screen ingredients in the chemical composition library of ZBM. SwissADME can predict the absorption, distribution, metabolism, and excretion (ADME) or pharmacokinetic performance of small molecules on the basis of specific models [28] (https://www.swissadme.ch/; screened on April 26, 2022). Compounds that met all of the following screening conditions were analyzed in the next step: (1) high gastrointestinal (GI) absorption in the pharmacokinetics analysis; (2) 4 or 5 YES conditions in the drug-likeness analysis; and (3) no violations within the YES condition in the drug-likeness analysis. After screening, 17 compounds with superior properties were obtained, and a library of these compounds was established. Detailed information regarding these compounds is presented in the Supporting Information (Supplementary Material S2-Library of Compounds with Superior Properties).

### 2.3. Predictive Targets of Components with Superior Properties in the Compound Library

Information from the simplified molecular input line entry system (SMILES) for components with superior properties in the compound library was obtained from the PubChem website (https://pubchem.ncbi.nlm.nih.gov/; obtained on April 27, 2022). Then, we pasted the SMILES information of the components into the Swiss Target Prediction platform (a platform for predicting possible biological targets of active molecules based on structural similarity algorithms [29] (https://www.swisstargetprediction.ch/; analysis performed on April 27, 2022) to obtain compound targets and retain targets with probability values >0. Compounds for which no targets were provided on the Swiss Target Prediction website were imported into the TCMSP for target collection. After removing duplicate values, the target cluster of compounds with superior properties was constructed. Detailed information is presented in the Supporting Information (Supplementary Material S3-Compounds with Superior Properties and Their Corresponding Targets).

### 2.4. Collection of Potential Targets against IPF

The Comparative Toxicogenomics Database (CTD), GeneCards, Online Mendelian Inheritance in Man Database (OMIM), Therapeutic Target Database (TTD), and DrugBank Database were searched to obtain IPF-related targets. CTD (https://ctdbase.org/) is a publicly available database for studying chemical exposures and their biological effects [30]. GeneCards (https://www.genecards.org) is an integrative database providing functional information on the relationship between human genes and disease [31]. OMIM (https://www.omim.org/) is an online database for describing the association between disease phenotypes and their causative genes [32]. TTD (https://db.idrblab.net/ttd/) is a publicly accessible database for information on targets known to be of therapeutic value for diseases [33]. DrugBank (https://go.drugbank.com/) is a database providing detailed drug data, drug target information, and computerized drug predictions [34]. All information was obtained on April 28, 2022. In each database, “idiopathic pulmonary fibrosis” was used as the key search term. Among the five databases, due to the large amount of target information obtained from the CTD and GeneCards databases, we screened the top 1000 targets according to the inference score values and relevance score values, sorted from high to low. After removing duplicates and collating the results from the five databases, the target cluster of disease was constructed. Finally, the intersection of the target cluster of compounds with superior properties and the target cluster of disease was analyzed by drawing a Venn diagram (https://www.bioinformatics.com.cn; obtained on April 29, 2022) to represent potential targets of ZBM against IPF. Specific information regarding the targets is provided in the Supporting Information (Supplementary Material S4-Details of Intersection Targets).

### 2.5. Analysis and Construction of the Protein-Protein Interaction Network

The Search Tool for the Retrieval of Interacting Genes/Proteins database (STRING, a database for exploring and analyzing functional connections between proteins based on associations between genomes [35], https://cn.string-db.org/; obtained on April 30, 2022) was applied to analyze the intersection target cluster. The data analyzed by the STRING database was exported in the TSV format and imported into Cytoscape software (v3.7.2, Massachusetts, USA) for network analysis. The medians of the three parameters, “betweenness centrality,” “closeness centrality,” and “degree,” were calculated by the software. The crucial gene cluster was screened by retaining genes whose corresponding data values were greater than medians. Network analysis and visualization of the crucial gene clusters were performed in Cytoscape software. Detailed information regarding the crucial gene clusters is presented in the Supporting Information (Supplementary Material S5-Information Regarding the Crucial Gene Cluster).

### 2.6. Enrichment Analysis through Gene Ontology and Kyoto Encyclopedia of Genes and Genome

The list of crucial genes obtained in Section 2.5 was entered into the Database for Annotation, Visualization, and Integrated Discovery (DAVID, version 6.8, a database for providing gene function annotation and related biological information [36], https://david.ncifcrf.gov/tools.jsp; obtained on April 30, 2022) to analyze the biological links among them. Gene ontology (GO) and Kyoto Encyclopedia of Genes and Genomes (KEGG) enrichment analysis were used to obtain information regarding the biological function and signaling pathways of the crucial gene clusters. Then, the microbiographic mapping platform (https://www.bioinformatics.com.cn/; employed on May 2, 2022) and Cytoscape software were employed to visualize the results by drawing a dot bubble chart, GO term chart, and the “compound-target-pathway” network. The correspondence information between active compounds, core targets, and pathways was input into Cytoscape, which performed network analysis among these elements and sorted them according to the “degree” value.

### 2.7. Molecular Docking Simulation

The 3D structure information of the proteins was obtained from the RCSB Protein Data Bank (PDB) (https://www.rcsb.org/, obtained on May 10, 2022, PDB IDs are shown in Table 1). After removing water molecules, heteroatoms, and original ligands in the protein structures with PyMOL software (v2.4.0, New York, USA, https://www.pymol.org/), hydrogen bonds were added to protein molecules in MGLTools software (v1.5.6, California, USA, https://ccsb.scripps.edu/mgltools/). The proteins were then processed by calculating their Gasteiger charge values and assigning AD4 atom types and saved in the PDBQT format as receptor molecules. By searching the positions of the existing ligands in protein receptors, the docking boxes of the functional pockets of protein receptors were determined to prepare for molecular docking. Subsequently, compound molecules in the SDF 3D format downloaded from PubChem were converted to the PDB 3D format, and the 3D compound molecules were imported into MGLTools software for the detection of roots and selection of torsions. Then, the processed compound molecules were saved in the PDBQT format as ligand molecules and treated together with the processed receptor protein molecules to perform molecular docking simulation in AutoDock Vina software (v1.1.2, California, USA, https://vina.scripps.edu/, a program for molecular docking and virtual screening [37]) to obtain docking affinity scores and docking information. Finally, the docking results were visualized with PyMOL software. Where the binding energies were equal, the conformation that contained hydrogen bonds or contained more hydrogen bonds was preferentially displayed.

### 2.8. Drug Preparation

The TCM *Fritillaria thunbergii* Miq. material was purchased from the Chinese herbal medicine market (Anhui, China) and authenticated by Dr. Ding Qi (Shenzhen Research Institute, Beijing University of Chinese Medicine, Beijing, China). A total of 10.03 g of the TCM material was soaked for 2 h with ten times the amount of double distilled water and then refluxed for 1 h. After filtering the extracted liquid, the drug residues were extracted with eight times the amount of water under reflux for 1 h. The two filtrated liquids were combined, concentrated, and dried to finally obtain 1.68 g of ZBM extract powder (a yield of 16.75%). The extract powder was dissolved in dimethyl sulfoxide (DMSO) before the experiment.

### 2.9. Cell Culture and Treatment

Human embryonic lung fibroblast cell line MRC-5 (BNCC353614) was purchased from BeNa Culture Collection (Henan, China). A basal culture of MRC5 cells was established using Dulbecco's Modified Eagle Medium (DMEM) containing 12% fetal bovine serum, and the cells showed good proliferation at 37°C and 5% CO_2_. When the cells were ready for plate laying, they were digested with 0.25% trypsin and inoculated at a density of 3 × 10^4^ cells/well in 96-well plates or a density of 4 × 10^5^ cells/well in 6-well plates. After starvation for at least 4 h, the cells in the administration groups were treated with transforming growth factor-*β*1 (TGF-*β*1) stimulators (10 ng/mL; Proteintech, Wuhan, China) for an additional 48 h in the absence or presence of ZBM extracts (0.01–10 *μ*g/mL).

### 2.10. Cell Viability Assay with the Cell Counting Kit-8

The cell counting kit-8 (CCK-8, MedChemExpress, Shanghai, China) was used to detect the proliferation of MRC-5 cells after treatment with ZBM extracts and TGF-*β*1. Briefly, the day after cell inoculation into 96-well plates, cells were starved with serum-free medium for at least 4 h. The cells were then treated with ZBM (0, 0.01, 0.1, 1, and 10 *μ*g/mL) and TGF-*β*1 for an additional 48 h. After adding 10% CCK-8 reagent to the cell-containing wells, the plates were allowed to react for an additional 1-2 h at 37°C under light-proof conditions. Finally, the optical density at 450 nm was detected using a multifunctional microplate reader.

### 2.11. Quantitative Real-Time Polymerase Chain Reaction Analysis

Quantitative real-time polymerase chain reaction (qRT-PCR) is a useful technique for quantitative analysis of gene expression [38]. RNA extraction kits (Tian Gen, Beijing, China) were used to extract total RNA. After determination of the RNA concentration with a NanoDrop One Microvolume Spectrophotometer (Thermo Fisher Scientific, Massachusetts, USA), and HiScript II Q RT SuperMix reagent (Vazyme, Nanjing, China) was added to all RNA samples (quantified to 1000 ng) for reverse transcription. The reverse-transcribed samples were cyclically amplified with ChamQ SYBR qPCR Master Mix (Vazyme, Nanjing, China) in a Roche LightCycler 480 (Roche, Basel, Switzerland) with GAPDH as the internal reference gene. The PCR conditions were as follows: 95°C for 30 s followed by 40 cycles at 95°C for 10 s and 60°C for 30 s. Data were processed using the 2^−ΔΔct^ calculation method. Primers were purchased from Sangon Biotech (Shanghai, China); detailed information regarding the primers is presented in Table 2.

### 2.12. Statistical Analysis

All experimental data were obtained from at least three independently performed experiments. GraphPad Prism 8.0 (GraphPad Software, California, USA, https://www.graphpad.com/) was used for statistical analysis of the data. The paired sample *T*-test was applied for comparison between the two groups. A *P* value <0.05 indicated that the difference was statistically significant.

## 3. Results

### 3.1. Chemical Composition Library of ZBM

We collected compound information from the TCM Database@Taiwan, TCMSP databases, and literature. After removing duplicates and compounds without structure information, the remaining 86 compounds were used to establish the chemical composition library of ZBM. The main components of ZBM included alkaloids, diterpenoids, and essential oils, among others. Detailed information regarding the library is presented in the Supporting Information (Supplementary Material S1, Chemical Composition Library of ZBM).

### 3.2. Screening of Compounds with Superior Properties and Target Prediction

Using the approach described in Section 2.2, a total of seventeen compounds with superior properties were screened from the 86 compounds in the chemical composition library of ZBM. Detailed information is presented in the Supporting Information (Supplementary Material S2-Library of Compounds with Superior Properties). Next, 410 compound targets were obtained to construct a target cluster of compounds with superior properties through the SwissTargetPredicition platform after removing duplicate values. Information about these compounds with superior properties and their corresponding targets is presented in the Supporting Information (Supplementary Material S3-Compounds with Superior Properties and Their Corresponding Targets).

### 3.3. Collection of IPF Disease Targets

**Figure 2:**
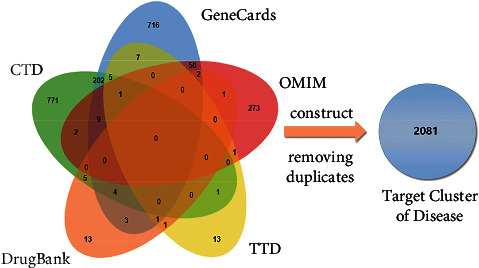
Venn diagrams of targets related to IPF obtained from five databases. Targets related to IPF were from the CTD (green), genecards (blue), OMIM (red), TTD (yellow), and DrugBank (brown) databases. The numbers represented the number of targets that were unique or common to each database. The target clusters of diseases were constructed after removing duplicates.

We collected information on targets related to IPF from the CTD (1000 targets), GeneCards (1000 targets), OMIM (339 targets), TTD (30 targets), and DrugBank (30 targets) databases. A total of 2081 IPF-related targets were obtained to construct the target cluster of disease after removing duplicates. The results were visualized with Venn diagrams (Figure 2).

### 3.4. Construction of the Protein-Protein Interaction Network and Selection of the Crucial Gene Cluster

**Figure 3:**
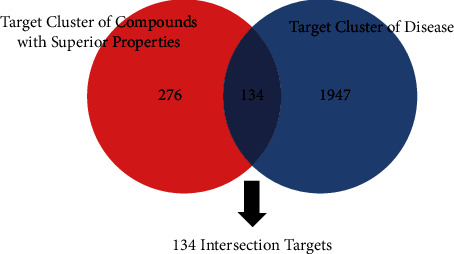
Venn diagram of the target cluster. The red part represented the target cluster of compounds with superior properties. The blue part represented the target cluster of disease. The crossed part represented the potential targets of ZBM against IPF.

**Figure 4:**
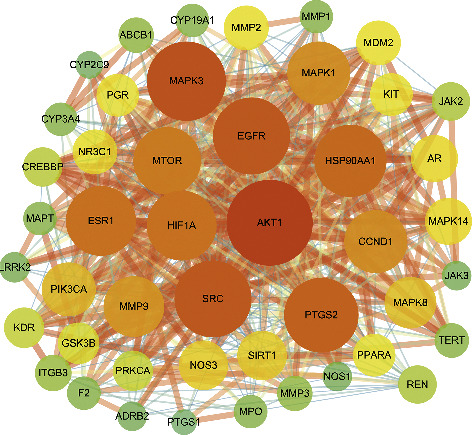
The protein-protein interaction network of the crucial gene clusters. As the degree of the target decreased, the circle became smaller and the color became lighter (from dark red to light green). As the combined score decreased, the combined line became thinner.

A total of 134 cross-targets were identified from the intersection of the target cluster of compounds with superior properties and the target cluster of disease (Figure 3). The STRING database and Cytoscape software were used to construct the protein-protein interaction network. After calculating the median values of the parameters “betweenness centrality” (median value = 0.00192664), “closeness centrality” (median value = 0.47482014), and “degree” (median value = 14), 46 core targets were selected to construct the crucial gene cluster. The network relationships in the crucial gene clusters were shown by Cytoscape software (Figure 4). Specific information is presented in the Supporting Information (Supplementary Material S4-Details of Intersection Targets) and the Supporting Information (Supplementary Material S5-Information regarding the Crucial Gene Cluster).

### 3.5. GO and KEGG Pathway Enrichment Analysis

**Figure 5:**
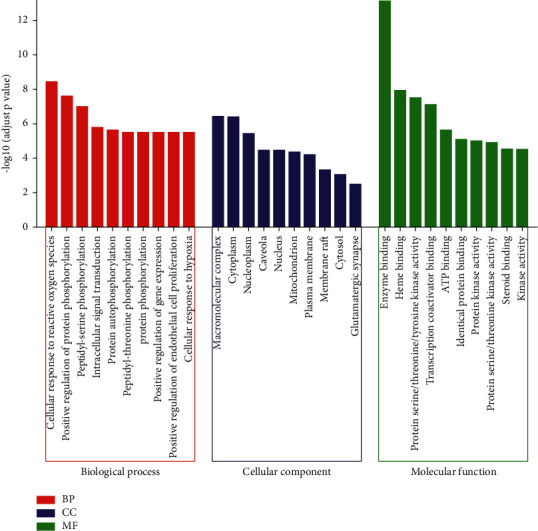
Merged bar graph based on GO enrichment analysis. The abscissa represented the top 10 items in the BP (red), CC (blue), and MF (green) analysis results. The ordinate represented the −log10 value of the adjusted *P* value.

**Figure 6:**
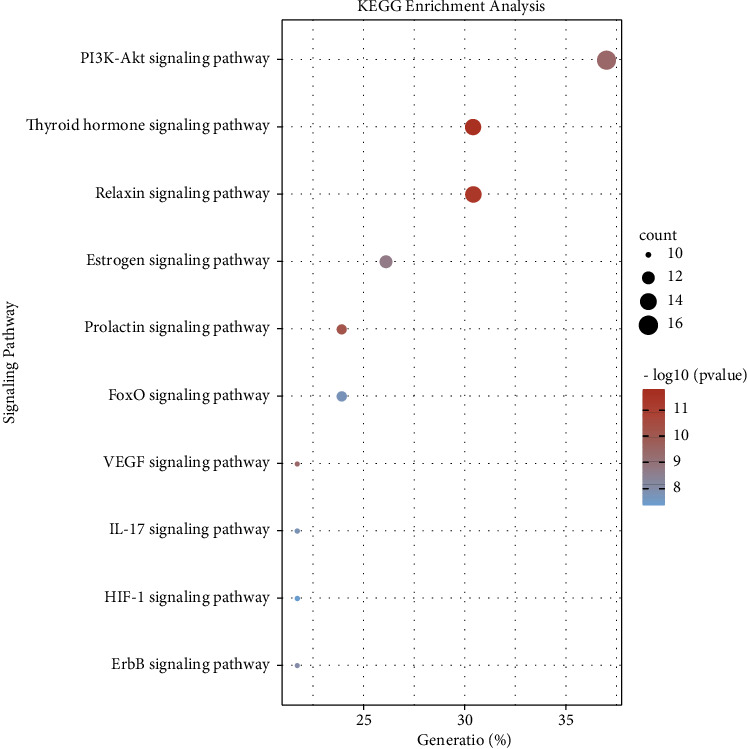
Dot bubble chart of KEGG enrichment analysis. The abscissa represented the gene ratio of the top 10 signaling pathways. The size of the bubbles represented the number of genes. Colors from blue to red represented the −log_10_ value of the adjusted *P* value from low to high. The arrangement order of signaling pathways was determined based on the count value from high to low.

The list of 46 core targets was pasted into the DAVID database for KEGG and GO enrichment analysis to further investigate the role of core targets in the development of IPF. The top 10 items in the biological process (BP), cellular component (CC), and molecular function (MF) analysis results in the GO enrichment analysis were presented in the form of a merged bar graph in Figure 5. The results indicated that core targets affect the development of IPF mainly by regulating biological processes such as cellular response to reactive oxygen species, regulation of protein phosphorylation, cell proliferation, and the cellular response to hypoxia. In addition, using KEGG enrichment analysis, the top 10 signaling pathways involved in IPF disease related to core targets were selected and visualized with a dot bubble chart (Figure 6). On the basis of the existing research on IPF, the enriched KEGG pathways were divided into hormone regulation-related signaling pathways (thyroid hormone, relaxin, prolactin, and estrogen), cell growth state regulation-related signaling pathways (VEGF, PI3K/AKT, ErbB, FoxO, and HIF-1), and immune regulation-related signaling pathways (IL-17).

### 3.6. Compound-Target-Pathway Network Construction

**Figure 7:**
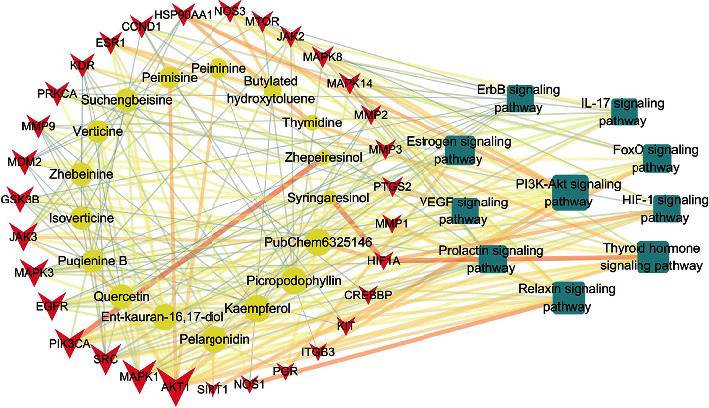
Compound-target-pathway network. Targets were represented in the form of pink arrows. Signaling pathways were represented in the form of blue round rectangles. Compounds were represented in the form of yellow ellipses. The sizes of the shapes varied according to the degree value.

Based on the top 10 signaling pathways obtained above, the compound-target-pathway network, including 17 compounds with superior properties and 31 core targets, was constructed by Cytoscape software to analyze the relationships among them. As shown in Figure 7, the compound-target-pathway network consisted of 58 nodes and 229 edges. Through network analysis, potential active compounds, core targets, and signaling pathways were sorted according to their degree values. The terms with higher degree values were considered more promising objects. AKT1, MAPK1, SRC, PIK3CA, MAPK3, EGFR, JAK3, GSK3B, and MDM were the top nine targets. Pelargonidin, kaempferol, picropodophyllin, [(4R, 9R, 10S,1 4R, 16R)-5, 5, 9-trimethyl-15-oxapentacyclo [11.3.1.01, 10.04, 9.014,16] heptadecan-14-yl] methanol (PubChem6325146), Ent-kauran-16, and 17-diol were the top five compounds.

### 3.7. Molecular Docking

**Figure 8:**
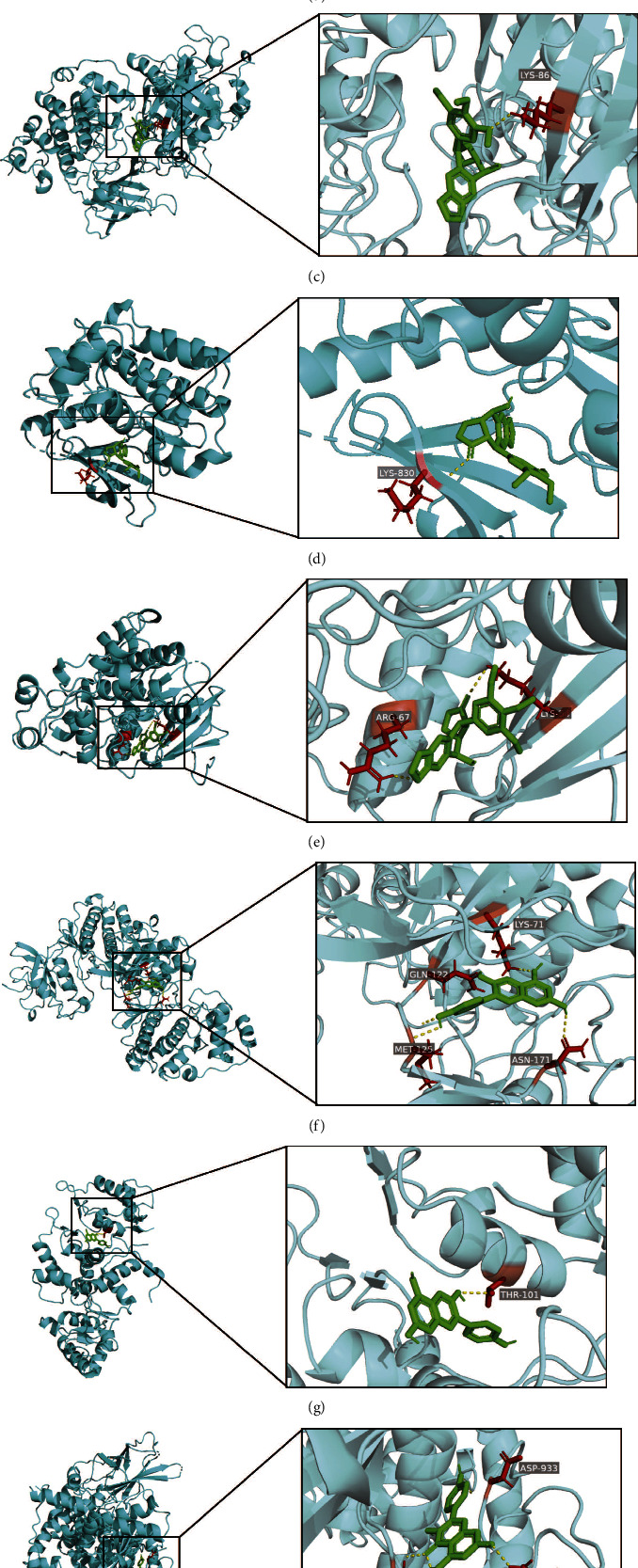
Representative illustration of the molecular docking simulation of ligands and receptors. (a) PubChem6325146 docked with AKT1. (b) Pelargonidin docked with EGFR. (c) Picropodophyllin docked with GSK3B. (d) Picropodophyllin docked with JAK3. (e) Picropodophyllin docked with MAPK1. (f) Pelargonidin docked with MAPK3. (g) Pelargonidin docked with MAPK3. (h) Kaempferol docked with PIK3CA. (i) Kaempferol docked with SRC. The protein receptors were presented in cyan. Small molecule ligands were presented in green. The binding part of the receptor and ligand was magnified, and the hydrogen bonds were presented in yellow dotted lines. Amino acid residues of the protein receptor that interacted with the ligand through hydrogen bonds were presented in red.

We simulated the binding of the compounds and the targets through molecular-docking analyses to further analyze the connection between the targets and compounds. After treatment, five compounds were used as small molecule ligands and nine target proteins were used as docking receptors. Using AutoDock Vina software, semiflexible simulated docking was performed between them. The docking results were presented as affinity scores obtained with AutoDock Vina software (Table 3). The larger the absolute value of the docking affinity score, the stronger the binding ability. The results showed that the docking affinity scores of all compounds had scores greater than −5.0 kcal/mol, and most of them had scores greater than −7.0 kcal/mol, indicating good binding among the top nine targets and five compounds. The groups with the highest affinity score with target proteins and compounds were visualized with PyMOL software and are presented in Figure 8. The abovementioned results suggested that the constituents in ZBM may show favorable interactions with the top nine targets and exert antipulmonary fibrosis effects through these targets. However, further experiments are required to elucidate and validate these findings.

### 3.8. Effects of ZBM on the Viability of MRC-5 Cells

**Figure 9:**
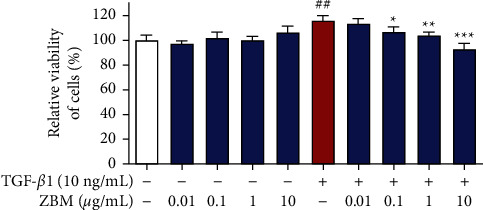
Effects of ZBM on the viability of MRC-5 cells with or without TGF-*β*1. MRC-5 cells were incubated with DMSO (solvents for ZBM extract) for 48 h as the control group. MRC5 cells were incubated with ZBM (0.01, 0.1, 1, and 10 *μ*g/mL) and TGF-*β*1 (10 ng/mL) for 48 h as the administration group. The cell viability was detected using the CCK-8 assay. Data were presented as the mean ± standard deviation (*n* = 4). ^##^*p*  <  0.01 versus the control group; ^*∗∗∗*^*p*  <  0.001, ^*∗∗*^*p*  <  0.01, and ^*∗*^*p*  <  0.05 versus the model group.

We conducted in vitro experiments to further verify the correctness of the network pharmacology method and the results of molecular docking. First, we investigated the effect of the ZBM extract on the viability of MRC-5 cells. As shown in Figure 9, in the absence of TGF-*β*1 stimulators, ZBM at a concentration of 0.01–10 *μ*g/mL showed no significant effect on the cell viability of MRC-5 cells, suggesting that ZBM is safe and reliable within a concentration range of 0.01–10 *μ*g/mL. In comparison with the control group (no stimulation or drug), the cell viability in the model group (only TGF-*β*1 treatment) increased significantly after administration of the TGF-*β*1 stimulator (10 ng/mL). However, in the group that received simultaneous administration of TGF-*β*1 and drugs, ZBM at concentrations of 0.1–10 *μ*g/mL significantly inhibited the cell proliferation effect induced by TGF-*β*1.

### 3.9. Effects of ZBM on mRNA Expression of Collagen and *α*-SMA in MRC-5 Cells

**Figure 10:**
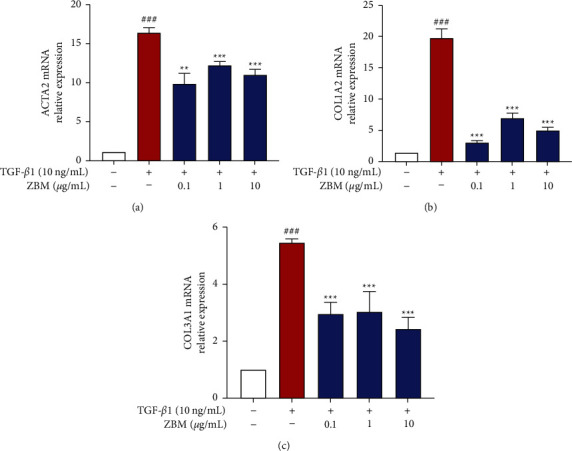
Effects of ZBM on mRNA expression of collagen and *α*-SMA in MRC-5 cells. MRC-5 cells were incubated with DMSO for 48 h as a control group. MRC-5 cells were incubated with ZBM (0.1, 1, and 10 *μ*g/mL) and TGF-*β*1 (10 ng/mL) for 48 h as the administration group. The mRNA expression of ACTA2 (a), COL1A2 (b), and COL3A1 (c) were detected using a qRT-PCR assay. Data were presented as the mean ± standard deviation (*n* = 3). ^###^*p*  <  0.001 versus the control group; ^*∗∗∗*^*p*  <  0.001 and ^*∗∗*^*p*  <  0.01 versus the model group.

Lung myofibroblasts are key effector cells of pulmonary fibrosis [39]. Overexpression of collagen and *α*-smooth muscle actin (*α*-SMA) is one of the characteristics of lung myofibroblasts in comparison with normal lung fibroblasts [40]. Therefore, we next investigated the effect of ZBM administration on *α*-SMA and collagen expression in MRC-5 cells to determine the effect of ZBM on the process of fibroblast transdifferentiation into myofibroblasts. As shown in Figure 10, the mRNA expression levels of ACTA2, COL1A2, and COL3A1 were significantly increased in the TGF-*β*1-stimulated group, while administration of 0.1–10 *μ*g/mL ZBM resulted in significant improvement, indicating that ZBM can inhibit the transformation of fibroblasts into myofibroblasts.

### 3.10. Exploration of the Possible Mechanisms Underlying the Therapeutic Effects of ZBM on IPF

**Figure 11:**
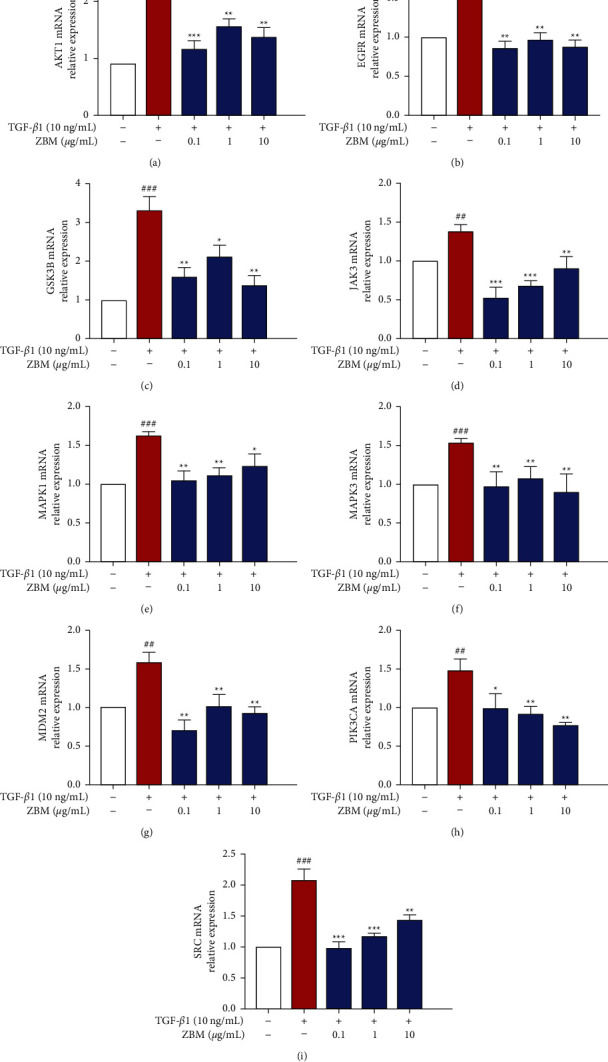
Effects of ZBM on mRNA expression of core targets in MRC-5 cells. The MRC-5 cells were incubated with DMSO for 48 h as the control group. MRC-5 cells were incubated with ZBM (0.1, 1, and 10 *μ*g/mL) and TGF-*β*1 (10 ng/mL) for 48 h as the administration group. The mRNA expression of AKT1 (a), EGFR (b), GSK3B (c), JAK3 (d), MAPK1 (e), MAPK3 (f), MDM2 (g), PIK3CA (h), and SRC (i) was detected using the qRT-PCR assay. Data were presented as the mean ± standard deviation (*n* = 3). ^###^*p*  <  0.001 and ^##^*p*  <  0.01 versus the control group; ^*∗∗∗*^*p*  <  0.001, ^*∗∗*^*p*  <  0.01, and ^*∗*^*p*  <  0.05 versus the model group.

Combined with the results of the above network pharmacology, we performed additional experimental analysis of the core targets to investigate the possible mechanisms underlying the therapeutic effects of ZBM on IPF. As shown in Figure 11, the mRNA expression levels of AKT1, EGFR, GSK3B, JAK3, MAPK1, MAPK3, MDM2, PIK3CA, and SRC were increased in the TGF-*β*1-stimulated group, while administration of 0.1–10 *μ*g/mL ZBM resulted in various degrees of improvement in these expression levels, indicating that ZBM inhibited IPF partially by regulating the expression levels of these targets and that the systematic analysis strategy based on network pharmacology was reliable.

## 4. Discussion

Although many risk factors for IPF, including smoking, drug exposure, pollutant exposure, viral infection, and genetic susceptibility, have been identified after decades of clinical research, therapeutic options based on the complex pathogenesis of this disease have still not been developed [41]. Multiple biological regulatory processes involving complex signaling pathways, including cellular metabolism, regulation of cell survival state, cellular senescence, immune stress, and hormone regulation, have been reported to contribute to the development of fibrosis [42, 43]. Given the suboptimal clinical outcomes of most existing single-targeted or single-pathway small molecule drugs such as SAR156597, simtuzumab, and lebrikizumab, the development of drugs with multitargeted effects may be a better approach for the treatment of IPF [8]. Because of the unique features of TCM, the principles and drugs used in this system of medicine are being increasingly employed in the treatment of and research on IPF worldwide, potentially serving as an attractive source for the identification of anti-IPF drugs through a multilevel, multitarget, and multipathway approach [10]. ZBM is frequently used in herbal compounds for the treatment of IPF, but its mechanism of action is still unclear and merits an in-depth investigation.

The existing understanding of the pathogenesis of IPF has been enhanced by ongoing research. In general, under the stimulation of multiple risk factors, repeatedly injured alveolar epithelial cells undergo abnormal activation or apoptosis, resulting in the release of multiple cytokines to promote abnormal recruitment and activation of lung fibroblasts [7, 44, 45]. These activated lung fibroblasts secrete large amounts of extracellular matrix components, which is a key aspect of the pulmonary fibrosis process [46]. In our study, we stimulated the activation of lung fibroblasts in vitro with TGF-*β*1. We found that ZBM significantly reduced the mRNA expression levels of ACTA2, COL1A2, and COL3A1 in the TGF-*β*1-stimulated lung fibroblasts. This result suggested that ZBM had an antipulmonary fibrosis effect in vitro.

For complex diseases, most drug studies focused on a single drug or a single target end in failure. Consequently, with the development of the field of network pharmacology, an increasing number of studies are employing synergistic, multicompound approaches for therapeutic drug development, which can favorably accelerate clinical translation [47]. In this study, by employing an analytical method based on network pharmacology, we predicted nine targets showing the highest association with the therapeutic effects of ZBM against IPF. Since molecular docking is the most used tool in structure-based drug design and optimization studies [48], we performed molecular-docking simulations using AutoDock Vina software for evaluating the binding of the top nine targets and five compounds. The results revealed strong binding abilities of each compound with each target, implying that ZBM may exert antipulmonary fibrosis effects through these targets. To gain a comprehensive understanding of the roles of these nine crucial targets in IPF and the interactions among them, we conducted a literature survey and performed in vitro validation experiments with qRT-PCR.

The phosphatidylinositol 3-kinase/protein kinase B (PI3K/AKT) signaling pathway is considered to be a major regulator of IPF, through its involvement in cell growth, differentiation, and metabolism [49]. Activation of the PI3K/AKT is closely related to the overexpression of *α*-SMA in pulmonary fibrosis and interactions with the TGF-*β* signaling pathway, promoting the development of pulmonary fibrosis [50]. Specifically, the expression of phosphorylated AKT was shown to be significant in live-cultured, precisely dissected lung sections obtained from IPF patients, while the progression of fibrosis was suppressed in a concentration-dependent manner after administration of GSK2126458 (a potent and highly selective inhibitor of PI3K) [51]. In our study, the mRNA expression levels of PIK3CA and AKT1 in MRC-5 cells increased after TGF-*β*1 stimulation and reduced after administration of ZBM, suggesting that ZBM has a regulatory effect on the PI3K/AKT pathway. Mouse double minute 2 (MDM2) mediates the ubiquitination of p53 (one of the key regulators of IPF [52]) and is responsible for the inhibition of p53 activity, making it important for the regulation of cellular homeostasis [53]. Interestingly, AKT can promote the activation of MDM2 through phosphorylation, indicating that the regulation of AKT signaling also affects MDM2 [54]. Furthermore, GSK3B is a serine/threonine kinase that can be activated by the AKT signaling cascade to regulate TGF*β* and *β*-catenin signaling pathways to influence the development of pulmonary fibrosis [55–57]. In our study, we observed the same trend for MDM2 and GSK3B in TGF-*β*1-stimulated MRC-5 cells, whereas the administration of ZBM had an inhibitory effect. Taken together, these findings suggest that ZBM may regulate the development of pulmonary fibrosis partly through the PI3K/AKT/MDM2 and PI3K/AKT/GSK3B signaling pathways.

The mitogen-activated protein kinase (MAPK) signaling cascade, which is responsible for the transmission of extracellular signals to intracellular targets, is critical for cell growth, survival, and stress [58]. The MAPK signaling cascade has been reported to mediate the regulation of the TGF-*β*1 signaling pathway, which is critical for the fibrotic process [59]. TGF-*β*-induced epithelial-mesenchymal transition (EMT) is dependent on the activation of the MAPK signaling cascade [60]. Mefunidone was shown to reduce the expression of Snail and vimentin by inhibiting the transduction of the TGF-*β*/Smad2 signaling pathway and the activation of the MAPK pathway, thereby inhibiting the process of EMT and improving pulmonary fibrosis [61]. In our study, MAPK1 (ERK2) and MAPK3 (ERK1) were enriched. We studied the changes in MAPK1 and MAPK3 expression after administration of TGF*β*1 and ZBM and found that ZBM could significantly reduce the TGF-*β*1-induced elevation of MAPK1 and MAPK3 expression in MRC-5 cells. Therefore, we speculate that ZBM may partially prevent the development of pulmonary fibrosis by inhibiting the MAPK signaling pathway.

Janus kinases (JAKs) are a group of intracellular tyrosine kinases that form the JAK/STAT signaling cascade with downstream signal transducers and activators of transcription (STAT), which play a regulatory role in the development of fibrosis [62]. The JAK/STAT pathway is activated through the binding of multiple cytokines to their specific plasma membrane receptors, thereby regulating the cellular transformation of fibroblasts to myofibroblasts, EMT, immune regulation, and cell survival [63, 64]. Fei Kang, a TCM compound prescription with beneficial clinical efficacy against IPF, has been reported to play an antipulmonary fibrosis effect by inhibiting the expression levels of proteins related to the JAK1/STAT3 signaling pathway [65]. In our study, ZBM significantly reduced the expression level of JAK3 after TGF-*β*1 stimulation in MRC-5 cells, indicating that ZBM regulated the JAK signaling pathway. On the basis of these findings, we speculate that ZBM can also exert antipulmonary fibrosis effects through the JAK/STAT signaling axis. Interestingly, the epidermal growth factor receptor (EGFR), a member of the ErbB tyrosine kinase receptor family, was shown to participate in wound healing and tissue regeneration by activating downstream PI3K/AKT, MAPK/ERK, and STAT signaling pathways [66]. In our study, we found that ZBM also significantly modulated the expression of EGFR and SRC, a nonreceptor tyrosine kinase that activates STAT3 and trans-activates EGFR. Therefore, we speculated that EGFR and SRC act as regulators of the abovementioned multiple pathways.

**Figure 12:**
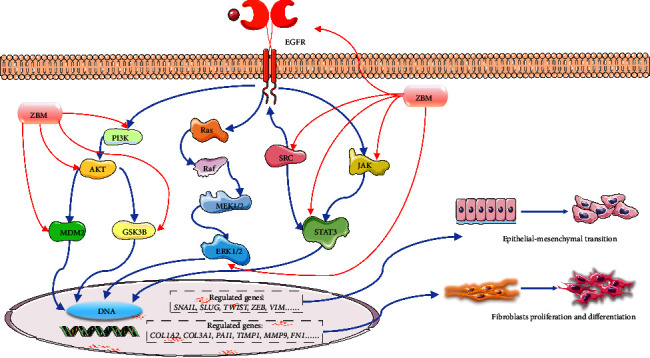
Hypothetical illustration of the regulation of IPF-related pathways by ZBM. Nine targets, AKT1, MAPK1, SRC, PIK3CA, MAPK3, EGFR, JAK3, GSK3B, and MDM2, play important roles in pulmonary fibrosis through the PI3K/AKT, MAPK, and JAK/STAT signaling cascades, which involve EMT, cell proliferation, and differentiation. ZBM may partially prevent the development of pulmonary fibrosis by inhibiting these targets. Different targets were displayed in different colors. The blue arrows represented the interactions between the targets (promotion), and the red arrows represented the regulation (inhibition) of the target by ZBM.

In summary, the use of ZBM in the management of IPF may be based on a multitarget and multipathway approach.  Figure 12 presents a general illustration of this approach. These pathways are interconnected and synergistically influence the overall development of IPF. However, our study only tentatively proposed the partial mechanism of ZBM against IPF on the basis of computational predictions from network pharmacology analyses. Other unproven or relevant targets may also regulate IPF after administration of ZBM. Therefore, in vivo experiments, gene silencing, and other experimental methods should be considered in future studies to comprehensively describe the mechanisms of ZBM against IPF. This study has laid the foundation for further clinical applications of ZBM and the development of related therapeutic products.

## 5. Conclusions

In this study, we used a systematic analysis strategy based on network pharmacology to predict the potential mechanisms underlying the effects of ZBM against IPF. Our results suggested that ZBM improves IPF partly by influencing the trans-differentiation of fibroblasts to myofibroblasts through multiple targets such as EGRF, AKT1, MAPK1, SRC, PIK3CA, MAPK3, and JAK3 and multiple pathways such as the PI3K/AKT, MAPK, and JAK/STAT signaling pathways. This study provided new insights for elucidating the mechanism of TCM against IPF and will facilitate the further development of drugs related to ZBM.

## Figures and Tables

**Table 1:** Gene names and corresponding PDB IDs.

Gene names	PDB IDs
AKT1	3QKM
EGFR	7JXQ
GSK3B	4J71
JAK3	5TTV
MAPK1	6G9M
MAPK3	4QTB
MDM2	4ZFI
PIK3CA	7R9Y
SRC	2BDJ

**Table 2:** Sequences of primers used in quantitative real-time polymerase chain reaction analysis.

Gene names	Forward primers	Reverse primers
AKT1	GCTTCTTTGCCGGTATCGTG	TCCACACACTCCATGCTGTC
EGFR	CCATCCAAACTGCACCTACG	ACACGCTGCCATCATTACTTTG
GSK3B	CAAATGGGCGAGACACACCT	GGCATTTGTGGGGGTTGAAG
JAK3	GTCGTACCGGCATCTCGTG	TAGGCCAGCTGTTTGACCAC
MAPK1	GACCTACTGCCAGAGAACCC	TTGCTCGATGGTTGGTGCT
MAPK3	CCCCAAGTCAGACTCCAAAGC	TCCTTAGGTAGGTCATCCAGC
MDM2	GGCAGGGGAGAGTGATACAGA	GAAGCCAATTCTCACGAAGGG
PIK3CA	TAGGCAAGTCGAGGCAATGG	CTGGTCGCCTCATTTGCTCA
SRC	ACAACACAGAGGGAGACTGG	CACGTAGTTGCTGGGGATGT
ACTA2	CTCCGGAGCGCAAATACTCT	CCCGGCTTCATCGTATTCCT
COL1A2	GAGGGCAACAGCAGGTTCACTTA	TCAGCACCACCGATGTCCAA
COL3A1	AAGTCAAGGAGAAAGTGGTCG	CTCGTTCTCCATTCTTACCAGG
GAPDH	CAAATTCCATGGCACCGTCA	GACTCCACGACGTACTCAGC

**Table 3:** Docking affinity scores for compounds and targets.

Docking affinity scores	PubChem6325146	Ent-kauran-16, 17-diol	Kaempferol	Pelargonidin	Picropodophyllin
AKT1	−8.0	−7.7	−7.5	−7.5	−7.7
EGFR	−8.4	−7.4	−8.8	−8.9	−7.6
GSK3B	−8.0	−7.9	−7.6	−7.1	−8.0
JAK3	−7.6	−7.0	−8.4	−8.3	−8.9
MAPK1	−7.5	−8.1	−7.3	−7.2	−7.7
MAPK3	−8.5	−8.0	−9.1	−9.1	−7.9
MDM2	−6.5	−6.8	−6.7	−6.9	−6.8
PIK3CA	−6.6	−6.1	−9.0	−8.2	−7.9
SRC	−7.8	−8.8	−9.6	−9.0	−7.8

## Data Availability

The data used to support the findings of this study are available from the corresponding author upon request.

## Supplementary Materials

Supplementary Materials Five supplementary materials were combined to a single file named “Supporting Information” (including Supplementary Material S1-Chemical Composition Library of ZBM; Supplementary Material S2-Library of Compounds with Superior Properties; Supplementary Material S3-Compounds with Superior Properties and Their Corresponding Targets; Supplementary Material S4-Details of Intersection Targets; Supplementary Material S5-Information regarding the Crucial Gene Cluster).
