# Supplementary material for: Systematic Analysis Strategy Based on Network Pharmacology to Investigate the Potential Mechanism of Fritillaria thunbergii Miq. against Idiopathic Pulmonary Fibrosis
Source: Evid Based Complement Alternat Med. 2022 Nov 28;2022:2996878. doi: 10.1155/2022/2996878 (PMC9722288; doi:10.1155/2022/2996878)
Supplement: Supplementary Materials — Five supplementary materials were combined to a single file named “Supporting Information” (including Supplementary Material S1-Chemical Composition Library of ZBM; Supplementary Material S2-Library of Compounds with Superior Properties; Supplementary Material S3-Compounds with Superior Properties and Their Corresponding Targets; Supplementary Material S4-Details of Intersection Targets; Supplementary Material S5-Information regarding the Crucial Gene Cluster). [file 2996878.f1.docx]

**Supporting information**

**Systematic analysis strategy based on network pharmacology to investigate the potential mechanism of** ***Fritillaria thunbergii* Miq. against idiopathic pulmonary fibrosis**

Gonghao Xu^1^, Siwen Feng^1^, Rui Sun^1^, Qi Ding^2*^, and Yuanyuan Shi^2,3*^

^1^ School of Life Sciences, Beijing University of Chinese Medicine, Beijing 100029, China.
^2^ Shenzhen Research Institute, Beijing University of Chinese Medicine, Shenzhen 518118, China.

^3^ School of Chinese Materia Medica, Beijing University of Chinese Medicine, Beijing 100029, China.

Correspondence

Qi Ding, Shenzhen Research Institute, Beijing University of Chinese Medicine. Shenzhen 518118, China. Email: dingqisc@163.com.

Yuanyuan Shi, Shenzhen Research Institute, Beijing University of Chinese Medicine. Shenzhen 518118, China. Email: yshi@bucm.edu.cn

***Supplementary Material***

**Table S1** **Chemical Composition Library of ZBM**

| **Number** | **Compound name** | **Molecular Formula** | **Molecular Weight** | **PubChem CID** |
| --- | --- | --- | --- | --- |
| 1 | Solatubin | C_27_H_43_NO | 397.6 | 65727 |
| 2 | Ebeinone | C_27_H_41_NO_2_ | 411.6 | 180322 |
| 3 | Cyclopamine | C_27_H_41_NO_2_ | 411.6 | 442972 |
| 4 | Puqiedinone | C_27_H_43_NO_2_ | 413.6 | 126149 |
| 5 | Ziebeimine | C_27_H_43_NO_2_ | 413.6 | 3083151 |
| 6 | Ebeiedinone | C_27_H_43_NO_2_ | 413.6 | 5316984 |
| 7 | ZINC70688102 | C_27_H_43_NO_2_ | 413.6 | 5317396 |
| 8 | Zhebeirine | C_27_H_43_NO_2_ | 413.6 | 10597859 |
| 9 | Ebeiedine | C_27_H_45_NO_2_ | 415.7 | 101324888 |
| 10 | N-demethylpuqietinone | C_27_H_45_NO_2_ | 415.7 | 11304576 |
| 11 | Puqiedine | C_27_H_45_NO_2_ | 415.7 | 101400593 |
| 12 | Peimisine | C_27_H_41_NO_3_ | 427.6 | 161294 |
| 13 | Peiminine | C_27_H_43_NO_3_ | 429.6 | 167691 |
| 14 | Suchengbeisine | C_27_H_43_NO_3_ | 429.6 | 102112537 |
| 15 | Hapepunine | C_28_H_47_NO_2_ | 429.7 | 78407192 |
| 16 | Puqietinone | C_28_H_47_NO_2_ | 429.7 | 10693900 |
| 17 | Verticine | C_27_H_45_NO_3_ | 431.7 | 131900 |
| 18 | Zhebeinine | C_27_H_45_NO_3_ | 431.7 | 21121503 |
| 19 | Isoverticine | C_27_H_45_NO_3_ | 431.7 | 21573744 |
| 20 | Puqienine B | C_28_H_45_NO_3_ | 443.7 | 11419389 |
| 21 | Chaksine | C_22_H_38_N_6_O_4_ | 450.7 | 120699 |
| 22 | Peiminoside | C_33_H_55_NO_7_ | 577.8 | 90479565 |
| 23 | Edpetiline | C_33_H_53_NO_8_ | 591.8 | 90479257 |
| 24 | Apicidin | C_34_H_49_N_5_O_6_ | 623.8 | 6918328 |
| 25 | β1-chaconine | C_39_H_63_NO_10_ | 705.9 | 12302820 |
| 26 | Choline | C_5_H_14_NO^+^ | 104.2 | 305 |
| 27 | Thymine | C_5_H_6_N_2_O_2_ | 126.1 | 1135 |
| 28 | Thymidine | C_10_H_14_N_2_O_5_ | 242.2 | 5789 |
| 29 | Adenosine | C_10_H_13_N_5_O_4_ | 267.2 | 60961 |
| 30 | Pelargonidin | C_15_H_11_O_5_^+^ | 271.2 | 440832 |
| 31 | Pelargonidin-3,5-diglucoside | C_27_H_31_ClO_15_ | 631.0 | 167642 |
| 32 | 13-hydroxy-9E,11E-octadecadienoic acid | C_18_H_32_O_3_ | 296.4 | 5282948 |
| 33 | Coriolic acid | C_18_H_32_O_3_ | 296.4 | 5282947 |
| 34 | α-dimorphecolic acid | C_18_H_32_O_3_ | 296.4 | 5312830 |
| 35 | Kaempferol | C_15_H_10_O_6_ | 286.2 | 5280863 |
| 36 | Quercetin | C_15_H_10_O_7_ | 302.2 | 5280343 |
| 37 | Zhepeiresinol | C_14_H_16_O_6_ | 280.3 | 192547 |
| 38 | Picropodophyllin | C_22_H_22_O_8_ | 414.4 | 72435 |
| 39 | Syringaresinol | C_22_H_26_O_8_ | 418.4 | 100067 |
| 40 | Bayogenin | C_30_H_48_O_5_ | 488.7 | 12305221 |
| 41 | Bayogenin methyl ester | C_31_H_50_O_5_ | 502.7 | 21594191 |
| 42 | Polyphyllin V | C_39_H_62_O_12_ | 722.9 | 101377612 |
| 43 | β-sitosterol | C_29_H_50_O | 414.7 | 222284 |
| 44 | Carotene | C_40_H_56_ | 536.9 | 6419725 |
| 45 | Daucosterol | C_35_H_60_O_6_ | 576.8 | 5742590 |
| 46 | Ent-17-norkauran-16-one | C_19_H_30_O | 274.4 | 12740861 |
| 47 | (9'R)-5',5',9'-trimethylspiro[oxirane-2,14'-tetracyclo[11.2.1.01,10.04,9]hexadecane] | C_20_H_32_O | 288.5 | 9900592 |
| 48 | Ent-kaur-15-en-17-ol | C_20_H_32_O | 288.5 | 3082069 |
| 49 | Ent-(16s)-atisan-13,17-oxide | C_20_H_32_O | 288.5 | 6325926 |
| 50 | Trans-communol | C_20_H_32_O | 288.5 | 12303806 |
| 51 | Akhdarenol | C_20_H_32_O | 288.5 | 158539 |
| 52 | [(4R,9R,10S,14R,16R)-5,5,9-trimethyl-15-oxapentacyclo[11.3.1.01,10.04,9.014,16]heptadecan-14-yl]methanol | C_20_H_32_O_2_ | 304.5 | 6325146 |
| 53 | Ent-kauran-16,17-diol | C_20_H_34_O_2_ | 306.5 | 13816757 |
| 54 | Methyl cis-Communate | C_21_H_32_O_2_ | 316.5 | 11141639 |
| 55 | [(4R,9R,10R,14S)-14-methoxy-5,5,9-trimethyl-14-tetracyclo[11.2.1.01,10.04,9]hexadecanyl]methanol | C_21_H_36_O_2_ | 320.5 | 5319461 |
| 56 | 2,5-dimethoxybenzoquinone | C_8_H_8_O_4_ | 168.1 | 101405 |
| 57 | 3-Acetyl-5-hydroxy-7-methoxy-2-methyl-1,4-naphthoquinone | C_14_H_12_O_5_ | 260.2 | 67877406 |
| 58 | 6-methoxyl-2-acetyl-3-methyl-1,4-naphthoquinone-8-O-beta-D-glucopyranoside | C_20_H_22_O_10_ | 422.4 | 5319462 |
| 59 | δ-elemene | C_15_H_24_ | 204.4 | 12309449 |
| 60 | δ-selinene | C_15_H_24_ | 204.4 | 520383 |
| 61 | (Z)-tetradec-9-enal | C_14_H_26_O | 210.4 | 5364471 |
| 62 | Butylated hydroxytoluene | C_15_H_24_O | 220.4 | 31404 |
| 63 | Tetradecanoic acid | C_14_H_28_O_2_ | 228.4 | 11005 |
| 64 | Pentadecanoic acid | C_15_H_30_O_2_ | 242.4 | 13849 |
| 65 | (E)-hexadec-9-enoic acid | C_16_H_30_O_2_ | 254.4 | 5282745 |
| 66 | N-hexadecanoic acid | C_16_H_32_O_2_ | 256.4 | 985 |
| 67 | methyl hexadecanoate | C_17_H_34_O_2_ | 270.5 | 8181 |
| 68 | Heptadecanoic acid | C_17_H_34_O_2_ | 270.5 | 10465 |
| 69 | Kaur-15-ene | C20H32 | 272.5 | 521318 |
| 70 | Kaurene | C_20_H_32_ | 272.5 | 5318786 |
| 71 | Kaur-16-ene | C_20_H_32_O | 272.5 | 520687 |
| 72 | Podocarp-7-en-3-one, 13,13-dimethyl- | C_19_H_30_O | 274.4 | 621255 |
| 73 | (9E,12E)-octadeca-9,12-dienoic acid | C_18_H_32_O_2_ | 280.5 | 5282457 |
| 74 | Oleic acid | C_18_H_34_O_2_ | 282.5 | 445639 |
| 75 | Ethyl 9-hexadecenoate | C_18_H_34_O_2_ | 282.5 | 5364759 |
| 76 | ethyl hexadecanoate | C_18_H_36_O_2_ | 284.5 | 12366 |
| 77 | Testosterone | C^19^H^28^O^2^ | 288.4 | 6013 |
| 78 | 3-Methyleneandrostan-17-ol | C_20_H_32_O | 288.5 | 625647 |
| 79 | Pimara-7,15-dien-3-ol | C_20_H_32_O | 288.5 | 620519 |
| 80 | Manool oxide | C_20_H_34_O | 290.5 | 518574 |
| 81 | methyl (9Z,12Z)-octadeca-9,12-dienoate | C_19_H_34_O_2_ | 294.5 | 5284421 |
| 82 | 9,12-Octadecadienoic acid,methyl ester, (E, E)- | C_19_H_34_O_2_ | 294.5 | 8203 |
| 83 | Methyl-9,11-Octadecadienoate | C_19_H_34_O_2_ | 294.5 | 5365686 |
| 84 | Linoleic acid, ethyl ester | C_20_H_36_O_2_ | 308.5 | 5282184 |
| 85 | ethyl octadecanoate | C_20_H_40_O_2_ | 312.5 | 8122 |
| 86 | L-(+)-Ascorbic acid 2,6-dihexadecanoate | C_38_H_68_O_8_ | 653.0 | 54722209 |

***Supplementary Material***

**Table S2** **Library of Compounds with Superior Properties**

| **Number** | **Compound name** | **PubChem CID** | **SMILES** | **SwissADME** |
| --- | --- | --- | --- | --- |
| 1 | Peimisine | 161294 | CC1CC2C(C(C3(O2)CCC4C5CC(=O)C6CC(CCC6(C5CC4=C3C)C)O)C)NC1 | **Pharmacokinetics**  GI absorption High  BBB permeant Yes  P-gp substrate Yes  CYP1A2 inhibitor No  CYP2C19 inhibitor No  CYP2C9 inhibitor No  CYP2D6 inhibitor No  CYP3A4 inhibitor No  Log Kp (skin permeation) -6.98 cm/s  **Druglikeness**  Lipinski Yes; 0 violation  Ghose No; 1 violation: #atoms>70  Veber Yes  Egan Yes  Muegge Yes  Bioavailability Score 0.55 |
| 2 | Peiminine | 167691 | CC1CCC2C(C3CCC4C(C3CN2C1)CC5C4CC(=O)C6C5(CCC(C6)O)C)(C)O | **Pharmacokinetics**  GI absorption High  BBB permeant Yes  P-gp substrate Yes  CYP1A2 inhibitor No  CYP2C19 inhibitor No  CYP2C9 inhibitor No  CYP2D6 inhibitor No  CYP3A4 inhibitor No  Log Kp (skin permeation) -6.19 cm/s  **Druglikeness**  Lipinski Yes; 0 violation  Ghose No; 1 violation: #atoms>70  Veber Yes  Egan Yes  Muegge Yes  Bioavailability Score 0.55 |
| 3 | Suchengbeisine | 102112537 | CC1CCC(NC1)C2COC3(C2CCC4C3CC5C4CC(=O)C6C5(CCC(C6)O)C)C | **Pharmacokinetics**  GI absorption High  BBB permeant Yes  P-gp substrate Yes  CYP1A2 inhibitor No  CYP2C19 inhibitor No  CYP2C9 inhibitor No  CYP2D6 inhibitor No  CYP3A4 inhibitor No  Log Kp (skin permeation) -6.27 cm/s  **Druglikeness**  Lipinski Yes; 0 violation  Ghose No; 1 violation: #atoms>70  Veber Yes  Egan Yes  Muegge Yes  Bioavailability Score 0.55 |
| 4 | Verticine | 131900 | CC1CCC2C(C3CCC4C(C3CN2C1)CC5C4CC(C6C5(CCC(C6)O)C)O)(C)O | **Pharmacokinetics**  GI absorption High  BBB permeant Yes  P-gp substrate Yes  CYP1A2 inhibitor No  CYP2C19 inhibitor No  CYP2C9 inhibitor No  CYP2D6 inhibitor No  CYP3A4 inhibitor No  Log Kp (skin permeation) -6.00 cm/s  **Druglikeness**  Lipinski Yes; 0 violation  Ghose No; 1 violation: #atoms>70  Veber Yes  Egan Yes  Muegge Yes  Bioavailability Score 0.55 |
| 5 | Zhebeinine | 21121503 | CC1CCC2C(C3CCC4C(C3CN2C1)CC5C4CC(C6C5(CCC(C6)O)C)O)(C)O | **Pharmacokinetics**  GI absorption High  BBB permeant Yes  P-gp substrate Yes  CYP1A2 inhibitor No  CYP2C19 inhibitor No  CYP2C9 inhibitor No  CYP2D6 inhibitor No  CYP3A4 inhibitor No  Log Kp (skin permeation) -6.00 cm/s  **Druglikeness**  Lipinski Yes; 0 violation  Ghose No; 1 violation: #atoms>70  Veber Yes  Egan Yes  Muegge Yes  Bioavailability Score 0.55 |
| 6 | Isoverticine | 21573744 | CC1CCC2C(C3CCC4C(C3CN2C1)CC5C4CC(C6C5(CCC(C6)O)C)O)(C)O | **Pharmacokinetics**  GI absorption High  BBB permeant Yes  P-gp substrate Yes  CYP1A2 inhibitor No  CYP2C19 inhibitor No  CYP2C9 inhibitor No  CYP2D6 inhibitor No  CYP3A4 inhibitor No  Log Kp (skin permeation) -6.00 cm/s  **Druglikeness**  Lipinski Yes; 0 violation  Ghose No; 1 violation: #atoms>70  Veber Yes  Egan Yes  Muegge Yes  Bioavailability Score 0.55 |
| 7 | Puqienine B | 11419389 | CC1CC(C(N(C1)C)C(C)C2CCC3C4CC(=O)C5CC(CCC5(C4CC3=C2C)C)O)O | **Pharmacokinetics**  GI absorption High  BBB permeant Yes  P-gp substrate Yes  CYP1A2 inhibitor No  CYP2C19 inhibitor No  CYP2C9 inhibitor No  CYP2D6 inhibitor No  CYP3A4 inhibitor No  Log Kp (skin permeation) -6.42 cm/s  **Druglikeness**  Lipinski Yes; 0 violation  Ghose No; 2 violations: MR>130, #atoms>70  Veber Yes  Egan Yes  Muegge Yes  Bioavailability Score 0.55 |
| 8 | Thymidine | 5789 | CC1=CN(C(=O)NC1=O)C2CC(C(O2)CO)O | **Pharmacokinetics**  GI absorption High  BBB permeant No  P-gp substrate No  CYP1A2 inhibitor No  CYP2C19 inhibitor No  CYP2C9 inhibitor No  CYP2D6 inhibitor No  CYP3A4 inhibitor No  Log Kp (skin permeation) -8.61 cm/s  **Druglikeness**  Lipinski Yes; 0 violation  Ghose No; 1 violation: WLOGP<-0.4  Veber Yes  Egan Yes  Muegge Yes  Bioavailability Score 0.55 |
| 9 | Pelargonidin | 440832 | C1=CC(=CC=C1C2=[O+]C3=CC(=CC(=C3C=C2O)O)O)O | **Pharmacokinetics**  GI absorption High  BBB permeant No  P-gp substrate Yes  CYP1A2 inhibitor Yes  CYP2C19 inhibitor No  CYP2C9 inhibitor No  CYP2D6 inhibitor Yes  CYP3A4 inhibitor No  Log Kp (skin permeation) -6.33 cm/s  **Druglikeness**  Lipinski Yes; 0 violation  Ghose Yes  Veber Yes  Egan Yes  Muegge Yes  Bioavailability Score 0.55 |
| 10 | Kaempferol | 5280863 | C1=CC(=CC=C1C2=C(C(=O)C3=C(C=C(C=C3O2)O)O)O)O | **Pharmacokinetics**  GI absorption High  BBB permeant No  P-gp substrate No  CYP1A2 inhibitor Yes  CYP2C19 inhibitor No  CYP2C9 inhibitor No  CYP2D6 inhibitor Yes  CYP3A4 inhibitor Yes  Log Kp (skin permeation) -6.70 cm/s  **Druglikeness**  Lipinski Yes; 0 violation  Ghose Yes  Veber Yes  Egan Yes  Muegge Yes  Bioavailability Score 0.55 |
| 11 | Quercetin | 5280343 | C1=CC(=C(C=C1C2=C(C(=O)C3=C(C=C(C=C3O2)O)O)O)O)O | **Pharmacokinetics**  GI absorption High  BBB permeant No  P-gp substrate No  CYP1A2 inhibitor Yes  CYP2C19 inhibitor No  CYP2C9 inhibitor No  CYP2D6 inhibitor Yes  CYP3A4 inhibitor Yes  Log Kp (skin permeation) -7.05 cm/s  **Druglikeness**  Lipinski Yes; 0 violation  Ghose Yes  Veber Yes  Egan Yes  Muegge Yes  Bioavailability Score 0.55 |
| 12 | Zhepeiresinol | 192547 | COC1=CC(=CC(=C1O)OC)C2C3COC(=O)C3CO2 | **Pharmacokinetics**  GI absorption High  BBB permeant No  P-gp substrate No  CYP1A2 inhibitor No  CYP2C19 inhibitor No  CYP2C9 inhibitor No  CYP2D6 inhibitor No  CYP3A4 inhibitor No  Log Kp (skin permeation) -7.40 cm/s  **Druglikeness**  Lipinski Yes; 0 violation  Ghose Yes  Veber Yes  Egan Yes  Muegge Yes  Bioavailability Score 0.55 |
| 13 | Picropodophyllin | 72435 | COC1=CC(=CC(=C1OC)OC)C2C3C(COC3=O)C(C4=CC5=C(C=C24)OCO5)O | **Pharmacokinetics**  GI absorption High  BBB permeant No  P-gp substrate No  CYP1A2 inhibitor No  CYP2C19 inhibitor No  CYP2C9 inhibitor No  CYP2D6 inhibitor Yes  CYP3A4 inhibitor Yes  Log Kp (skin permeation) -7.40 cm/s  **Druglikeness**  Lipinski Yes; 0 violation  Ghose Yes  Veber Yes  Egan Yes  Muegge Yes  Bioavailability Score 0.55 |
| 14 | Syringaresinol | 100067 | COC1=CC(=CC(=C1O)OC)C2C3COC(C3CO2)C4=CC(=C(C(=C4)OC)O)OC | **Pharmacokinetics**  GI absorption High  BBB permeant No  P-gp substrate Yes  CYP1A2 inhibitor No  CYP2C19 inhibitor No  CYP2C9 inhibitor No  CYP2D6 inhibitor Yes  CYP3A4 inhibitor No  Log Kp (skin permeation) -7.27 cm/s  **Druglikeness**  Lipinski Yes; 0 violation  Ghose Yes  Veber Yes  Egan Yes  Muegge Yes  Bioavailability Score 0.55 |
| 15 | [(4R,9R,10S,14R,16R)-5,5,9-trimethyl-15-oxapentacyclo[11.3.1.01,10.04,9.014,16]heptadecan-14-yl]methanol | 6325146 | CC1(CCCC2(C1CCC34C2CCC(C3)C5(C4O5)CO)C)C | **Pharmacokinetics**  GI absorption High  BBB permeant Yes  P-gp substrate No  CYP1A2 inhibitor No  CYP2C19 inhibitor No  CYP2C9 inhibitor Yes  CYP2D6 inhibitor No  CYP3A4 inhibitor No  Log Kp (skin permeation) -4.81 cm/s  **Druglikeness**  Lipinski Yes; 0 violation  Ghose Yes  Veber Yes  Egan Yes  Muegge Yes  Bioavailability Score 0.55 |
| 16 | Ent-kauran-16,17-diol | 13816757 | CC1(CCCC2(C1CCC34C2CCC(C3)C(C4)(CO)O)C)C | **Pharmacokinetics**  GI absorption High  BBB permeant Yes  P-gp substrate No  CYP1A2 inhibitor No  CYP2C19 inhibitor No  CYP2C9 inhibitor Yes  CYP2D6 inhibitor No  CYP3A4 inhibitor No  Log Kp (skin permeation) -4.70 cm/s  **Druglikeness**  Lipinski Yes; 0 violation  Ghose Yes  Veber Yes  Egan Yes  Muegge Yes  Bioavailability Score 0.55 |
| 17 | Butylated hydroxytoluene | 31404 | CC1=CC(=C(C(=C1)C(C)(C)C)O)C(C)(C)C | **Pharmacokinetics**  GI absorption High  BBB permeant Yes  P-gp substrate No  CYP1A2 inhibitor No  CYP2C19 inhibitor No  CYP2C9 inhibitor No  CYP2D6 inhibitor Yes  CYP3A4 inhibitor No  Log Kp (skin permeation) -4.02 cm/s  **Druglikeness**  Lipinski Yes; 0 violation  Ghose Yes  Veber Yes  Egan Yes  Muegge No; 2 violations: XLOGP3>5, Heteroatoms<2  Bioavailability Score 0.55 |

***Supplementary Material***

**Table S3 Compounds with Superior Properties and Their Corresponding Targets**

| **Gene** | **Compound name** | **Source** |
| --- | --- | --- |
| SHH | Peimisine | Swisstarget prediction |
| SMO | Peimisine | Swisstarget prediction |
| EBP | Peimisine | Swisstarget prediction |
| ADRA1A | Peimisine | Swisstarget prediction |
| ADRA1B | Peimisine | Swisstarget prediction |
| ADRA1D | Peimisine | Swisstarget prediction |
| ADRA2A | Peimisine | Swisstarget prediction |
| ADRA2B | Peimisine | Swisstarget prediction |
| ADRA2C | Peimisine | Swisstarget prediction |
| ADRB3 | Peimisine | Swisstarget prediction |
| AKT1 | Peimisine | Swisstarget prediction |
| AKT2 | Peimisine | Swisstarget prediction |
| AKT3 | Peimisine | Swisstarget prediction |
| ALK | Peimisine | Swisstarget prediction |
| CCND1 CDK4 | Peimisine | Swisstarget prediction |
| CCNE2 CDK2 CCNE1 | Peimisine | Swisstarget prediction |
| CCR3 | Peimisine | Swisstarget prediction |
| CSF1R | Peimisine | Swisstarget prediction |
| CTSC | Peimisine | Swisstarget prediction |
| CYP24A1 | Peimisine | Swisstarget prediction |
| DPP4 | Peimisine | Swisstarget prediction |
| DRD2 | Peimisine | Swisstarget prediction |
| DRD3 | Peimisine | Swisstarget prediction |
| ESRRG | Peimisine | Swisstarget prediction |
| FKBP1A | Peimisine | Swisstarget prediction |
| HRH3 | Peimisine | Swisstarget prediction |
| HTR5A | Peimisine | Swisstarget prediction |
| HTR6 | Peimisine | Swisstarget prediction |
| HTR7 | Peimisine | Swisstarget prediction |
| INSR | Peimisine | Swisstarget prediction |
| JAK1 | Peimisine | Swisstarget prediction |
| JAK3 | Peimisine | Swisstarget prediction |
| KIT | Peimisine | Swisstarget prediction |
| MAP3K14 | Peimisine | Swisstarget prediction |
| NOS1 | Peimisine | Swisstarget prediction |
| NOS3 | Peimisine | Swisstarget prediction |
| PRKG1 | Peimisine | Swisstarget prediction |
| ROCK2 | Peimisine | Swisstarget prediction |
| TNK2 | Peimisine | Swisstarget prediction |
| HSD17B3 | Peiminine | Swisstarget prediction |
| BCHE | Peiminine | Swisstarget prediction |
| HSD11B1 | Peiminine | Swisstarget prediction |
| CHRM2 | Peiminine | Swisstarget prediction |
| CHRM1 | Peiminine | Swisstarget prediction |
| REN | Peiminine | Swisstarget prediction |
| LSS | Peiminine | Swisstarget prediction |
| SLC18A3 | Peiminine | Swisstarget prediction |
| UGCG | Peiminine | Swisstarget prediction |
| GBA | Peiminine | Swisstarget prediction |
| GBA2 | Peiminine | Swisstarget prediction |
| SRD5A2 | Peiminine | Swisstarget prediction |
| AR | Peiminine | Swisstarget prediction |
| SIGMAR1 | Peiminine | Swisstarget prediction |
| AKT2 | Peiminine | Swisstarget prediction |
| AKT1 | Peiminine | Swisstarget prediction |
| AKT3 | Peiminine | Swisstarget prediction |
| KISS1R | Peiminine | Swisstarget prediction |
| MME | Peiminine | Swisstarget prediction |
| EBP | Peiminine | Swisstarget prediction |
| PRKCA | Peiminine | Swisstarget prediction |
| ITGA2B ITGB3 | Peiminine | Swisstarget prediction |
| PRKG1 | Peiminine | Swisstarget prediction |
| DRD1 | Peiminine | Swisstarget prediction |
| INSR | Peiminine | Swisstarget prediction |
| ALK | Peiminine | Swisstarget prediction |
| PLA2G2C | Peiminine | Swisstarget prediction |
| PLA2G10 | Peiminine | Swisstarget prediction |
| CNR1 | Peiminine | Swisstarget prediction |
| TACR2 | Peiminine | Swisstarget prediction |
| SLC1A2 | Peiminine | Swisstarget prediction |
| ADRA1D | Peiminine | Swisstarget prediction |
| ADRA1A | Peiminine | Swisstarget prediction |
| PLA2G1B | Peiminine | Swisstarget prediction |
| ROCK2 | Peiminine | Swisstarget prediction |
| MCHR1 | Peiminine | Swisstarget prediction |
| MDM2 | Peiminine | Swisstarget prediction |
| PTGER4 | Peiminine | Swisstarget prediction |
| PTGER2 | Peiminine | Swisstarget prediction |
| PTGER3 | Peiminine | Swisstarget prediction |
| DRD4 | Peiminine | Swisstarget prediction |
| PRKCQ | Peiminine | Swisstarget prediction |
| HTR7 | Peiminine | Swisstarget prediction |
| HTR6 | Peiminine | Swisstarget prediction |
| MGAM | Peiminine | Swisstarget prediction |
| GLB1 | Peiminine | Swisstarget prediction |
| GAA | Peiminine | Swisstarget prediction |
| SI | Peiminine | Swisstarget prediction |
| NR1H4 | Peiminine | Swisstarget prediction |
| DPP4 | Peiminine | Swisstarget prediction |
| OPRL1 | Peiminine | Swisstarget prediction |
| ADRB2 | Peiminine | Swisstarget prediction |
| ADRB3 | Peiminine | Swisstarget prediction |
| CHRNA4 CHRNB2 | Peiminine | Swisstarget prediction |
| GPR88 | Peiminine | Swisstarget prediction |
| DNPEP | Peiminine | Swisstarget prediction |
| JAK3 | Peiminine | Swisstarget prediction |
| JAK1 | Peiminine | Swisstarget prediction |
| OPRD1 | Peiminine | Swisstarget prediction |
| CCR1 | Peiminine | Swisstarget prediction |
| CCR3 | Peiminine | Swisstarget prediction |
| NR3C1 | Peiminine | Swisstarget prediction |
| MAN2B1 | Peiminine | Swisstarget prediction |
| PDE10A | Peiminine | Swisstarget prediction |
| APP | Peiminine | Swisstarget prediction |
| CHRM1 | Suchengbeisine | Swisstarget prediction |
| CHRM2 | Suchengbeisine | Swisstarget prediction |
| CHRM3 | Suchengbeisine | Swisstarget prediction |
| CHRM4 | Suchengbeisine | Swisstarget prediction |
| PPP5C | Suchengbeisine | Swisstarget prediction |
| PPP1CA | Suchengbeisine | Swisstarget prediction |
| AKT2 | Suchengbeisine | Swisstarget prediction |
| AKT1 | Suchengbeisine | Swisstarget prediction |
| AKT3 | Suchengbeisine | Swisstarget prediction |
| BCHE | Suchengbeisine | Swisstarget prediction |
| CHRM5 | Suchengbeisine | Swisstarget prediction |
| ATP12A | Suchengbeisine | Swisstarget prediction |
| IRAK1 | Suchengbeisine | Swisstarget prediction |
| IRAK4 | Suchengbeisine | Swisstarget prediction |
| MAP3K7 | Suchengbeisine | Swisstarget prediction |
| SLC6A2 | Suchengbeisine | Swisstarget prediction |
| SLC6A4 | Suchengbeisine | Swisstarget prediction |
| SLC6A3 | Suchengbeisine | Swisstarget prediction |
| SHH | Suchengbeisine | Swisstarget prediction |
| ESRRG | Suchengbeisine | Swisstarget prediction |
| MGAM | Suchengbeisine | Swisstarget prediction |
| GAA | Suchengbeisine | Swisstarget prediction |
| SI | Suchengbeisine | Swisstarget prediction |
| AGL | Suchengbeisine | Swisstarget prediction |
| HSD17B7 | Suchengbeisine | Swisstarget prediction |
| HTR3A | Suchengbeisine | Swisstarget prediction |
| ADRA1B | Suchengbeisine | Swisstarget prediction |
| PRKG1 | Suchengbeisine | Swisstarget prediction |
| PRKCB | Suchengbeisine | Swisstarget prediction |
| TNK2 | Suchengbeisine | Swisstarget prediction |
| SCN9A | Suchengbeisine | Swisstarget prediction |
| CTSG | Suchengbeisine | Swisstarget prediction |
| MTOR | Suchengbeisine | Swisstarget prediction |
| TTK | Suchengbeisine | Swisstarget prediction |
| PIK3CA | Suchengbeisine | Swisstarget prediction |
| HRH3 | Suchengbeisine | Swisstarget prediction |
| SSTR2 | Suchengbeisine | Swisstarget prediction |
| MAPK1 | Suchengbeisine | Swisstarget prediction |
| CYP2J2 | Suchengbeisine | Swisstarget prediction |
| TACR1 | Suchengbeisine | Swisstarget prediction |
| DRD4 | Suchengbeisine | Swisstarget prediction |
| IGF1R | Suchengbeisine | Swisstarget prediction |
| MC4R | Suchengbeisine | Swisstarget prediction |
| PRKCD | Suchengbeisine | Swisstarget prediction |
| PRKCQ | Suchengbeisine | Swisstarget prediction |
| MC5R | Suchengbeisine | Swisstarget prediction |
| MC3R | Suchengbeisine | Swisstarget prediction |
| NPY1R | Suchengbeisine | Swisstarget prediction |
| MAP3K14 | Suchengbeisine | Swisstarget prediction |
| JAK3 | Suchengbeisine | Swisstarget prediction |
| JAK1 | Suchengbeisine | Swisstarget prediction |
| MDM2 | Suchengbeisine | Swisstarget prediction |
| XIAP | Suchengbeisine | Swisstarget prediction |
| EGLN1 | Suchengbeisine | Swisstarget prediction |
| EBP | Suchengbeisine | Swisstarget prediction |
| GBA | Suchengbeisine | Swisstarget prediction |
| INSR | Suchengbeisine | Swisstarget prediction |
| ALK | Suchengbeisine | Swisstarget prediction |
| PARP1 | Suchengbeisine | Swisstarget prediction |
| RPS6KB1 | Suchengbeisine | Swisstarget prediction |
| IDH1 | Suchengbeisine | Swisstarget prediction |
| PDE10A | Suchengbeisine | Swisstarget prediction |
| CREBBP | Suchengbeisine | Swisstarget prediction |
| HTR1B | Suchengbeisine | Swisstarget prediction |
| HTR1D | Suchengbeisine | Swisstarget prediction |
| OPRL1 | Suchengbeisine | Swisstarget prediction |
| LSS | Verticine | Swisstarget prediction |
| SLC18A3 | Verticine | Swisstarget prediction |
| GAA | Verticine | Swisstarget prediction |
| GBA | Verticine | Swisstarget prediction |
| PLA2G1B | Verticine | Swisstarget prediction |
| SIGMAR1 | Verticine | Swisstarget prediction |
| EBP | Verticine | Swisstarget prediction |
| GANAB | Verticine | Swisstarget prediction |
| HSD17B3 | Verticine | Swisstarget prediction |
| FUCA1 | Verticine | Swisstarget prediction |
| FUCA2 | Verticine | Swisstarget prediction |
| GANC | Verticine | Swisstarget prediction |
| GLA | Verticine | Swisstarget prediction |
| GBA2 | Verticine | Swisstarget prediction |
| AKT2 | Verticine | Swisstarget prediction |
| AKT1 | Verticine | Swisstarget prediction |
| AKT3 | Verticine | Swisstarget prediction |
| DNM1 | Verticine | Swisstarget prediction |
| IRAK1 | Verticine | Swisstarget prediction |
| IRAK4 | Verticine | Swisstarget prediction |
| MAP3K7 | Verticine | Swisstarget prediction |
| UGCG | Verticine | Swisstarget prediction |
| MGAM | Verticine | Swisstarget prediction |
| SI | Verticine | Swisstarget prediction |
| BCHE | Verticine | Swisstarget prediction |
| MANBA | Verticine | Swisstarget prediction |
| DRD2 | Verticine | Swisstarget prediction |
| PLA2G2A | Verticine | Swisstarget prediction |
| GLB1 | Verticine | Swisstarget prediction |
| HSP90AA1 | Verticine | Swisstarget prediction |
| MC4R | Verticine | Swisstarget prediction |
| SSTR2 | Verticine | Swisstarget prediction |
| PRKG1 | Verticine | Swisstarget prediction |
| MAN2B1 | Verticine | Swisstarget prediction |
| MAOA | Verticine | Swisstarget prediction |
| JAK3 | Verticine | Swisstarget prediction |
| JAK1 | Verticine | Swisstarget prediction |
| ADRA1D | Verticine | Swisstarget prediction |
| ADRA1A | Verticine | Swisstarget prediction |
| CHRM1 | Verticine | Swisstarget prediction |
| CHRM3 | Verticine | Swisstarget prediction |
| SCN9A | Verticine | Swisstarget prediction |
| OPRL1 | Verticine | Swisstarget prediction |
| HTR2A | Verticine | Swisstarget prediction |
| PRKCD | Verticine | Swisstarget prediction |
| PRKCQ | Verticine | Swisstarget prediction |
| MC5R | Verticine | Swisstarget prediction |
| MC3R | Verticine | Swisstarget prediction |
| MDM2 | Verticine | Swisstarget prediction |
| AGL | Verticine | Swisstarget prediction |
| DRD1 | Verticine | Swisstarget prediction |
| MCHR1 | Verticine | Swisstarget prediction |
| ADRB2 | Verticine | Swisstarget prediction |
| ADRB3 | Verticine | Swisstarget prediction |
| PRKCB | Verticine | Swisstarget prediction |
| MELK | Verticine | Swisstarget prediction |
| SLC6A2 | Verticine | Swisstarget prediction |
| DRD4 | Verticine | Swisstarget prediction |
| CCR1 | Verticine | Swisstarget prediction |
| CCR3 | Verticine | Swisstarget prediction |
| NPY1R | Verticine | Swisstarget prediction |
| MAP3K14 | Verticine | Swisstarget prediction |
| RPS6KA3 | Verticine | Swisstarget prediction |
| RPS6KB1 | Verticine | Swisstarget prediction |
| SRC | Verticine | Swisstarget prediction |
| PIK3CD | Verticine | Swisstarget prediction |
| CHRM2 | Verticine | Swisstarget prediction |
| KDR | Verticine | Swisstarget prediction |
| TTK | Verticine | Swisstarget prediction |
| MAPK1 | Verticine | Swisstarget prediction |
| AURKA | Verticine | Swisstarget prediction |
| ROCK2 | Verticine | Swisstarget prediction |
| KCNH2 | Verticine | Swisstarget prediction |
| HTR1A | Verticine | Swisstarget prediction |
| PARP1 | Verticine | Swisstarget prediction |
| CCR2 | Verticine | Swisstarget prediction |
| LSS | Zhebeinine | Swisstarget prediction |
| SLC18A3 | Zhebeinine | Swisstarget prediction |
| GAA | Zhebeinine | Swisstarget prediction |
| GBA | Zhebeinine | Swisstarget prediction |
| PLA2G1B | Zhebeinine | Swisstarget prediction |
| SIGMAR1 | Zhebeinine | Swisstarget prediction |
| EBP | Zhebeinine | Swisstarget prediction |
| GANAB | Zhebeinine | Swisstarget prediction |
| HSD17B3 | Zhebeinine | Swisstarget prediction |
| FUCA1 | Zhebeinine | Swisstarget prediction |
| FUCA2 | Zhebeinine | Swisstarget prediction |
| GANC | Zhebeinine | Swisstarget prediction |
| GLA | Zhebeinine | Swisstarget prediction |
| GBA2 | Zhebeinine | Swisstarget prediction |
| AKT2 | Zhebeinine | Swisstarget prediction |
| AKT1 | Zhebeinine | Swisstarget prediction |
| AKT3 | Zhebeinine | Swisstarget prediction |
| DNM1 | Zhebeinine | Swisstarget prediction |
| IRAK1 | Zhebeinine | Swisstarget prediction |
| IRAK4 | Zhebeinine | Swisstarget prediction |
| MAP3K7 | Zhebeinine | Swisstarget prediction |
| UGCG | Zhebeinine | Swisstarget prediction |
| MGAM | Zhebeinine | Swisstarget prediction |
| SI | Zhebeinine | Swisstarget prediction |
| BCHE | Zhebeinine | Swisstarget prediction |
| MANBA | Zhebeinine | Swisstarget prediction |
| DRD2 | Zhebeinine | Swisstarget prediction |
| PLA2G2A | Zhebeinine | Swisstarget prediction |
| GLB1 | Zhebeinine | Swisstarget prediction |
| HSP90AA1 | Zhebeinine | Swisstarget prediction |
| MC4R | Zhebeinine | Swisstarget prediction |
| SSTR2 | Zhebeinine | Swisstarget prediction |
| PRKG1 | Zhebeinine | Swisstarget prediction |
| MAN2B1 | Zhebeinine | Swisstarget prediction |
| MAOA | Zhebeinine | Swisstarget prediction |
| JAK3 | Zhebeinine | Swisstarget prediction |
| JAK1 | Zhebeinine | Swisstarget prediction |
| ADRA1D | Zhebeinine | Swisstarget prediction |
| ADRA1A | Zhebeinine | Swisstarget prediction |
| CHRM1 | Zhebeinine | Swisstarget prediction |
| CHRM3 | Zhebeinine | Swisstarget prediction |
| SCN9A | Zhebeinine | Swisstarget prediction |
| OPRL1 | Zhebeinine | Swisstarget prediction |
| HTR2A | Zhebeinine | Swisstarget prediction |
| PRKCD | Zhebeinine | Swisstarget prediction |
| PRKCQ | Zhebeinine | Swisstarget prediction |
| MC5R | Zhebeinine | Swisstarget prediction |
| MC3R | Zhebeinine | Swisstarget prediction |
| MDM2 | Zhebeinine | Swisstarget prediction |
| AGL | Zhebeinine | Swisstarget prediction |
| DRD1 | Zhebeinine | Swisstarget prediction |
| MCHR1 | Zhebeinine | Swisstarget prediction |
| ADRB2 | Zhebeinine | Swisstarget prediction |
| ADRB3 | Zhebeinine | Swisstarget prediction |
| PRKCB | Zhebeinine | Swisstarget prediction |
| MELK | Zhebeinine | Swisstarget prediction |
| SLC6A2 | Zhebeinine | Swisstarget prediction |
| DRD4 | Zhebeinine | Swisstarget prediction |
| CCR1 | Zhebeinine | Swisstarget prediction |
| CCR3 | Zhebeinine | Swisstarget prediction |
| NPY1R | Zhebeinine | Swisstarget prediction |
| MAP3K14 | Zhebeinine | Swisstarget prediction |
| RPS6KA3 | Zhebeinine | Swisstarget prediction |
| RPS6KB1 | Zhebeinine | Swisstarget prediction |
| SRC | Zhebeinine | Swisstarget prediction |
| PIK3CD | Zhebeinine | Swisstarget prediction |
| CHRM2 | Zhebeinine | Swisstarget prediction |
| KDR | Zhebeinine | Swisstarget prediction |
| TTK | Zhebeinine | Swisstarget prediction |
| MAPK1 | Zhebeinine | Swisstarget prediction |
| AURKA | Zhebeinine | Swisstarget prediction |
| ROCK2 | Zhebeinine | Swisstarget prediction |
| KCNH2 | Zhebeinine | Swisstarget prediction |
| HTR1A | Zhebeinine | Swisstarget prediction |
| PARP1 | Zhebeinine | Swisstarget prediction |
| CCR2 | Zhebeinine | Swisstarget prediction |
| LSS | Isoverticine | Swisstarget prediction |
| SLC18A3 | Isoverticine | Swisstarget prediction |
| GAA | Isoverticine | Swisstarget prediction |
| GBA | Isoverticine | Swisstarget prediction |
| PLA2G1B | Isoverticine | Swisstarget prediction |
| SIGMAR1 | Isoverticine | Swisstarget prediction |
| EBP | Isoverticine | Swisstarget prediction |
| GANAB | Isoverticine | Swisstarget prediction |
| HSD17B3 | Isoverticine | Swisstarget prediction |
| FUCA1 | Isoverticine | Swisstarget prediction |
| FUCA2 | Isoverticine | Swisstarget prediction |
| GANC | Isoverticine | Swisstarget prediction |
| GLA | Isoverticine | Swisstarget prediction |
| GBA2 | Isoverticine | Swisstarget prediction |
| AKT2 | Isoverticine | Swisstarget prediction |
| AKT1 | Isoverticine | Swisstarget prediction |
| AKT3 | Isoverticine | Swisstarget prediction |
| DNM1 | Isoverticine | Swisstarget prediction |
| IRAK1 | Isoverticine | Swisstarget prediction |
| IRAK4 | Isoverticine | Swisstarget prediction |
| MAP3K7 | Isoverticine | Swisstarget prediction |
| UGCG | Isoverticine | Swisstarget prediction |
| MGAM | Isoverticine | Swisstarget prediction |
| SI | Isoverticine | Swisstarget prediction |
| BCHE | Isoverticine | Swisstarget prediction |
| MANBA | Isoverticine | Swisstarget prediction |
| DRD2 | Isoverticine | Swisstarget prediction |
| PLA2G2A | Isoverticine | Swisstarget prediction |
| GLB1 | Isoverticine | Swisstarget prediction |
| HSP90AA1 | Isoverticine | Swisstarget prediction |
| MC4R | Isoverticine | Swisstarget prediction |
| SSTR2 | Isoverticine | Swisstarget prediction |
| PRKG1 | Isoverticine | Swisstarget prediction |
| MAN2B1 | Isoverticine | Swisstarget prediction |
| MAOA | Isoverticine | Swisstarget prediction |
| JAK3 | Isoverticine | Swisstarget prediction |
| JAK1 | Isoverticine | Swisstarget prediction |
| ADRA1D | Isoverticine | Swisstarget prediction |
| ADRA1A | Isoverticine | Swisstarget prediction |
| CHRM1 | Isoverticine | Swisstarget prediction |
| CHRM3 | Isoverticine | Swisstarget prediction |
| SCN9A | Isoverticine | Swisstarget prediction |
| OPRL1 | Isoverticine | Swisstarget prediction |
| HTR2A | Isoverticine | Swisstarget prediction |
| PRKCD | Isoverticine | Swisstarget prediction |
| PRKCQ | Isoverticine | Swisstarget prediction |
| MC5R | Isoverticine | Swisstarget prediction |
| MC3R | Isoverticine | Swisstarget prediction |
| MDM2 | Isoverticine | Swisstarget prediction |
| AGL | Isoverticine | Swisstarget prediction |
| DRD1 | Isoverticine | Swisstarget prediction |
| MCHR1 | Isoverticine | Swisstarget prediction |
| ADRB2 | Isoverticine | Swisstarget prediction |
| ADRB3 | Isoverticine | Swisstarget prediction |
| PRKCB | Isoverticine | Swisstarget prediction |
| MELK | Isoverticine | Swisstarget prediction |
| SLC6A2 | Isoverticine | Swisstarget prediction |
| DRD4 | Isoverticine | Swisstarget prediction |
| CCR1 | Isoverticine | Swisstarget prediction |
| CCR3 | Isoverticine | Swisstarget prediction |
| NPY1R | Isoverticine | Swisstarget prediction |
| MAP3K14 | Isoverticine | Swisstarget prediction |
| RPS6KA3 | Isoverticine | Swisstarget prediction |
| RPS6KB1 | Isoverticine | Swisstarget prediction |
| SRC | Isoverticine | Swisstarget prediction |
| PIK3CD | Isoverticine | Swisstarget prediction |
| CHRM2 | Isoverticine | Swisstarget prediction |
| KDR | Isoverticine | Swisstarget prediction |
| TTK | Isoverticine | Swisstarget prediction |
| MAPK1 | Isoverticine | Swisstarget prediction |
| AURKA | Isoverticine | Swisstarget prediction |
| ROCK2 | Isoverticine | Swisstarget prediction |
| KCNH2 | Isoverticine | Swisstarget prediction |
| HTR1A | Isoverticine | Swisstarget prediction |
| PARP1 | Isoverticine | Swisstarget prediction |
| CCR2 | Isoverticine | Swisstarget prediction |
| OPRM1 | Puqienine B | Swisstarget prediction |
| OPRD1 | Puqienine B | Swisstarget prediction |
| SHH | Puqienine B | Swisstarget prediction |
| NPC1L1 | Puqienine B | Swisstarget prediction |
| EBP | Puqienine B | Swisstarget prediction |
| SLC6A2 | Puqienine B | Swisstarget prediction |
| OPRK1 | Puqienine B | Swisstarget prediction |
| SIGMAR1 | Puqienine B | Swisstarget prediction |
| CCR1 | Puqienine B | Swisstarget prediction |
| AKT2 | Puqienine B | Swisstarget prediction |
| CCR3 | Puqienine B | Swisstarget prediction |
| AKT1 | Puqienine B | Swisstarget prediction |
| AKT3 | Puqienine B | Swisstarget prediction |
| DRD1 | Puqienine B | Swisstarget prediction |
| DRD2 | Puqienine B | Swisstarget prediction |
| JAK3 | Puqienine B | Swisstarget prediction |
| JAK1 | Puqienine B | Swisstarget prediction |
| CNR1 | Puqienine B | Swisstarget prediction |
| PRKG1 | Puqienine B | Swisstarget prediction |
| MAOA | Puqienine B | Swisstarget prediction |
| PPARA | Puqienine B | Swisstarget prediction |
| GPR119 | Puqienine B | Swisstarget prediction |
| SRD5A2 | Puqienine B | Swisstarget prediction |
| MCHR1 | Puqienine B | Swisstarget prediction |
| HTR2A | Puqienine B | Swisstarget prediction |
| PRKCD | Puqienine B | Swisstarget prediction |
| PRKCQ | Puqienine B | Swisstarget prediction |
| CSF1R | Puqienine B | Swisstarget prediction |
| KIT | Puqienine B | Swisstarget prediction |
| ADRA2B | Puqienine B | Swisstarget prediction |
| ADRA1D | Puqienine B | Swisstarget prediction |
| ADRA1A | Puqienine B | Swisstarget prediction |
| ADRA1B | Puqienine B | Swisstarget prediction |
| ROCK2 | Puqienine B | Swisstarget prediction |
| IRAK1 | Puqienine B | Swisstarget prediction |
| IRAK4 | Puqienine B | Swisstarget prediction |
| MAP3K7 | Puqienine B | Swisstarget prediction |
| CCND1 CDK4 | Puqienine B | Swisstarget prediction |
| CCNE2 CDK2 CCNE1 | Puqienine B | Swisstarget prediction |
| ADRB2 | Puqienine B | Swisstarget prediction |
| ADRB3 | Puqienine B | Swisstarget prediction |
| TNK2 | Puqienine B | Swisstarget prediction |
| ESRRG | Puqienine B | Swisstarget prediction |
| CHEK1 | Puqienine B | Swisstarget prediction |
| CTSS | Puqienine B | Swisstarget prediction |
| NR1H3 | Puqienine B | Swisstarget prediction |
| PTPN1 | Puqienine B | Swisstarget prediction |
| TACR2 | Puqienine B | Swisstarget prediction |
| PRKCB | Puqienine B | Swisstarget prediction |
| HSD11B1 | Puqienine B | Swisstarget prediction |
| CCNE1 CDK2 | Puqienine B | Swisstarget prediction |
| ATP12A | Puqienine B | Swisstarget prediction |
| PIK3CD | Puqienine B | Swisstarget prediction |
| MDM2 | Puqienine B | Swisstarget prediction |
| MAP3K14 | Puqienine B | Swisstarget prediction |
| DGAT1 | Puqienine B | Swisstarget prediction |
| SSTR2 | Puqienine B | Swisstarget prediction |
| OPRL1 | Puqienine B | Swisstarget prediction |
| HTR7 | Puqienine B | Swisstarget prediction |
| HTR6 | Puqienine B | Swisstarget prediction |
| HTR5A | Puqienine B | Swisstarget prediction |
| XIAP | Puqienine B | Swisstarget prediction |
| CHRNA3 CHRNB2 | Puqienine B | Swisstarget prediction |
| SRC | Puqienine B | Swisstarget prediction |
| JAK2 | Puqienine B | Swisstarget prediction |
| PTGS1 | Thymidine | TCMSP |
| AR | Thymidine | TCMSP |
| PTGS2 | Thymidine | TCMSP |
| CA2 | Thymidine | TCMSP |
| PTPN1 | Thymidine | TCMSP |
| AKR1B1 | Thymidine | TCMSP |
| DPP4 | Thymidine | TCMSP |
| HSP90AA1 | Thymidine | TCMSP |
| PIK3CG | Thymidine | TCMSP |
| PRSS1 | Thymidine | TCMSP |
| NCOA2 | Thymidine | TCMSP |
| F2 | Pelargonidin | Swisstarget prediction |
| NR1H3 | Pelargonidin | Swisstarget prediction |
| GLO1 | Pelargonidin | Swisstarget prediction |
| NOX4 | Pelargonidin | Swisstarget prediction |
| AKR1B1 | Pelargonidin | Swisstarget prediction |
| XDH | Pelargonidin | Swisstarget prediction |
| TYR | Pelargonidin | Swisstarget prediction |
| FLT3 | Pelargonidin | Swisstarget prediction |
| CA2 | Pelargonidin | Swisstarget prediction |
| ALOX5 | Pelargonidin | Swisstarget prediction |
| CA7 | Pelargonidin | Swisstarget prediction |
| HSD17B2 | Pelargonidin | Swisstarget prediction |
| ABCC1 | Pelargonidin | Swisstarget prediction |
| HSD17B1 | Pelargonidin | Swisstarget prediction |
| AHR | Pelargonidin | Swisstarget prediction |
| CA12 | Pelargonidin | Swisstarget prediction |
| ESRRA | Pelargonidin | Swisstarget prediction |
| ABCB1 | Pelargonidin | Swisstarget prediction |
| CYP1B1 | Pelargonidin | Swisstarget prediction |
| ABCG2 | Pelargonidin | Swisstarget prediction |
| CD38 | Pelargonidin | Swisstarget prediction |
| BCHE | Pelargonidin | Swisstarget prediction |
| ACHE | Pelargonidin | Swisstarget prediction |
| ADORA1 | Pelargonidin | Swisstarget prediction |
| ADORA2A | Pelargonidin | Swisstarget prediction |
| ADORA3 | Pelargonidin | Swisstarget prediction |
| CA4 | Pelargonidin | Swisstarget prediction |
| GPR35 | Pelargonidin | Swisstarget prediction |
| PTPRS | Pelargonidin | Swisstarget prediction |
| ESR2 | Pelargonidin | Swisstarget prediction |
| DAPK1 | Pelargonidin | Swisstarget prediction |
| MPG | Pelargonidin | Swisstarget prediction |
| SLC22A12 | Pelargonidin | Swisstarget prediction |
| ALOX15 | Pelargonidin | Swisstarget prediction |
| SHBG | Pelargonidin | Swisstarget prediction |
| MAPT | Pelargonidin | Swisstarget prediction |
| KDM4E | Pelargonidin | Swisstarget prediction |
| AVPR2 | Pelargonidin | Swisstarget prediction |
| TOP2A | Pelargonidin | Swisstarget prediction |
| MAOA | Pelargonidin | Swisstarget prediction |
| IGF1R | Pelargonidin | Swisstarget prediction |
| CYP19A1 | Pelargonidin | Swisstarget prediction |
| INSR | Pelargonidin | Swisstarget prediction |
| EGFR | Pelargonidin | Swisstarget prediction |
| PIM1 | Pelargonidin | Swisstarget prediction |
| AURKB | Pelargonidin | Swisstarget prediction |
| DRD4 | Pelargonidin | Swisstarget prediction |
| MYLK | Pelargonidin | Swisstarget prediction |
| MPO | Pelargonidin | Swisstarget prediction |
| PIK3R1 | Pelargonidin | Swisstarget prediction |
| PYGL | Pelargonidin | Swisstarget prediction |
| SYK | Pelargonidin | Swisstarget prediction |
| CA1 | Pelargonidin | Swisstarget prediction |
| GSK3B | Pelargonidin | Swisstarget prediction |
| SRC | Pelargonidin | Swisstarget prediction |
| PTK2 | Pelargonidin | Swisstarget prediction |
| KDR | Pelargonidin | Swisstarget prediction |
| MMP13 | Pelargonidin | Swisstarget prediction |
| MMP3 | Pelargonidin | Swisstarget prediction |
| CA3 | Pelargonidin | Swisstarget prediction |
| PLK1 | Pelargonidin | Swisstarget prediction |
| CA6 | Pelargonidin | Swisstarget prediction |
| CDK1 | Pelargonidin | Swisstarget prediction |
| MMP9 | Pelargonidin | Swisstarget prediction |
| PIK3CG | Pelargonidin | Swisstarget prediction |
| MMP2 | Pelargonidin | Swisstarget prediction |
| PKN1 | Pelargonidin | Swisstarget prediction |
| CA14 | Pelargonidin | Swisstarget prediction |
| CA9 | Pelargonidin | Swisstarget prediction |
| CSNK2A1 | Pelargonidin | Swisstarget prediction |
| ALOX12 | Pelargonidin | Swisstarget prediction |
| MET | Pelargonidin | Swisstarget prediction |
| NEK2 | Pelargonidin | Swisstarget prediction |
| CXCR1 | Pelargonidin | Swisstarget prediction |
| CAMK2B | Pelargonidin | Swisstarget prediction |
| ALK | Pelargonidin | Swisstarget prediction |
| AKT1 | Pelargonidin | Swisstarget prediction |
| NEK6 | Pelargonidin | Swisstarget prediction |
| PLA2G1B | Pelargonidin | Swisstarget prediction |
| CA5A | Pelargonidin | Swisstarget prediction |
| BACE1 | Pelargonidin | Swisstarget prediction |
| AXL | Pelargonidin | Swisstarget prediction |
| APEX1 | Pelargonidin | Swisstarget prediction |
| NUAK1 | Pelargonidin | Swisstarget prediction |
| AKR1C2 | Pelargonidin | Swisstarget prediction |
| AKR1C1 | Pelargonidin | Swisstarget prediction |
| AKR1C3 | Pelargonidin | Swisstarget prediction |
| AKR1C4 | Pelargonidin | Swisstarget prediction |
| CA13 | Pelargonidin | Swisstarget prediction |
| AKR1A1 | Pelargonidin | Swisstarget prediction |
| SIRT1 | Pelargonidin | Swisstarget prediction |
| ESR1 | Pelargonidin | Swisstarget prediction |
| PFKFB3 | Pelargonidin | Swisstarget prediction |
| CDK5R1 CDK5 | Pelargonidin | Swisstarget prediction |
| CCNB3 CDK1 | Pelargonidin | Swisstarget prediction |
| CDK6 | Pelargonidin | Swisstarget prediction |
| CDK2 | Pelargonidin | Swisstarget prediction |
| ARG1 | Pelargonidin | Swisstarget prediction |
| PTGS1 | Pelargonidin | Swisstarget prediction |
| PDE4B | Pelargonidin | Swisstarget prediction |
| NOX4 | Kaempferol | Swisstarget prediction |
| AKR1B1 | Kaempferol | Swisstarget prediction |
| XDH | Kaempferol | Swisstarget prediction |
| TYR | Kaempferol | Swisstarget prediction |
| FLT3 | Kaempferol | Swisstarget prediction |
| CA2 | Kaempferol | Swisstarget prediction |
| ALOX5 | Kaempferol | Swisstarget prediction |
| CA7 | Kaempferol | Swisstarget prediction |
| HSD17B2 | Kaempferol | Swisstarget prediction |
| ABCC1 | Kaempferol | Swisstarget prediction |
| HSD17B1 | Kaempferol | Swisstarget prediction |
| AHR | Kaempferol | Swisstarget prediction |
| CA12 | Kaempferol | Swisstarget prediction |
| ESRRA | Kaempferol | Swisstarget prediction |
| ABCB1 | Kaempferol | Swisstarget prediction |
| CYP1B1 | Kaempferol | Swisstarget prediction |
| ABCG2 | Kaempferol | Swisstarget prediction |
| ADORA1 | Kaempferol | Swisstarget prediction |
| CA4 | Kaempferol | Swisstarget prediction |
| ACHE | Kaempferol | Swisstarget prediction |
| MAOA | Kaempferol | Swisstarget prediction |
| GLO1 | Kaempferol | Swisstarget prediction |
| SYK | Kaempferol | Swisstarget prediction |
| GSK3B | Kaempferol | Swisstarget prediction |
| MMP9 | Kaempferol | Swisstarget prediction |
| MMP2 | Kaempferol | Swisstarget prediction |
| ALOX15 | Kaempferol | Swisstarget prediction |
| ALOX12 | Kaempferol | Swisstarget prediction |
| PTPRS | Kaempferol | Swisstarget prediction |
| ADORA2A | Kaempferol | Swisstarget prediction |
| CDK5R1 CDK5 | Kaempferol | Swisstarget prediction |
| CCNB3 CDK1 | Kaempferol | Swisstarget prediction |
| ARG1 | Kaempferol | Swisstarget prediction |
| GPR35 | Kaempferol | Swisstarget prediction |
| ESR2 | Kaempferol | Swisstarget prediction |
| DAPK1 | Kaempferol | Swisstarget prediction |
| MPG | Kaempferol | Swisstarget prediction |
| SLC22A12 | Kaempferol | Swisstarget prediction |
| TTR | Kaempferol | Swisstarget prediction |
| AKR1B10 | Kaempferol | Swisstarget prediction |
| TNKS2 | Kaempferol | Swisstarget prediction |
| TNKS | Kaempferol | Swisstarget prediction |
| CDK6 | Kaempferol | Swisstarget prediction |
| CDK2 | Kaempferol | Swisstarget prediction |
| CYP19A1 | Kaempferol | Swisstarget prediction |
| CSNK2A1 | Kaempferol | Swisstarget prediction |
| EGFR | Kaempferol | Swisstarget prediction |
| AVPR2 | Kaempferol | Swisstarget prediction |
| IGF1R | Kaempferol | Swisstarget prediction |
| F2 | Kaempferol | Swisstarget prediction |
| PIM1 | Kaempferol | Swisstarget prediction |
| AURKB | Kaempferol | Swisstarget prediction |
| DRD4 | Kaempferol | Swisstarget prediction |
| MPO | Kaempferol | Swisstarget prediction |
| PIK3R1 | Kaempferol | Swisstarget prediction |
| PYGL | Kaempferol | Swisstarget prediction |
| CA1 | Kaempferol | Swisstarget prediction |
| SRC | Kaempferol | Swisstarget prediction |
| PTK2 | Kaempferol | Swisstarget prediction |
| KDR | Kaempferol | Swisstarget prediction |
| MMP13 | Kaempferol | Swisstarget prediction |
| MMP3 | Kaempferol | Swisstarget prediction |
| CA3 | Kaempferol | Swisstarget prediction |
| PLK1 | Kaempferol | Swisstarget prediction |
| CA6 | Kaempferol | Swisstarget prediction |
| CDK1 | Kaempferol | Swisstarget prediction |
| PKN1 | Kaempferol | Swisstarget prediction |
| CA14 | Kaempferol | Swisstarget prediction |
| CA9 | Kaempferol | Swisstarget prediction |
| MET | Kaempferol | Swisstarget prediction |
| NEK2 | Kaempferol | Swisstarget prediction |
| CXCR1 | Kaempferol | Swisstarget prediction |
| CAMK2B | Kaempferol | Swisstarget prediction |
| ALK | Kaempferol | Swisstarget prediction |
| AKT1 | Kaempferol | Swisstarget prediction |
| NEK6 | Kaempferol | Swisstarget prediction |
| PLA2G1B | Kaempferol | Swisstarget prediction |
| CA5A | Kaempferol | Swisstarget prediction |
| BACE1 | Kaempferol | Swisstarget prediction |
| AXL | Kaempferol | Swisstarget prediction |
| NUAK1 | Kaempferol | Swisstarget prediction |
| AKR1C2 | Kaempferol | Swisstarget prediction |
| AKR1C1 | Kaempferol | Swisstarget prediction |
| AKR1C3 | Kaempferol | Swisstarget prediction |
| AKR1C4 | Kaempferol | Swisstarget prediction |
| CA13 | Kaempferol | Swisstarget prediction |
| AKR1A1 | Kaempferol | Swisstarget prediction |
| APP | Kaempferol | Swisstarget prediction |
| PARP1 | Kaempferol | Swisstarget prediction |
| MMP12 | Kaempferol | Swisstarget prediction |
| CD38 | Kaempferol | Swisstarget prediction |
| TOP1 | Kaempferol | Swisstarget prediction |
| ESR1 | Kaempferol | Swisstarget prediction |
| PTGS2 | Kaempferol | Swisstarget prediction |
| CFTR | Kaempferol | Swisstarget prediction |
| PFKFB3 | Kaempferol | Swisstarget prediction |
| AMY1A | Kaempferol | Swisstarget prediction |
| GRK6 | Kaempferol | Swisstarget prediction |
| TERT | Kaempferol | Swisstarget prediction |
| MAPT | Kaempferol | Swisstarget prediction |
| ALOX5 | Zhepeiresinol | Swisstarget prediction |
| ERN1 | Zhepeiresinol | Swisstarget prediction |
| ELAVL1 | Zhepeiresinol | Swisstarget prediction |
| PIK3CD | Zhepeiresinol | Swisstarget prediction |
| PRKDC | Zhepeiresinol | Swisstarget prediction |
| PIK3CB | Zhepeiresinol | Swisstarget prediction |
| HSD17B3 | Zhepeiresinol | Swisstarget prediction |
| CCNE2 CDK2 CCNE1 | Zhepeiresinol | Swisstarget prediction |
| PDE10A | Zhepeiresinol | Swisstarget prediction |
| PARP1 | Zhepeiresinol | Swisstarget prediction |
| EIF2AK3 | Zhepeiresinol | Swisstarget prediction |
| PTAFR | Zhepeiresinol | Swisstarget prediction |
| PIK3CG | Zhepeiresinol | Swisstarget prediction |
| PIK3CA | Zhepeiresinol | Swisstarget prediction |
| TYMP | Zhepeiresinol | Swisstarget prediction |
| FBP1 | Zhepeiresinol | Swisstarget prediction |
| PNP | Zhepeiresinol | Swisstarget prediction |
| FFAR1 | Zhepeiresinol | Swisstarget prediction |
| GABRB3 GABRA3 GABRG2 | Zhepeiresinol | Swisstarget prediction |
| GABRB3 GABRG2 GABRA1 | Zhepeiresinol | Swisstarget prediction |
| GABRB3 GABRG2 GABRA5 | Zhepeiresinol | Swisstarget prediction |
| GABRA2 GABRB3 GABRG2 | Zhepeiresinol | Swisstarget prediction |
| GABRG2 GABRB3 GABRA6 | Zhepeiresinol | Swisstarget prediction |
| NQO2 | Zhepeiresinol | Swisstarget prediction |
| TOP2A | Zhepeiresinol | Swisstarget prediction |
| NR3C1 | Picropodophyllin | Swisstarget prediction |
| CYP2C9 | Picropodophyllin | Swisstarget prediction |
| CYP3A4 | Picropodophyllin | Swisstarget prediction |
| CYP2C19 | Picropodophyllin | Swisstarget prediction |
| SYK | Picropodophyllin | Swisstarget prediction |
| P2RX3 | Picropodophyllin | Swisstarget prediction |
| CDK5R1 CDK5 | Picropodophyllin | Swisstarget prediction |
| IMPDH2 | Picropodophyllin | Swisstarget prediction |
| JAK3 | Picropodophyllin | Swisstarget prediction |
| JAK2 | Picropodophyllin | Swisstarget prediction |
| MTOR | Picropodophyllin | Swisstarget prediction |
| PIK3CA | Picropodophyllin | Swisstarget prediction |
| JAK1 | Picropodophyllin | Swisstarget prediction |
| MERTK | Picropodophyllin | Swisstarget prediction |
| GSK3B | Picropodophyllin | Swisstarget prediction |
| GSK3A | Picropodophyllin | Swisstarget prediction |
| ABCC9 | Picropodophyllin | Swisstarget prediction |
| TYK2 | Picropodophyllin | Swisstarget prediction |
| ALPL | Picropodophyllin | Swisstarget prediction |
| MTNR1A | Picropodophyllin | Swisstarget prediction |
| MTNR1B | Picropodophyllin | Swisstarget prediction |
| EGFR | Picropodophyllin | Swisstarget prediction |
| NTRK1 | Picropodophyllin | Swisstarget prediction |
| DYRK1A | Picropodophyllin | Swisstarget prediction |
| CLK1 | Picropodophyllin | Swisstarget prediction |
| CCNB3 CDK1 CCNB1 CCNB2 | Picropodophyllin | Swisstarget prediction |
| PIK3CD | Picropodophyllin | Swisstarget prediction |
| PIK3CB | Picropodophyllin | Swisstarget prediction |
| MMP9 | Picropodophyllin | Swisstarget prediction |
| PIK3CG | Picropodophyllin | Swisstarget prediction |
| EDNRA | Picropodophyllin | Swisstarget prediction |
| ABL1 | Picropodophyllin | Swisstarget prediction |
| GRM5 | Picropodophyllin | Swisstarget prediction |
| P2RX7 | Picropodophyllin | Swisstarget prediction |
| CXCR2 | Picropodophyllin | Swisstarget prediction |
| PDE2A | Picropodophyllin | Swisstarget prediction |
| CDK2 | Picropodophyllin | Swisstarget prediction |
| CDK1 | Picropodophyllin | Swisstarget prediction |
| GPR139 | Picropodophyllin | Swisstarget prediction |
| PDE10A | Picropodophyllin | Swisstarget prediction |
| DRD1 | Picropodophyllin | Swisstarget prediction |
| FAP | Picropodophyllin | Swisstarget prediction |
| LRRK2 | Picropodophyllin | Swisstarget prediction |
| GRK7 | Picropodophyllin | Swisstarget prediction |
| STK38 | Picropodophyllin | Swisstarget prediction |
| HIPK4 | Picropodophyllin | Swisstarget prediction |
| TAOK2 | Picropodophyllin | Swisstarget prediction |
| ERN1 | Picropodophyllin | Swisstarget prediction |
| OXSR1 | Picropodophyllin | Swisstarget prediction |
| MAK | Picropodophyllin | Swisstarget prediction |
| STK39 | Picropodophyllin | Swisstarget prediction |
| CDKL5 | Picropodophyllin | Swisstarget prediction |
| CDKL3 | Picropodophyllin | Swisstarget prediction |
| PRPF4 | Picropodophyllin | Swisstarget prediction |
| PIK3C2G | Picropodophyllin | Swisstarget prediction |
| MAP3K6 | Picropodophyllin | Swisstarget prediction |
| MAP3K13 | Picropodophyllin | Swisstarget prediction |
| ICK | Picropodophyllin | Swisstarget prediction |
| MAP3K15 | Picropodophyllin | Swisstarget prediction |
| MAST1 | Picropodophyllin | Swisstarget prediction |
| SBK1 | Picropodophyllin | Swisstarget prediction |
| HUNK | Picropodophyllin | Swisstarget prediction |
| CDK13 | Picropodophyllin | Swisstarget prediction |
| CSF1R | Picropodophyllin | Swisstarget prediction |
| FLT1 | Picropodophyllin | Swisstarget prediction |
| CCND1 CDK4 | Picropodophyllin | Swisstarget prediction |
| CASK | Picropodophyllin | Swisstarget prediction |
| PRPF4B | Picropodophyllin | Swisstarget prediction |
| DSTYK | Picropodophyllin | Swisstarget prediction |
| MAP3K12 | Picropodophyllin | Swisstarget prediction |
| PDGFRB | Picropodophyllin | Swisstarget prediction |
| KIT | Picropodophyllin | Swisstarget prediction |
| IGF1R | Picropodophyllin | Swisstarget prediction |
| INSR | Picropodophyllin | Swisstarget prediction |
| IKBKB | Picropodophyllin | Swisstarget prediction |
| RET | Picropodophyllin | Swisstarget prediction |
| EPHA2 | Picropodophyllin | Swisstarget prediction |
| YES1 | Picropodophyllin | Swisstarget prediction |
| MAP2K3 | Picropodophyllin | Swisstarget prediction |
| PRKAA2 | Picropodophyllin | Swisstarget prediction |
| MAP2K6 | Picropodophyllin | Swisstarget prediction |
| AURKB | Picropodophyllin | Swisstarget prediction |
| MAPKAPK2 | Picropodophyllin | Swisstarget prediction |
| BLK | Picropodophyllin | Swisstarget prediction |
| HTR2C | Picropodophyllin | Swisstarget prediction |
| MAPK8 | Picropodophyllin | Swisstarget prediction |
| RPS6KA3 | Picropodophyllin | Swisstarget prediction |
| PHKG2 | Picropodophyllin | Swisstarget prediction |
| MYLK | Picropodophyllin | Swisstarget prediction |
| AKT2 | Picropodophyllin | Swisstarget prediction |
| DAPK3 | Picropodophyllin | Swisstarget prediction |
| CAMK1 | Picropodophyllin | Swisstarget prediction |
| CAMK4 | Picropodophyllin | Swisstarget prediction |
| CHEK2 | Picropodophyllin | Swisstarget prediction |
| PDPK1 | Picropodophyllin | Swisstarget prediction |
| RPS6KA1 | Picropodophyllin | Swisstarget prediction |
| DAPK1 | Picropodophyllin | Swisstarget prediction |
| LCK | Picropodophyllin | Swisstarget prediction |
| PRKD3 | Picropodophyllin | Swisstarget prediction |
| PRKCI | Picropodophyllin | Swisstarget prediction |
| ALOX5 | Syringaresinol | Swisstarget prediction |
| PTAFR | Syringaresinol | Swisstarget prediction |
| MAPK9 | Syringaresinol | Swisstarget prediction |
| MCL1 | Syringaresinol | Swisstarget prediction |
| SHBG | Syringaresinol | Swisstarget prediction |
| SLC5A2 | Syringaresinol | Swisstarget prediction |
| SOAT1 | Syringaresinol | Swisstarget prediction |
| HIF1A | Syringaresinol | Swisstarget prediction |
| SOAT2 | Syringaresinol | Swisstarget prediction |
| SLC6A2 | Syringaresinol | Swisstarget prediction |
| SLC6A4 | Syringaresinol | Swisstarget prediction |
| CNR2 | Syringaresinol | Swisstarget prediction |
| CYP19A1 | [(4R,9R,10S,14R,16R)-5,5,9-trimethyl-15-oxapentacyclo[11.3.1.01,10.04,9.014,16]heptadecan-14-yl]methanol | Swisstarget prediction |
| HSD11B1 | [(4R,9R,10S,14R,16R)-5,5,9-trimethyl-15-oxapentacyclo[11.3.1.01,10.04,9.014,16]heptadecan-14-yl]methanol | Swisstarget prediction |
| IDO1 | [(4R,9R,10S,14R,16R)-5,5,9-trimethyl-15-oxapentacyclo[11.3.1.01,10.04,9.014,16]heptadecan-14-yl]methanol | Swisstarget prediction |
| EPHX2 | [(4R,9R,10S,14R,16R)-5,5,9-trimethyl-15-oxapentacyclo[11.3.1.01,10.04,9.014,16]heptadecan-14-yl]methanol | Swisstarget prediction |
| PRKCB | [(4R,9R,10S,14R,16R)-5,5,9-trimethyl-15-oxapentacyclo[11.3.1.01,10.04,9.014,16]heptadecan-14-yl]methanol | Swisstarget prediction |
| KCNA3 | [(4R,9R,10S,14R,16R)-5,5,9-trimethyl-15-oxapentacyclo[11.3.1.01,10.04,9.014,16]heptadecan-14-yl]methanol | Swisstarget prediction |
| CDC25A | [(4R,9R,10S,14R,16R)-5,5,9-trimethyl-15-oxapentacyclo[11.3.1.01,10.04,9.014,16]heptadecan-14-yl]methanol | Swisstarget prediction |
| GCGR | [(4R,9R,10S,14R,16R)-5,5,9-trimethyl-15-oxapentacyclo[11.3.1.01,10.04,9.014,16]heptadecan-14-yl]methanol | Swisstarget prediction |
| IL6ST | [(4R,9R,10S,14R,16R)-5,5,9-trimethyl-15-oxapentacyclo[11.3.1.01,10.04,9.014,16]heptadecan-14-yl]methanol | Swisstarget prediction |
| TRPA1 | [(4R,9R,10S,14R,16R)-5,5,9-trimethyl-15-oxapentacyclo[11.3.1.01,10.04,9.014,16]heptadecan-14-yl]methanol | Swisstarget prediction |
| CYP17A1 | [(4R,9R,10S,14R,16R)-5,5,9-trimethyl-15-oxapentacyclo[11.3.1.01,10.04,9.014,16]heptadecan-14-yl]methanol | Swisstarget prediction |
| PSEN2 PSENEN NCSTN APH1A PSEN1 APH1B | [(4R,9R,10S,14R,16R)-5,5,9-trimethyl-15-oxapentacyclo[11.3.1.01,10.04,9.014,16]heptadecan-14-yl]methanol | Swisstarget prediction |
| AKR1C3 | [(4R,9R,10S,14R,16R)-5,5,9-trimethyl-15-oxapentacyclo[11.3.1.01,10.04,9.014,16]heptadecan-14-yl]methanol | Swisstarget prediction |
| C5AR1 | [(4R,9R,10S,14R,16R)-5,5,9-trimethyl-15-oxapentacyclo[11.3.1.01,10.04,9.014,16]heptadecan-14-yl]methanol | Swisstarget prediction |
| PER2 | [(4R,9R,10S,14R,16R)-5,5,9-trimethyl-15-oxapentacyclo[11.3.1.01,10.04,9.014,16]heptadecan-14-yl]methanol | Swisstarget prediction |
| PGR | [(4R,9R,10S,14R,16R)-5,5,9-trimethyl-15-oxapentacyclo[11.3.1.01,10.04,9.014,16]heptadecan-14-yl]methanol | Swisstarget prediction |
| G6PD | [(4R,9R,10S,14R,16R)-5,5,9-trimethyl-15-oxapentacyclo[11.3.1.01,10.04,9.014,16]heptadecan-14-yl]methanol | Swisstarget prediction |
| RASGRP1 | [(4R,9R,10S,14R,16R)-5,5,9-trimethyl-15-oxapentacyclo[11.3.1.01,10.04,9.014,16]heptadecan-14-yl]methanol | Swisstarget prediction |
| EDNRA | [(4R,9R,10S,14R,16R)-5,5,9-trimethyl-15-oxapentacyclo[11.3.1.01,10.04,9.014,16]heptadecan-14-yl]methanol | Swisstarget prediction |
| MAPK14 | [(4R,9R,10S,14R,16R)-5,5,9-trimethyl-15-oxapentacyclo[11.3.1.01,10.04,9.014,16]heptadecan-14-yl]methanol | Swisstarget prediction |
| CHRM4 | [(4R,9R,10S,14R,16R)-5,5,9-trimethyl-15-oxapentacyclo[11.3.1.01,10.04,9.014,16]heptadecan-14-yl]methanol | Swisstarget prediction |
| CHRM5 | [(4R,9R,10S,14R,16R)-5,5,9-trimethyl-15-oxapentacyclo[11.3.1.01,10.04,9.014,16]heptadecan-14-yl]methanol | Swisstarget prediction |
| CHRM2 | [(4R,9R,10S,14R,16R)-5,5,9-trimethyl-15-oxapentacyclo[11.3.1.01,10.04,9.014,16]heptadecan-14-yl]methanol | Swisstarget prediction |
| CHRM1 | [(4R,9R,10S,14R,16R)-5,5,9-trimethyl-15-oxapentacyclo[11.3.1.01,10.04,9.014,16]heptadecan-14-yl]methanol | Swisstarget prediction |
| CHRM3 | [(4R,9R,10S,14R,16R)-5,5,9-trimethyl-15-oxapentacyclo[11.3.1.01,10.04,9.014,16]heptadecan-14-yl]methanol | Swisstarget prediction |
| CYP2C9 | [(4R,9R,10S,14R,16R)-5,5,9-trimethyl-15-oxapentacyclo[11.3.1.01,10.04,9.014,16]heptadecan-14-yl]methanol | Swisstarget prediction |
| CYP2C19 | [(4R,9R,10S,14R,16R)-5,5,9-trimethyl-15-oxapentacyclo[11.3.1.01,10.04,9.014,16]heptadecan-14-yl]methanol | Swisstarget prediction |
| JAK1 | [(4R,9R,10S,14R,16R)-5,5,9-trimethyl-15-oxapentacyclo[11.3.1.01,10.04,9.014,16]heptadecan-14-yl]methanol | Swisstarget prediction |
| JAK2 | [(4R,9R,10S,14R,16R)-5,5,9-trimethyl-15-oxapentacyclo[11.3.1.01,10.04,9.014,16]heptadecan-14-yl]methanol | Swisstarget prediction |
| CYP3A4 | [(4R,9R,10S,14R,16R)-5,5,9-trimethyl-15-oxapentacyclo[11.3.1.01,10.04,9.014,16]heptadecan-14-yl]methanol | Swisstarget prediction |
| EGFR | [(4R,9R,10S,14R,16R)-5,5,9-trimethyl-15-oxapentacyclo[11.3.1.01,10.04,9.014,16]heptadecan-14-yl]methanol | Swisstarget prediction |
| KCNH2 | [(4R,9R,10S,14R,16R)-5,5,9-trimethyl-15-oxapentacyclo[11.3.1.01,10.04,9.014,16]heptadecan-14-yl]methanol | Swisstarget prediction |
| SERPINA6 | [(4R,9R,10S,14R,16R)-5,5,9-trimethyl-15-oxapentacyclo[11.3.1.01,10.04,9.014,16]heptadecan-14-yl]methanol | Swisstarget prediction |
| POLB | [(4R,9R,10S,14R,16R)-5,5,9-trimethyl-15-oxapentacyclo[11.3.1.01,10.04,9.014,16]heptadecan-14-yl]methanol | Swisstarget prediction |
| NR3C1 | [(4R,9R,10S,14R,16R)-5,5,9-trimethyl-15-oxapentacyclo[11.3.1.01,10.04,9.014,16]heptadecan-14-yl]methanol | Swisstarget prediction |
| CCNB3 CDK1 CCNB1 CCNB2 | [(4R,9R,10S,14R,16R)-5,5,9-trimethyl-15-oxapentacyclo[11.3.1.01,10.04,9.014,16]heptadecan-14-yl]methanol | Swisstarget prediction |
| DRD2 | [(4R,9R,10S,14R,16R)-5,5,9-trimethyl-15-oxapentacyclo[11.3.1.01,10.04,9.014,16]heptadecan-14-yl]methanol | Swisstarget prediction |
| CCR1 | [(4R,9R,10S,14R,16R)-5,5,9-trimethyl-15-oxapentacyclo[11.3.1.01,10.04,9.014,16]heptadecan-14-yl]methanol | Swisstarget prediction |
| GSK3B | [(4R,9R,10S,14R,16R)-5,5,9-trimethyl-15-oxapentacyclo[11.3.1.01,10.04,9.014,16]heptadecan-14-yl]methanol | Swisstarget prediction |
| HSD17B2 | [(4R,9R,10S,14R,16R)-5,5,9-trimethyl-15-oxapentacyclo[11.3.1.01,10.04,9.014,16]heptadecan-14-yl]methanol | Swisstarget prediction |
| KCNA5 | [(4R,9R,10S,14R,16R)-5,5,9-trimethyl-15-oxapentacyclo[11.3.1.01,10.04,9.014,16]heptadecan-14-yl]methanol | Swisstarget prediction |
| SLC18A3 | [(4R,9R,10S,14R,16R)-5,5,9-trimethyl-15-oxapentacyclo[11.3.1.01,10.04,9.014,16]heptadecan-14-yl]methanol | Swisstarget prediction |
| TRPV1 | [(4R,9R,10S,14R,16R)-5,5,9-trimethyl-15-oxapentacyclo[11.3.1.01,10.04,9.014,16]heptadecan-14-yl]methanol | Swisstarget prediction |
| GBA | [(4R,9R,10S,14R,16R)-5,5,9-trimethyl-15-oxapentacyclo[11.3.1.01,10.04,9.014,16]heptadecan-14-yl]methanol | Swisstarget prediction |
| LRRK2 | [(4R,9R,10S,14R,16R)-5,5,9-trimethyl-15-oxapentacyclo[11.3.1.01,10.04,9.014,16]heptadecan-14-yl]methanol | Swisstarget prediction |
| SIGMAR1 | [(4R,9R,10S,14R,16R)-5,5,9-trimethyl-15-oxapentacyclo[11.3.1.01,10.04,9.014,16]heptadecan-14-yl]methanol | Swisstarget prediction |
| NPY5R | [(4R,9R,10S,14R,16R)-5,5,9-trimethyl-15-oxapentacyclo[11.3.1.01,10.04,9.014,16]heptadecan-14-yl]methanol | Swisstarget prediction |
| JAK3 | [(4R,9R,10S,14R,16R)-5,5,9-trimethyl-15-oxapentacyclo[11.3.1.01,10.04,9.014,16]heptadecan-14-yl]methanol | Swisstarget prediction |
| TYK2 | [(4R,9R,10S,14R,16R)-5,5,9-trimethyl-15-oxapentacyclo[11.3.1.01,10.04,9.014,16]heptadecan-14-yl]methanol | Swisstarget prediction |
| MMP1 | [(4R,9R,10S,14R,16R)-5,5,9-trimethyl-15-oxapentacyclo[11.3.1.01,10.04,9.014,16]heptadecan-14-yl]methanol | Swisstarget prediction |
| PTGS1 | [(4R,9R,10S,14R,16R)-5,5,9-trimethyl-15-oxapentacyclo[11.3.1.01,10.04,9.014,16]heptadecan-14-yl]methanol | Swisstarget prediction |
| PTGS2 | [(4R,9R,10S,14R,16R)-5,5,9-trimethyl-15-oxapentacyclo[11.3.1.01,10.04,9.014,16]heptadecan-14-yl]methanol | Swisstarget prediction |
| TACR2 | [(4R,9R,10S,14R,16R)-5,5,9-trimethyl-15-oxapentacyclo[11.3.1.01,10.04,9.014,16]heptadecan-14-yl]methanol | Swisstarget prediction |
| TACR1 | [(4R,9R,10S,14R,16R)-5,5,9-trimethyl-15-oxapentacyclo[11.3.1.01,10.04,9.014,16]heptadecan-14-yl]methanol | Swisstarget prediction |
| HIF1A | [(4R,9R,10S,14R,16R)-5,5,9-trimethyl-15-oxapentacyclo[11.3.1.01,10.04,9.014,16]heptadecan-14-yl]methanol | Swisstarget prediction |
| PIM1 | [(4R,9R,10S,14R,16R)-5,5,9-trimethyl-15-oxapentacyclo[11.3.1.01,10.04,9.014,16]heptadecan-14-yl]methanol | Swisstarget prediction |
| PDE2A | [(4R,9R,10S,14R,16R)-5,5,9-trimethyl-15-oxapentacyclo[11.3.1.01,10.04,9.014,16]heptadecan-14-yl]methanol | Swisstarget prediction |
| PDE10A | [(4R,9R,10S,14R,16R)-5,5,9-trimethyl-15-oxapentacyclo[11.3.1.01,10.04,9.014,16]heptadecan-14-yl]methanol | Swisstarget prediction |
| PIM2 | [(4R,9R,10S,14R,16R)-5,5,9-trimethyl-15-oxapentacyclo[11.3.1.01,10.04,9.014,16]heptadecan-14-yl]methanol | Swisstarget prediction |
| HSD11B1 | Ent-kauran-16,17-diol | Swisstarget prediction |
| UGT2B7 | Ent-kauran-16,17-diol | Swisstarget prediction |
| AR | Ent-kauran-16,17-diol | Swisstarget prediction |
| ESR1 | Ent-kauran-16,17-diol | Swisstarget prediction |
| ESR2 | Ent-kauran-16,17-diol | Swisstarget prediction |
| SHBG | Ent-kauran-16,17-diol | Swisstarget prediction |
| POLA1 | Ent-kauran-16,17-diol | Swisstarget prediction |
| CDC25A | Ent-kauran-16,17-diol | Swisstarget prediction |
| CDC25B | Ent-kauran-16,17-diol | Swisstarget prediction |
| NR1I3 | Ent-kauran-16,17-diol | Swisstarget prediction |
| NPC1L1 | Ent-kauran-16,17-diol | Swisstarget prediction |
| GPBAR1 | Ent-kauran-16,17-diol | Swisstarget prediction |
| SHH | Ent-kauran-16,17-diol | Swisstarget prediction |
| CA2 | Ent-kauran-16,17-diol | Swisstarget prediction |
| CA1 | Ent-kauran-16,17-diol | Swisstarget prediction |
| CA4 | Ent-kauran-16,17-diol | Swisstarget prediction |
| NR1H4 | Ent-kauran-16,17-diol | Swisstarget prediction |
| NR3C2 | Ent-kauran-16,17-diol | Swisstarget prediction |
| NR3C1 | Ent-kauran-16,17-diol | Swisstarget prediction |
| PGR | Ent-kauran-16,17-diol | Swisstarget prediction |
| CYP19A1 | Ent-kauran-16,17-diol | Swisstarget prediction |
| IDO1 | Ent-kauran-16,17-diol | Swisstarget prediction |
| NR1H3 | Ent-kauran-16,17-diol | Swisstarget prediction |
| CYP17A1 | Ent-kauran-16,17-diol | Swisstarget prediction |
| JAK1 | Ent-kauran-16,17-diol | Swisstarget prediction |
| JAK2 | Ent-kauran-16,17-diol | Swisstarget prediction |
| SRD5A1 | Ent-kauran-16,17-diol | Swisstarget prediction |
| SRD5A2 | Ent-kauran-16,17-diol | Swisstarget prediction |
| TRPM8 | Ent-kauran-16,17-diol | Swisstarget prediction |
| SLC6A3 | Ent-kauran-16,17-diol | Swisstarget prediction |
| ADORA3 | Ent-kauran-16,17-diol | Swisstarget prediction |
| MAPK3 | Ent-kauran-16,17-diol | Swisstarget prediction |
| PSEN2 PSENEN NCSTN APH1A PSEN1 APH1B | Ent-kauran-16,17-diol | Swisstarget prediction |
| JAK3 | Ent-kauran-16,17-diol | Swisstarget prediction |
| TYK2 | Ent-kauran-16,17-diol | Swisstarget prediction |
| SERPINA6 | Ent-kauran-16,17-diol | Swisstarget prediction |
| KCNA3 | Ent-kauran-16,17-diol | Swisstarget prediction |
| OPRM1 | Ent-kauran-16,17-diol | Swisstarget prediction |
| OPRD1 | Ent-kauran-16,17-diol | Swisstarget prediction |
| OPRK1 | Ent-kauran-16,17-diol | Swisstarget prediction |
| EPHX2 | Ent-kauran-16,17-diol | Swisstarget prediction |
| PIM1 | Ent-kauran-16,17-diol | Swisstarget prediction |
| HMGCR | Ent-kauran-16,17-diol | Swisstarget prediction |
| PIM3 | Ent-kauran-16,17-diol | Swisstarget prediction |
| PRKCG | Ent-kauran-16,17-diol | Swisstarget prediction |
| PRKCD | Ent-kauran-16,17-diol | Swisstarget prediction |
| PRKCA | Ent-kauran-16,17-diol | Swisstarget prediction |
| PRKCB | Ent-kauran-16,17-diol | Swisstarget prediction |
| PRKCE | Ent-kauran-16,17-diol | Swisstarget prediction |
| PRKCH | Ent-kauran-16,17-diol | Swisstarget prediction |
| PRKCQ | Ent-kauran-16,17-diol | Swisstarget prediction |
| TRPV1 | Ent-kauran-16,17-diol | Swisstarget prediction |
| KCNH2 | Ent-kauran-16,17-diol | Swisstarget prediction |
| CNR2 | Ent-kauran-16,17-diol | Swisstarget prediction |
| HSD11B2 | Ent-kauran-16,17-diol | Swisstarget prediction |
| C5AR1 | Ent-kauran-16,17-diol | Swisstarget prediction |
| PRMT3 | Ent-kauran-16,17-diol | Swisstarget prediction |
| MMP3 | Ent-kauran-16,17-diol | Swisstarget prediction |
| MMP9 | Ent-kauran-16,17-diol | Swisstarget prediction |
| MMP1 | Ent-kauran-16,17-diol | Swisstarget prediction |
| RASGRP1 | Ent-kauran-16,17-diol | Swisstarget prediction |
| CA2 | Butylated hydroxytoluene | Swisstarget prediction |
| GABRA1 GABRB2 GABRG2 | Butylated hydroxytoluene | Swisstarget prediction |
| HTR2B | Butylated hydroxytoluene | Swisstarget prediction |
| GABRB3 GABRG2 GABRA1 | Butylated hydroxytoluene | Swisstarget prediction |
| PTGS1 | Butylated hydroxytoluene | Swisstarget prediction |
| SLC6A2 | Butylated hydroxytoluene | Swisstarget prediction |
| HTR2C | Butylated hydroxytoluene | Swisstarget prediction |
| GABBR2 GABBR1 | Butylated hydroxytoluene | Swisstarget prediction |
| RORC | Butylated hydroxytoluene | Swisstarget prediction |
| ESRRG | Butylated hydroxytoluene | Swisstarget prediction |
| TRPA1 | Butylated hydroxytoluene | Swisstarget prediction |
| ESR2 | Butylated hydroxytoluene | Swisstarget prediction |
| ESR1 | Butylated hydroxytoluene | Swisstarget prediction |
| HTR6 | Butylated hydroxytoluene | Swisstarget prediction |
| TYR | Butylated hydroxytoluene | Swisstarget prediction |
| NOS1 | Butylated hydroxytoluene | Swisstarget prediction |
| NOS3 | Butylated hydroxytoluene | Swisstarget prediction |

***Supplementary Material***

**Table S4 Details of Intersection Targets**

| **Target cluster of better properties compounds** | **Target cluster of disease** | **Intersection target** |
| --- | --- | --- |
| SHH | BIRC5 | SHH |
| SMO | ACTA2 | ADRB3 |
| EBP | COL1A1 | AKT1 |
| ADRA1A | ACE | ALK |
| ADRA1B | HMCES | CCND1 |
| ADRA1D | CYP3A4 | CCR3 |
| ADRA2A | PTGDR | DPP4 |
| ADRA2B | IL12RB2 | HTR5A |
| ADRA2C | S1PR5 | INSR |
| ADRB3 | GRIK3 | JAK3 |
| AKT1 | MACROH2A1 | KIT |
| AKT2 | HDAC9 | NOS1 |
| AKT3 | CCL12 | NOS3 |
| ALK | TIMP1 | PRKG1 |
| CCND1 | PRELP | HSD11B1 |
| CCNE2 | GPX1 | REN |
| CCR3 | TBX21 | GBA |
| CSF1R | MYO1E | AR |
| CTSC | SMIM14 | MME |
| CYP24A1 | PRODH | PRKCA |
| DPP4 | ST14 | TACR2 |
| DRD2 | AGTR1 | PLA2G1B |
| DRD3 | NEU1 | MDM2 |
| ESRRG | S1PR3 | PTGER4 |
| FKBP1A | CCL17 | PTGER2 |
| HRH3 | DLK1 | PTGER3 |
| HTR5A | EDN1 | GAA |
| HTR6 | ADCYAP1 | NR1H4 |
| HTR7 | CDH1 | ADRB2 |
| INSR | SLCO2A1 | CCR1 |
| JAK1 | FSHR | NR3C1 |
| JAK3 | KAT2B | CHRM3 |
| KIT | SMAD7 | ATP12A |
| MAP3K14 | TGFB1I1 | SLC6A4 |
| NOS1 | ST3GAL5 | CTSG |
| NOS3 | FGF7 | MTOR |
| PRKG1 | CCR5 | PIK3CA |
| ROCK2 | TNF | MAPK1 |
| RORC | PNP | IGF1R |
| TNK2 | NR2F2 | PRKCD |
| HSD17B3 | THBS2 | PARP1 |
| BCHE | PDLIM1 | CREBBP |
| HSD11B1 | ANKRD1 | HTR1B |
| CHRM2 | RAC2 | GANAB |
| CHRM1 | PODXL | GLA |
| REN | TAGLN2 | PLA2G2A |
| LSS | GABRB2 | HSP90AA1 |
| SLC18A3 | SFRP1 | HTR2A |
| UGCG | F2RL1 | RPS6KA3 |
| GBA | PLA2G2A | SRC |
| GBA2 | MMP2 | KDR |
| SRD5A2 | ACE2 | KCNH2 |
| AR | SOCS1 | CCR2 |
| SIGMAR1 | IFITM1 | OPRM1 |
| KISS1R | STAT6 | PPARA |
| MME | PTGER4 | NR1H3 |
| PRKCA | STAT3 | CHRNA3 |
| ITGA2B | BMP7 | JAK2 |
| DRD1 | PRKACA | PTGS1 |
| PLA2G2C | ETS1 | PTGS2 |
| PLA2G10 | AQP1 | PIK3CG |
| CNR1 | S100A11 | PRSS1 |
| TACR2 | HPGD | F2 |
| SLC1A2 | TUBB2A | NOX4 |
| PLA2G1B | ORM1 | XDH |
| MCHR1 | CLDN4 | TYR |
| MDM2 | FGF | FLT3 |
| PTGER4 | TGFBR1 | ALOX5 |
| PTGER2 | TGFB3 | ABCC1 |
| PTGER3 | CX3CL1 | ESRRA |
| DRD4 | MYC | ABCB1 |
| PRKCQ | SOX2 | CYP1B1 |
| MGAM | ICAM1 | ACHE |
| GLB1 | S100A6 | ADORA1 |
| GAA | GATA3 | CA4 |
| SI | GRIN2A | ALOX15 |
| NR1H4 | RDH9 | MAPT |
| OPRL1 | PDGFA | CYP19A1 |
| ADRB2 | EZR | EGFR |
| CHRNA4 | ANXA3 | MYLK |
| GPR88 | TGFBR2 | MPO |
| DNPEP | KIT | GSK3B |
| OPRD1 | PLA2G4A | MMP3 |
| CCR1 | S100A4 | CDK1 |
| NR3C1 | OAT | MMP9 |
| MAN2B1 | SOX9 | MMP2 |
| PDE10A | KRT19 | MET |
| APP | SMAD2 | CXCR1 |
| CHRM3 | NRG1 | AXL |
| CHRM4 | JUN | APEX1 |
| PPP5C | LGALS3 | CA13 |
| PPP1CA | TNFRSF11B | SIRT1 |
| CHRM5 | AREG | ESR1 |
| ATP12A | HGF | PFKFB3 |
| IRAK1 | ANXA2 | ARG1 |
| IRAK4 | ANXA1 | TTR |
| MAP3K7 | MMP3 | MMP12 |
| SLC6A2 | TGFB2 | CFTR |
| SLC6A4 | CALCA | TERT |
| SLC6A3 | MAPK14 | PRKDC |
| AGL | IL13 | TYMP |
| HSD17B7 | CASP8 | PNP |
| HTR3A | TNFSF11 | GABRG2 |
| PRKCB | TGFB1 | CYP2C9 |
| SCN9A | CP | CYP3A4 |
| CTSG | CCL4 | CYP2C19 |
| MTOR | CXCR4 | GSK3A |
| TTK | MIR2985 | ABCC9 |
| PIK3CA | RUNX2 | EDNRA |
| SSTR2 | PAPPA | CXCR2 |
| MAPK1 | SYPL | LRRK2 |
| CYP2J2 | TAC1 | FLT1 |
| TACR1 | TNFRSF1A | PDGFRB |
| IGF1R | FASLG | RET |
| MC4R | CEC3 | MAPK8 |
| PRKCD | ZFP131 | CHEK2 |
| MC5R | CD44 | LCK |
| MC3R | PLAU | HIF1A |
| NPY1R | COL1A2 | EPHX2 |
| XIAP | CSF2 | PSEN2 |
| EGLN1 | CXCL12 | PGR |
| PARP1 | GABRG2 | RASGRP1 |
| RPS6KB1 | IL12B | MAPK14 |
| IDH1 | CCL3 | SERPINA6 |
| CREBBP | NSMCE3 | TRPV1 |
| HTR1B | TRP53BP2 | MMP1 |
| HTR1D | CAR6 | NR1I3 |
| GANAB | CER1 | NR3C2 |
| FUCA1 | GABRA1 | MAPK3 |
| FUCA2 | CCN2 | PRMT3 |
| GANC | NFE2L2 | GABRA1 |
| GLA | SIAH1A | HTR2B |
| DNM1 | THRB | GABRB2 |
| MANBA | CXCL10 | ITGB3 |
| PLA2G2A | PTGS1 |  |
| HSP90AA1 | SIRT1 |  |
| MAOA | FRS3 |  |
| HTR2A | TRMT10B |  |
| MELK | PARD6G |  |
| RPS6KA3 | ZFP638 |  |
| SRC | KHDRBS2 |  |
| PIK3CD | MIR340-1 |  |
| KDR | MIR6215 |  |
| AURKA | PARP1 |  |
| KCNH2 | ARHGAP39 |  |
| HTR1A | CRPPA |  |
| CCR2 | NOS2 |  |
| OPRM1 | ZSCAN21 |  |
| NPC1L1 | ADGRB3 |  |
| OPRK1 | EGF |  |
| PPARA | MBL1 |  |
| GPR119 | CCL5 |  |
| CHEK1 | NAP1L2 |  |
| CTSS | CELA2A |  |
| NR1H3 | LSM1 |  |
| PTPN1 | MTX2 |  |
| CCNE1 | SLC4A3 |  |
| DGAT1 | UNCX |  |
| CHRNA3 | CEBPB |  |
| JAK2 | RNASEH1 |  |
| PTGS1 | RPE |  |
| PTGS2 | SCAF1 |  |
| CA2 | THOC6 |  |
| AKR1B1 | KRT2 |  |
| PIK3CG | CASP9 |  |
| PRSS1 | MEX3D |  |
| NCOA2 | OLIG3 |  |
| F2 | RANBP6 |  |
| GLO1 | UBL5 |  |
| NOX4 | AGAP3 |  |
| XDH | IPP |  |
| TYR | MIR3573 |  |
| FLT3 | MIR6328 |  |
| ALOX5 | NEUROG2 |  |
| CA7 | NT5DC1 |  |
| HSD17B2 | RGD1562690 |  |
| ABCC1 | SERPINE1 |  |
| HSD17B1 | RDH14 |  |
| AHR | NGLY1 |  |
| CA12 | TCF15 |  |
| ESRRA | TUT4 |  |
| ABCB1 | AKAP10 |  |
| CYP1B1 | CASKIN1 |  |
| ABCG2 | IL26 |  |
| CD38 | LEP |  |
| ACHE | SNX2 |  |
| ADORA1 | EIF1A |  |
| ADORA2A | FBXL3 |  |
| ADORA3 | HOXB7 |  |
| CA4 | RBP2 |  |
| GPR35 | YME1L1 |  |
| PTPRS | ZFP503 |  |
| ESR2 | ARMC8 |  |
| DAPK1 | B4GALT7 |  |
| MPG | CMPK1 |  |
| SLC22A12 | KCTD3 |  |
| ALOX15 | PLCL1 |  |
| SHBG | VARS2 |  |
| MAPT | AKT1 |  |
| KDM4E | MESP1 |  |
| AVPR2 | RBM34 |  |
| TOP2A | CNOT7 |  |
| CYP19A1 | EGFR |  |
| EGFR | TCEAL1 |  |
| PIM1 | ATPAF1 |  |
| AURKB | CHCR1 |  |
| MYLK | CHCR2 |  |
| MPO | CYP2C |  |
| PIK3R1 | MRPS18C |  |
| PYGL | PAFAH1B2 |  |
| SYK | RNF115 |  |
| CA1 | UBR1 |  |
| GSK3B | AMN1 |  |
| PTK2 | INPP5F |  |
| MMP13 | RIOK2 |  |
| MMP3 | SART3 |  |
| CA3 | ATF3 |  |
| PLK1 | HOOK3 |  |
| CA6 | KLHL7 |  |
| CDK1 | LDB2 |  |
| MMP9 | MTF2 |  |
| MMP2 | POMC |  |
| PKN1 | SULT1D1 |  |
| CA14 | ACT1 |  |
| CA9 | ARFIP1 |  |
| CSNK2A1 | CBLL1 |  |
| ALOX12 | FNDC3A |  |
| MET | GNL2 |  |
| NEK2 | HOXA2 |  |
| CXCR1 | MIR347 |  |
| CAMK2B | MIR3584 |  |
| NEK6 | MIR378 |  |
| CA5A | MIR466B-1 |  |
| BACE1 | NCAN |  |
| AXL | NELFA |  |
| APEX1 | PDGFAA |  |
| NUAK1 | RSRC1 |  |
| AKR1C2 | CYP2C37 |  |
| AKR1C1 | EBPL |  |
| AKR1C3 | GNPDA2 |  |
| AKR1C4 | KLHL2 |  |
| CA13 | KRIT1 |  |
| AKR1A1 | PPIL4 |  |
| SIRT1 | SMARCA1 |  |
| ESR1 | SMC5 |  |
| PFKFB3 | CDX2 |  |
| CDK5R1 | CLCN6 |  |
| CCNB3 | HELZ |  |
| CDK6 | SENP7 |  |
| CDK2 | STAR |  |
| ARG1 | SH3YL1 |  |
| PDE4B | DMTN |  |
| TTR | DAP3 |  |
| AKR1B10 | PHF14 |  |
| TNKS2 | UBE2D2 |  |
| TNKS | WNT3 |  |
| MMP12 | MAPRE3 |  |
| TOP1 | PARG |  |
| CFTR | SCN3A |  |
| AMY1A | SPP1 |  |
| GRK6 | TERF1 |  |
| TERT | AAAS |  |
| ERN1 | FMO4 |  |
| ELAVL1 | G6PDX |  |
| PRKDC | MIR1949 |  |
| PIK3CB | MIR3085 |  |
| EIF2AK3 | MIR352 |  |
| PTAFR | PTBP2 |  |
| TYMP | RBM12 |  |
| FBP1 | SCARNA15 |  |
| PNP | TAF9B |  |
| FFAR1 | WNT1 |  |
| GABRB3 | CAT |  |
| GABRA2 | FGF5 |  |
| GABRG2 | IL2 |  |
| NQO2 | POU3F1 |  |
| CYP2C9 | CPSF6 |  |
| CYP3A4 | CYP3A13 |  |
| CYP2C19 | IGHE |  |
| P2RX3 | CAPN5 |  |
| IMPDH2 | DAPK2 |  |
| MERTK | ITGB3BP |  |
| GSK3A | MAP3K4 |  |
| ABCC9 | PRMT3 |  |
| TYK2 | RAI1 |  |
| ALPL | SMARCA5 |  |
| MTNR1A | HOXB9 |  |
| MTNR1B | MGAT2 |  |
| NTRK1 | SCN1A |  |
| DYRK1A | SIPA1L1 |  |
| CLK1 | EMX2 |  |
| EDNRA | FMOD |  |
| ABL1 | GSK3B |  |
| GRM5 | HEXIM1 |  |
| P2RX7 | KCND2 |  |
| CXCR2 | SCN1B |  |
| PDE2A | XRCC4 |  |
| GPR139 | CEP57 |  |
| FAP | CUL4A |  |
| LRRK2 | IL6RA |  |
| GRK7 | LTBP4 |  |
| STK38 | RPL28 |  |
| HIPK4 | SLC22A22 |  |
| TAOK2 | FGF12 |  |
| OXSR1 | FOXC2 |  |
| MAK | HDAC7 |  |
| STK39 | LAP3 |  |
| CDKL5 | LRBA |  |
| CDKL3 | LYPLA1 |  |
| PRPF4 | MIR181A-1 |  |
| PIK3C2G | MIR290 |  |
| MAP3K6 | PIP4K2A |  |
| MAP3K13 | CCNC |  |
| ICK | ERVH-1 |  |
| MAP3K15 | EYA1 |  |
| MAST1 | FS(1)H |  |
| SBK1 | MAPK12 |  |
| HUNK | NUP153 |  |
| CDK13 | RELA |  |
| FLT1 | SMC3 |  |
| CASK | TLR9 |  |
| PRPF4B | EOMES |  |
| DSTYK | LHR |  |
| MAP3K12 | PNN |  |
| PDGFRB | PRSS12 |  |
| IKBKB | FGF8 |  |
| RET | FLRT2 |  |
| EPHA2 | IFNGR2 |  |
| YES1 | MEOX1 |  |
| MAP2K3 | PAX3 |  |
| PRKAA2 | PTPN3 |  |
| MAP2K6 | ZAP70 |  |
| MAPKAPK2 | GPM6A |  |
| BLK | HMOX1 |  |
| HTR2C | KYAT1 |  |
| MAPK8 | PCYOX1 |  |
| PHKG2 | SP3 |  |
| DAPK3 | ST13 |  |
| CAMK1 | H2AX |  |
| CAMK4 | IL1A |  |
| CHEK2 | MIR3473 |  |
| PDPK1 | PLXNB2 |  |
| RPS6KA1 | SCN2A |  |
| LCK | SNORA58 |  |
| PRKD3 | ABHD5 |  |
| PRKCI | IL4 |  |
| MAPK9 | LAMA2 |  |
| MCL1 | ADAMTS9 |  |
| SLC5A2 | BMP1 |  |
| SOAT1 | C2 |  |
| HIF1A | CYP2B9 |  |
| SOAT2 | ITGA8 |  |
| CNR2 | PBX1 |  |
| IDO1 | PSMC6 |  |
| EPHX2 | HIF1A |  |
| KCNA3 | DHX9 |  |
| CDC25A | PAFAH1B1 |  |
| GCGR | PSIP1 |  |
| IL6ST | ADAM8 |  |
| TRPA1 | MIR101-1 |  |
| CYP17A1 | MIR6216 |  |
| PSEN2 | NPAS2 |  |
| C5AR1 | RPS3A1 |  |
| PER2 | VEGFB |  |
| PGR | CALM2 |  |
| G6PD | ENPEP |  |
| RASGRP1 | FBXO30 |  |
| MAPK14 | HLA-B |  |
| SERPINA6 | CYP2A5 |  |
| POLB | IHH |  |
| KCNA5 | PSMD12 |  |
| TRPV1 | RAPGEF4 |  |
| NPY5R | RARG |  |
| MMP1 | STAT4 |  |
| PIM2 | FMR1 |  |
| UGT2B7 | IRF9 |  |
| POLA1 | PLEC |  |
| CDC25B | SMC2 |  |
| NR1I3 | ABLIM1 |  |
| GPBAR1 | FASL |  |
| NR3C2 | PGRMC1 |  |
| SRD5A1 | RYR2 |  |
| TRPM8 | ADRB3 |  |
| MAPK3 | CACYBP |  |
| HMGCR | CASP10 |  |
| PIM3 | MDK |  |
| PRKCG | MIR199A2 |  |
| PRKCE | MIR511 |  |
| PRKCH | NR2F1 |  |
| HSD11B2 | SYN1 |  |
| PRMT3 | COL6A2 |  |
| GABRA1 | MTHFD1L |  |
| HTR2B | RPS6KA3 |  |
| GABBR2 | FGF9 |  |
| CDK4 | FOXP3 |  |
| CDK5 | PTH |  |
| CHRNB2 | ALDH1A7 |  |
| GABBR1 | CD47 |  |
| GABRA3 | COL6A3 |  |
| GABRB2 | IFNGR1 |  |
| ITGB3 | NTF3 |  |
| PSENEN | RBM3 |  |
| CCNB1 | CYP3A7 |  |
| GABRA5 | CYP4A10 |  |
| GABRA6 | ERLEC1P1 |  |
| NCSTN | HSPA1L |  |
| APH1A | LINC00458 |  |
| CCNB2 | LINC00540 |  |
| PSEN1 | NRCAM |  |
| APH1B | TRADD |  |
|  | VCX |  |
|  | CYP4A1 |  |
|  | NCL |  |
|  | NFKB1 |  |
|  | CKM |  |
|  | COL11A1 |  |
|  | CYP2C29 |  |
|  | HNRNPA2B1 |  |
|  | PTCH1 |  |
|  | RAD50 |  |
|  | SERPINC1 |  |
|  | EGR3 |  |
|  | GSTK1 |  |
|  | RND1 |  |
|  | SHH |  |
|  | CCDC144NL |  |
|  | CYP4A14 |  |
|  | HIST1H2BG |  |
|  | LCAT |  |
|  | RPSA |  |
|  | ITGA2 |  |
|  | NOX1 |  |
|  | PAX6 |  |
|  | PLTP |  |
|  | COL6A1 |  |
|  | KLF2 |  |
|  | LINC00545 |  |
|  | MAILR |  |
|  | MAP1B |  |
|  | SLC22A7 |  |
|  | ALDH1A2 |  |
|  | F10 |  |
|  | FGF20 |  |
|  | MIR1249 |  |
|  | MIR133A1 |  |
|  | MPO |  |
|  | UGDH |  |
|  | GUSB |  |
|  | PRKDC |  |
|  | TKT |  |
|  | VCAN |  |
|  | BAX |  |
|  | IL23A |  |
|  | NES |  |
|  | S100A10 |  |
|  | UGT2B1 |  |
|  | COL18A1 |  |
|  | MIR193 |  |
|  | MIR365B |  |
|  | SDC4 |  |
|  | UNG |  |
|  | ANXA4 |  |
|  | SC5D |  |
|  | WNT5A |  |
|  | AKR7A3 |  |
|  | H2BW4P |  |
|  | S100A11P1 |  |
|  | STIMATE-MUSTN1 |  |
|  | GSTO1 |  |
|  | SYP |  |
|  | CYP7B1 |  |
|  | DNAJA1 |  |
|  | HSPE1 |  |
|  | MIR24-2 |  |
|  | MIR33 |  |
|  | UQCRHL |  |
|  | ABCG1 |  |
|  | APOA4 |  |
|  | COL4A1 |  |
|  | LPIN1 |  |
|  | FMO1 |  |
|  | NCOA1 |  |
|  | G6PC1 |  |
|  | MIR301A |  |
|  | MIR450A1 |  |
|  | C8ORF34-AS1 |  |
|  | LINC00698 |  |
|  | SOX21-AS1 |  |
|  | SULT2A1 |  |
|  | NR1H3 |  |
|  | ESR1 |  |
|  | SFTPA2 |  |
|  | MIR26A |  |
|  | MIR326 |  |
|  | MIR872 |  |
|  | CYP2B2 |  |
|  | GLRA3 |  |
|  | ABCB1B |  |
|  | BMP4 |  |
|  | FGFR2 |  |
|  | FAM226B |  |
|  | GJE1 |  |
|  | LINC00491 |  |
|  | MIR101B |  |
|  | MIR672 |  |
|  | RARB |  |
|  | VCX3A |  |
|  | ANXA5 |  |
|  | CRH |  |
|  | CTSL |  |
|  | CYP3A2 |  |
|  | GRIN1 |  |
|  | OPRM1 |  |
|  | TRPC7 |  |
|  | TGFA |  |
|  | IGF2 |  |
|  | INSR |  |
|  | MIR30C2 |  |
|  | MIR500 |  |
|  | MIR652 |  |
|  | MIR674 |  |
|  | MIR702 |  |
|  | NOX4 |  |
|  | RARA |  |
|  | BCL2 |  |
|  | CCNG1 |  |
|  | CYP1B1 |  |
|  | IL17F |  |
|  | PDGFB |  |
|  | AFP |  |
|  | LINC01405 |  |
|  | POU5F1P3 |  |
|  | POU5F1P4 |  |
|  | S100A8 |  |
|  | S100A9 |  |
|  | RHOA |  |
|  | CYP3A11 |  |
|  | ITGB1 |  |
|  | UGT1A6 |  |
|  | CYP2B10 |  |
|  | EPHX2 |  |
|  | GPX2 |  |
|  | MIR211 |  |
|  | C12ORF56 |  |
|  | CHEK2 |  |
|  | E2F1 |  |
|  | ERVH48-1 |  |
|  | GSEC |  |
|  | GSTM3 |  |
|  | IL17A |  |
|  | MAPK3 |  |
|  | NIFK-AS1 |  |
|  | ZFHX4-AS1 |  |
|  | FCER2 |  |
|  | GLUL |  |
|  | MAPK1 |  |
|  | MGMT |  |
|  | MIR338 |  |
|  | PLAUR |  |
|  | SELE |  |
|  | TMEM250 |  |
|  | CYP3A5 |  |
|  | IL10 |  |
|  | ABCB1A |  |
|  | ME1 |  |
|  | NLRP3 |  |
|  | TLR2 |  |
|  | C2ORF27A |  |
|  | CYP3A23-3A1 |  |
|  | ERVMER61-1 |  |
|  | F2 |  |
|  | HNRNPCL1 |  |
|  | KIF28P |  |
|  | ZNF503-AS2 |  |
|  | ZNF785 |  |
|  | CTSB |  |
|  | MIR151 |  |
|  | MIR181D |  |
|  | MIR322 |  |
|  | SCARB1 |  |
|  | CDH2 |  |
|  | ATM |  |
|  | GSTM2 |  |
|  | HBB-B2 |  |
|  | KDR |  |
|  | MIR296 |  |
|  | MIR361 |  |
|  | CNC |  |
|  | KLF3-AS1 |  |
|  | TSPOAP1-AS1 |  |
|  | XCL2 |  |
|  | ZSCAN16-AS1 |  |
|  | HMGB1 |  |
|  | MIR28 |  |
|  | CCL27 |  |
|  | C14ORF39 |  |
|  | DPPA5 |  |
|  | HSPD1 |  |
|  | LINC00515 |  |
|  | LINC01003 |  |
|  | LINCR |  |
|  | MIR124-2HG |  |
|  | MIR148B |  |
|  | MIR188 |  |
|  | MIR345 |  |
|  | PABPC5 |  |
|  | POU5F1B |  |
|  | APOA1 |  |
|  | CYP7A1 |  |
|  | HSPA8 |  |
|  | MMP9 |  |
|  | SULT1A1 |  |
|  | ABCA1 |  |
|  | CYP2B1 |  |
|  | MMP1 |  |
|  | PRL |  |
|  | CCDC140 |  |
|  | GSTA2 |  |
|  | HTR1F |  |
|  | LYZL6 |  |
|  | MIR32 |  |
|  | MIR328 |  |
|  | MIR409 |  |
|  | MIR542 |  |
|  | MUC3A |  |
|  | PON1 |  |
|  | PPP1R9B |  |
|  | MIR133B |  |
|  | MIR30B |  |
|  | MIR331 |  |
|  | NFKBIA |  |
|  | OVAL |  |
|  | PRL2C2 |  |
|  | APOE |  |
|  | HSPB1 |  |
|  | BEX5 |  |
|  | GSTA1 |  |
|  | KAL1 |  |
|  | MIR195 |  |
|  | MIR374B |  |
|  | MIR449A |  |
|  | MIR497 |  |
|  | RMST |  |
|  | MIR330 |  |
|  | MIR451A |  |
|  | CYP2C19 |  |
|  | IL12RB1 |  |
|  | KIAA1755 |  |
|  | POR |  |
|  | VENTX |  |
|  | MDM2 |  |
|  | MIR101A |  |
|  | MIR1224 |  |
|  | MIR362 |  |
|  | TNFSF10 |  |
|  | CYP2D6 |  |
|  | HCRT |  |
|  | HTR5B |  |
|  | MIR320A |  |
|  | MIR342 |  |
|  | MIR483 |  |
|  | PLA2G1B |  |
|  | ABCC3 |  |
|  | ALDH1A1 |  |
|  | EPHX1 |  |
|  | RNF175 |  |
|  | UGT1A1 |  |
|  | CYP4A11 |  |
|  | IL18 |  |
|  | MIR181C |  |
|  | MIR425 |  |
|  | VEGFA |  |
|  | ATP5O |  |
|  | MIR107 |  |
|  | MIR324 |  |
|  | MIR532 |  |
|  | MKI67 |  |
|  | SOD2 |  |
|  | ABCC1 |  |
|  | CYP2C9 |  |
|  | OVOS2 |  |
|  | PTGES2 |  |
|  | CD33 |  |
|  | CYP2B6 |  |
|  | HTR5A |  |
|  | IL17RA |  |
|  | MIR100 |  |
|  | MIR16 |  |
|  | MIR26B |  |
|  | MIR423 |  |
|  | PLA2G5 |  |
|  | TXNRD1 |  |
|  | APELA |  |
|  | C3ORF33 |  |
|  | C8ORF34 |  |
|  | CBX8 |  |
|  | DPY19L2P2 |  |
|  | GARIN1B |  |
|  | HLA-DPB2 |  |
|  | LINC00472 |  |
|  | MIR152 |  |
|  | MIR185 |  |
|  | MIR186 |  |
|  | MIRLET7I |  |
|  | TENT4B |  |
|  | CXCL2 |  |
|  | FGF23 |  |
|  | FOS |  |
|  | IFNG |  |
|  | SEC14L3 |  |
|  | ANXA2P3 |  |
|  | CYP19A1 |  |
|  | ESRG |  |
|  | FRRS1L |  |
|  | MIR223 |  |
|  | MIR31 |  |
|  | MIR429 |  |
|  | MIR494 |  |
|  | MIR503 |  |
|  | MIRLET7D |  |
|  | MIRLET7E |  |
|  | NMBR |  |
|  | RTP1 |  |
|  | SLC25A53 |  |
|  | IL22 |  |
|  | FN1 |  |
|  | GSTM1 |  |
|  | HRH2 |  |
|  | LHFP |  |
|  | MIR106B |  |
|  | MIR18A |  |
|  | MIR23B |  |
|  | MIR99B |  |
|  | TLR4 |  |
|  | ATF1 |  |
|  | C11ORF52 |  |
|  | CALML3 |  |
|  | COL10A1 |  |
|  | GRIN2D |  |
|  | INS |  |
|  | MIR126 |  |
|  | MIR203 |  |
|  | MIR212 |  |
|  | CDK1 |  |
|  | GADD45A |  |
|  | GCLM |  |
|  | IL2RG |  |
|  | KCNH2 |  |
|  | MIR10B |  |
|  | MIR17 |  |
|  | MIR20B |  |
|  | MIR25 |  |
|  | PLD2 |  |
|  | EGFL8 |  |
|  | MIR204 |  |
|  | MIR30D |  |
|  | MIR375 |  |
|  | NR3C1 |  |
|  | PTGFR |  |
|  | SOX2-OT |  |
|  | UGT2B28 |  |
|  | VIM |  |
|  | ZYG11A |  |
|  | H1F4 |  |
|  | MIR140 |  |
|  | MIRLET7B |  |
|  | AFDN-DT |  |
|  | GABRR1 |  |
|  | HMGCLL1 |  |
|  | KIAA1549 |  |
|  | MIR34B |  |
|  | MIR98 |  |
|  | SHISA6 |  |
|  | EGR1 |  |
|  | IGF1 |  |
|  | MIR130A |  |
|  | PSEN2 |  |
|  | PTGER1 |  |
|  | ABCC2 |  |
|  | BEST4 |  |
|  | C16ORF87 |  |
|  | C1ORF53 |  |
|  | CCR6 |  |
|  | LCTL |  |
|  | MAP1LC3C |  |
|  | MIR142 |  |
|  | MIR143 |  |
|  | MIR144 |  |
|  | MIR183 |  |
|  | MIR20A |  |
|  | MIR23A |  |
|  | MIR96 |  |
|  | TKTL1 |  |
|  | TMPRSS11E |  |
|  | WNT8A |  |
|  | ZNF519 |  |
|  | MIR146B |  |
|  | MIR15B |  |
|  | MIR214 |  |
|  | MIR29A |  |
|  | MIR30E |  |
|  | MIR34C |  |
|  | NR1I3 |  |
|  | ALKAL2 |  |
|  | ANXA2P2 |  |
|  | FAM200B |  |
|  | JAK3 |  |
|  | L1TD1 |  |
|  | MIR10A |  |
|  | TVP23A |  |
|  | UTF1 |  |
|  | ZNF436-AS1 |  |
|  | ZNF600 |  |
|  | CFAP95 |  |
|  | FAM162B |  |
|  | FOXH1 |  |
|  | GRPR |  |
|  | HCAR2 |  |
|  | HLA-DRB5 |  |
|  | IPW |  |
|  | KCNA7 |  |
|  | LEMD1 |  |
|  | MIR27A |  |
|  | NME8 |  |
|  | PIFO |  |
|  | SMN2 |  |
|  | THAP9 |  |
|  | TMEM87A |  |
|  | ZFY |  |
|  | ZNF483 |  |
|  | RAB1B |  |
|  | TACR2 |  |
|  | C19ORF33 |  |
|  | CCL2 |  |
|  | CFAP91 |  |
|  | MIR99A |  |
|  | PLAAT5 |  |
|  | SCUBE3 |  |
|  | VGF |  |
|  | ATP5ME |  |
|  | GSK3A |  |
|  | MEPCE |  |
|  | MIR182 |  |
|  | C2ORF68 |  |
|  | CCDC177 |  |
|  | MANEAL |  |
|  | MIR150 |  |
|  | OSTF1 |  |
|  | ZFP2 |  |
|  | MIR93 |  |
|  | MNX1 |  |
|  | NHSL2 |  |
|  | RASSF10 |  |
|  | SLC35D3 |  |
|  | TRPC2 |  |
|  | TRPV4 |  |
|  | TTC29 |  |
|  | ZNF287 |  |
|  | ZNF385D |  |
|  | ZNF804A |  |
|  | EPX |  |
|  | GAST |  |
|  | MIR200B |  |
|  | MIR27B |  |
|  | NQO1 |  |
|  | ZFX |  |
|  | BNIPL |  |
|  | DACH2 |  |
|  | KIAA0040 |  |
|  | MIAT |  |
|  | MUC15 |  |
|  | RPS6KA6 |  |
|  | CCKAR |  |
|  | MIR141 |  |
|  | MIR200A |  |
|  | MIR221 |  |
|  | MYL12B |  |
|  | AMER2 |  |
|  | ANOS1 |  |
|  | BLMH |  |
|  | DCAF12L2 |  |
|  | LRRC55 |  |
|  | MIR145 |  |
|  | MIR19A |  |
|  | PRDM14 |  |
|  | PTCHD1 |  |
|  | C4ORF19 |  |
|  | CCL22 |  |
|  | GHRL |  |
|  | LINGO2 |  |
|  | MIR125A |  |
|  | MIR200C |  |
|  | OSMR |  |
|  | PABPC4L |  |
|  | PCDHB14 |  |
|  | PTGER3 |  |
|  | PYM1 |  |
|  | SP140L |  |
|  | SPINK2 |  |
|  | SV2B |  |
|  | XKR4 |  |
|  | ZNF280C |  |
|  | ALX1 |  |
|  | BCL2L1 |  |
|  | COMP |  |
|  | CXCL8 |  |
|  | FBXL13 |  |
|  | GPR173 |  |
|  | LIPH |  |
|  | MIR29C |  |
|  | NWD2 |  |
|  | PTGER2 |  |
|  | SCGN |  |
|  | UGT3A1 |  |
|  | CHIL3 |  |
|  | HCLS1 |  |
|  | LHX1 |  |
|  | MIR210 |  |
|  | MIR30A |  |
|  | NIT2 |  |
|  | SFTPA1 |  |
|  | SLC12A1 |  |
|  | SSTR1 |  |
|  | TRPM2 |  |
|  | ABCB1 |  |
|  | ACHE |  |
|  | ADAMTS19 |  |
|  | COA1 |  |
|  | CYP2E1 |  |
|  | DIPK1C |  |
|  | EMILIN3 |  |
|  | FAXC |  |
|  | FUT9 |  |
|  | KCNH5 |  |
|  | KLHL42 |  |
|  | KLRG2 |  |
|  | NPFFR2 |  |
|  | NR5A1 |  |
|  | SP8 |  |
|  | TMEM178A |  |
|  | VGLL1 |  |
|  | ZFP14 |  |
|  | CGA |  |
|  | CYREN |  |
|  | DDIT3 |  |
|  | HMX2 |  |
|  | KREMEN1 |  |
|  | LIM2 |  |
|  | LRRTM1 |  |
|  | MIR222 |  |
|  | NALF1 |  |
|  | NR1I2 |  |
|  | POU3F3 |  |
|  | ZNF436 |  |
|  | BSCL2 |  |
|  | EMCN |  |
|  | GANAB |  |
|  | RETN |  |
|  | SLC9A3R2 |  |
|  | WT1 |  |
|  | ALOX15 |  |
|  | AP5S1 |  |
|  | DLX6 |  |
|  | ESRRA |  |
|  | IQCA1 |  |
|  | KCNV1 |  |
|  | NKAIN3 |  |
|  | NOL4 |  |
|  | PARK7 |  |
|  | PSMD13 |  |
|  | RALB |  |
|  | SIX6 |  |
|  | TGFBRAP1 |  |
|  | C3ORF52 |  |
|  | C6ORF141 |  |
|  | CCR7 |  |
|  | CLCN5 |  |
|  | DMRTA2 |  |
|  | FAM124A |  |
|  | FAM163A |  |
|  | PPP1R3D |  |
|  | RPRML |  |
|  | SCGB3A2 |  |
|  | TPPP3 |  |
|  | XKRX |  |
|  | ATPIF1 |  |
|  | CBR3 |  |
|  | IGSF21 |  |
|  | MIR146A |  |
|  | MUC1 |  |
|  | RAX |  |
|  | RETNLA |  |
|  | TMEM18 |  |
|  | VASP |  |
|  | ZNF521 |  |
|  | ADAMTS17 |  |
|  | BCL6B |  |
|  | CA13 |  |
|  | DLL4 |  |
|  | DUSP23 |  |
|  | ALB |  |
|  | UGT1A7 |  |
|  | UGT1A8 |  |
|  | UGT1A10 |  |
|  | VEGFR1 |  |
|  | VEGFR2 |  |
|  | VEGFR3 |  |
|  | PDGFRA |  |
|  | PDGFRB |  |
|  | FGFR1 |  |
|  | FGFR3 |  |
|  | FLT3 |  |
|  | LCK |  |
|  | LYN |  |
|  | SRC |  |
|  | SLC22A1 |  |
|  | SERPINA6 |  |
|  | SLCO1A2 |  |
|  | CYP2A6 |  |
|  | CYP2C8 |  |
|  | HSD11B1 |  |
|  | TERT |  |
|  | CFTR |  |
|  | RTEL1 |  |
|  | RTEL1-TNFRSF6B |  |
|  | SFTPC |  |
|  | BMPR2 |  |
|  | ABCA3 |  |
|  | PARN |  |
|  | LOC110806263 |  |
|  | MUC5B |  |
|  | TERC |  |
|  | SFTPB |  |
|  | DSP |  |
|  | FAM13A |  |
|  | HLA-DRB1 |  |
|  | SERPINA1 |  |
|  | ENG |  |
|  | ACVRL1 |  |
|  | KCNK3 |  |
|  | STN1 |  |
|  | CAV1 |  |
|  | ATP11A |  |
|  | DPP9 |  |
|  | CACNA1H |  |
|  | IL6 |  |
|  | FOXF1 |  |
|  | EIF2AK4 |  |
|  | FAM111B |  |
|  | SFTPD |  |
|  | LOC111674472 |  |
|  | CFTR-AS1 |  |
|  | MIF |  |
|  | LOC102723566 |  |
|  | LOC111674475 |  |
|  | HFE |  |
|  | EDNRA |  |
|  | NKX2-5 |  |
|  | IL1B |  |
|  | TBX4 |  |
|  | CSF2RA |  |
|  | ELN |  |
|  | LOC111674477 |  |
|  | TINF2 |  |
|  | RPL5 |  |
|  | NKX2-1 |  |
|  | SLC11A1 |  |
|  | SMAD9 |  |
|  | LOC113664106 |  |
|  | ZCCHC8 |  |
|  | IL1RN |  |
|  | PMS2 |  |
|  | FCGR2A |  |
|  | SMAD4 |  |
|  | ELANE |  |
|  | SLC20A2 |  |
|  | CRP |  |
|  | DIPK1A |  |
|  | TUBB3 |  |
|  | ACTC1 |  |
|  | SCN5A |  |
|  | NHP2 |  |
|  | HPS1 |  |
|  | LOC110806306 |  |
|  | JAK2 |  |
|  | NF1 |  |
|  | TMEM67 |  |
|  | SCNN1A |  |
|  | TP53 |  |
|  | MIR21 |  |
|  | GATA4 |  |
|  | HPS4 |  |
|  | SLC34A2 |  |
|  | BMP6 |  |
|  | AGT |  |
|  | CTNNB1 |  |
|  | INPP5E |  |
|  | FGF10 |  |
|  | GATA6 |  |
|  | IL2RA |  |
|  | SMAD3 |  |
|  | IL5 |  |
|  | HPS5 |  |
|  | KCNN4 |  |
|  | REN |  |
|  | PTPN11 |  |
|  | SLC9A3 |  |
|  | HPS3 |  |
|  | NOS3 |  |
|  | HPS6 |  |
|  | SARS2 |  |
|  | NOP10 |  |
|  | CEACAM3 |  |
|  | FLNA |  |
|  | HLA-DQB1 |  |
|  | FBN1 |  |
|  | COPD |  |
|  | NOTCH1 |  |
|  | STX1A |  |
|  | TOLLIP |  |
|  | RPGRIP1L |  |
|  | SLC26A9 |  |
|  | CLCN2 |  |
|  | CSF2RB |  |
|  | SLC6A14 |  |
|  | TNNT2 |  |
|  | LMNA |  |
|  | DKC1 |  |
|  | NPPB |  |
|  | SLC2A1 |  |
|  | CCL18 |  |
|  | FGF2 |  |
|  | MPL |  |
|  | CASR |  |
|  | TNNI3 |  |
|  | ADRB2 |  |
|  | PTPN22 |  |
|  | EP300 |  |
|  | TET2 |  |
|  | MUC5AC |  |
|  | CXCR3 |  |
|  | DTNBP1 |  |
|  | SCGB1A1 |  |
|  | PRTN3 |  |
|  | PKHD1 |  |
|  | KRT18 |  |
|  | FENDRR |  |
|  | THBD |  |
|  | MYH7 |  |
|  | CD36 |  |
|  | SCNN1G |  |
|  | SCNN1B |  |
|  | MBL2 |  |
|  | CALR |  |
|  | JAG1 |  |
|  | STAT1 |  |
|  | TBX1 |  |
|  | PKD1 |  |
|  | ELMOD2 |  |
|  | CCL11 |  |
|  | RYR1 |  |
|  | DYNC2H1 |  |
|  | MMP7 |  |
|  | DES |  |
|  | ALOX5 |  |
|  | CTLA4 |  |
|  | CD4 |  |
|  | IL1R1 |  |
|  | HLA-DPB1 |  |
|  | HLA-DQA1 |  |
|  | KRAS |  |
|  | MMP12 |  |
|  | MYH6 |  |
|  | MTHFR |  |
|  | CHD7 |  |
|  | TTN |  |
|  | PTGS2 |  |
|  | WRAP53 |  |
|  | IRF5 |  |
|  | F5 |  |
|  | THPO |  |
|  | MT-CO1 |  |
|  | SHOX |  |
|  | STRA6 |  |
|  | GBA |  |
|  | CSF3 |  |
|  | MMP8 |  |
|  | FAS |  |
|  | PTEN |  |
|  | CPS1 |  |
|  | PIK3CA |  |
|  | PPARG |  |
|  | COL3A1 |  |
|  | ZFPM2 |  |
|  | XPR1 |  |
|  | SOS1 |  |
|  | GREM1 |  |
|  | CHI3L1 |  |
|  | AGER |  |
|  | ITGAM |  |
|  | SLC34A1 |  |
|  | CXCR2 |  |
|  | RNASE3 |  |
|  | CXCR1 |  |
|  | IL33 |  |
|  | ARG1 |  |
|  | SPINK1 |  |
|  | IGFBP5 |  |
|  | POSTN |  |
|  | NPPA |  |
|  | CD34 |  |
|  | ACTA1 |  |
|  | GSTP1 |  |
|  | GJA5 |  |
|  | LOX |  |
|  | PKD2 |  |
|  | RAF1 |  |
|  | VCAM1 |  |
|  | F3 |  |
|  | CXCL9 |  |
|  | NPHS2 |  |
|  | ADA |  |
|  | GRP |  |
|  | ABCB4 |  |
|  | IL9 |  |
|  | PLAT |  |
|  | FLT4 |  |
|  | CXCL5 |  |
|  | BRAF |  |
|  | MIR199A1 |  |
|  | VWF |  |
|  | CCR4 |  |
|  | VDR |  |
|  | CXCL1 |  |
|  | IL2RB |  |
|  | HP |  |
|  | RET |  |
|  | SERPINH1 |  |
|  | NOD2 |  |
|  | PTPRC |  |
|  | BTNL2 |  |
|  | POLG |  |
|  | KCNMA1 |  |
|  | BMP2 |  |
|  | LPAR1 |  |
|  | TPM1 |  |
|  | SH2B3 |  |
|  | ADCY10 |  |
|  | MYRF |  |
|  | CASP3 |  |
|  | TSC2 |  |
|  | HBB |  |
|  | SMPD1 |  |
|  | SPARC |  |
|  | FLNC |  |
|  | KNG1 |  |
|  | WNT7B |  |
|  | VIP |  |
|  | TBX5 |  |
|  | HLA-A |  |
|  | MIR155 |  |
|  | IGFBP3 |  |
|  | GJA1 |  |
|  | RSPO2 |  |
|  | ATP12A |  |
|  | GSN |  |
|  | HSPA4 |  |
|  | GNRH1 |  |
|  | MYBPC3 |  |
|  | INSL6 |  |
|  | INVS |  |
|  | HRAS |  |
|  | ACD |  |
|  | NRAS |  |
|  | CCR2 |  |
|  | DMD |  |
|  | ADIPOQ |  |
|  | EPO |  |
|  | SLPI |  |
|  | ABCC9 |  |
|  | RAPSN |  |
|  | PRSS1 |  |
|  | NPHP4 |  |
|  | SELP |  |
|  | GJB2 |  |
|  | GFI1 |  |
|  | SERPINA3 |  |
|  | CD80 |  |
|  | ALMS1 |  |
|  | IFT140 |  |
|  | HCN4 |  |
|  | MIR499A |  |
|  | MGP |  |
|  | ENPP1 |  |
|  | NDUFAF6 |  |
|  | ADM |  |
|  | LTA |  |
|  | FHIT |  |
|  | MEN1 |  |
|  | MRC1 |  |
|  | TUBB2B |  |
|  | SOD1 |  |
|  | RBP4 |  |
|  | GPT |  |
|  | TBX20 |  |
|  | GHR |  |
|  | GC |  |
|  | EDNRB |  |
|  | MAP2K1 |  |
|  | TRPC6 |  |
|  | LOXL2 |  |
|  | NOS1 |  |
|  | BGLAP |  |
|  | ACTN2 |  |
|  | TTN-AS1 |  |
|  | ENPP2 |  |
|  | SNAI1 |  |
|  | F13A1 |  |
|  | BLOC1S5 |  |
|  | PLG |  |
|  | KRT7 |  |
|  | DDR1 |  |
|  | CD8A |  |
|  | CCN4 |  |
|  | FBLN5 |  |
|  | AGTR2 |  |
|  | ABCB11 |  |
|  | LAMP2 |  |
|  | SPPL2C |  |
|  | PDE5A |  |
|  | CYP1A1 |  |
|  | SOD3 |  |
|  | HYDIN |  |
|  | THY1 |  |
|  | SETBP1 |  |
|  | IL12A |  |
|  | GDF2 |  |
|  | NPHS1 |  |
|  | NBAS |  |
|  | RBM20 |  |
|  | GATA2 |  |
|  | MEG3 |  |
|  | IFNA1 |  |
|  | DEFB1 |  |
|  | TCAP |  |
|  | CHRNA7 |  |
|  | NAT2 |  |
|  | RBFOX3 |  |
|  | LOC106627981 |  |
|  | S100A1 |  |
|  | PRKN |  |
|  | STAT5B |  |
|  | ACP5 |  |
|  | ADGRV1 |  |
|  | MT-CO3 |  |
|  | ARHGAP31 |  |
|  | KIF7 |  |
|  | MT-CO2 |  |
|  | MTOR |  |
|  | BMPR1B |  |
|  | IFT43 |  |
|  | TRAF3IP1 |  |
|  | MET |  |
|  | TSC1 |  |
|  | SERPINB1 |  |
|  | PROKR2 |  |
|  | DICER1 |  |
|  | VCL |  |
|  | SLC6A4 |  |
|  | CTSG |  |
|  | MIR34A |  |
|  | CD40LG |  |
|  | CD247 |  |
|  | ACTB |  |
|  | PRKCD |  |
|  | THBS1 |  |
|  | MEFV |  |
|  | FIP1L1 |  |
|  | PLN |  |
|  | KRT8 |  |
|  | CCR3 |  |
|  | C3 |  |
|  | SLC12A2 |  |
|  | APOH |  |
|  | HSP90AA1 |  |
|  | PDE4A |  |
|  | LAMA3 |  |
|  | AHI1 |  |
|  | HAMP |  |
|  | NF2 |  |
|  | ATP4A |  |
|  | SGCB |  |
|  | PRKG1 |  |
|  | HSPG2 |  |
|  | HDAC2 |  |
|  | GLA |  |
|  | HFE-AS1 |  |
|  | GSTT1 |  |
|  | GAPDH |  |
|  | NR1H4 |  |
|  | ANGPT1 |  |
|  | GZMB |  |
|  | SIGLEC5 |  |
|  | BAG3 |  |
|  | TGM2 |  |
|  | FKTN |  |
|  | MYPN |  |
|  | KCNQ1 |  |
|  | LCN2 |  |
|  | B2M |  |
|  | CTC1 |  |
|  | NOTCH2 |  |
|  | MUSK |  |
|  | CD40 |  |
|  | CSF1 |  |
|  | ERBB2 |  |
|  | TIMP2 |  |
|  | TF |  |
|  | TAP1 |  |
|  | TTR |  |
|  | MAPK7 |  |
|  | CCND1 |  |
|  | STING1 |  |
|  | PTX3 |  |
|  | ACVR1 |  |
|  | PTPN2 |  |
|  | NPM1 |  |
|  | MED13L |  |
|  | ADSS1 |  |
|  | PRF1 |  |
|  | COL2A1 |  |
|  | FARSB |  |
|  | CR1 |  |
|  | IL3 |  |
|  | SST |  |
|  | PROS1 |  |
|  | LDB3 |  |
|  | NSMF |  |
|  | DNAH8 |  |
|  | GNRHR |  |
|  | IL13RA2 |  |
|  | AFF4 |  |
|  | MIR708 |  |
|  | DNASE1 |  |
|  | ATP8B1 |  |
|  | SLC9A3R1 |  |
|  | PDCD1 |  |
|  | NSD1 |  |
|  | CHAT |  |
|  | HAND2 |  |
|  | MAPT |  |
|  | ACTG2 |  |
|  | LIPA |  |
|  | LTBP1 |  |
|  | HLA-G |  |
|  | PECAM1 |  |
|  | NUP107 |  |
|  | CD28 |  |
|  | MYH11 |  |
|  | NFU1 |  |
|  | SLC17A5 |  |
|  | CSRP3 |  |
|  | MHRT |  |
|  | VPS45 |  |
|  | HLA-DPA1 |  |
|  | CHRM3 |  |
|  | SLC40A1 |  |
|  | CDKN1A |  |
|  | TXN |  |
|  | MYL3 |  |
|  | HTR2A |  |
|  | VCP |  |
|  | BRCA2 |  |
|  | BMPR1A |  |
|  | CDKN3 |  |
|  | PRKAG2 |  |
|  | LRRC56 |  |
|  | ANO1 |  |
|  | GDF15 |  |
|  | LBP |  |
|  | ANGPT2 |  |
|  | NIPBL |  |
|  | NR3C2 |  |
|  | FGA |  |
|  | FOXP1 |  |
|  | IFNA2 |  |
|  | BRCA1 |  |
|  | DKK1 |  |
|  | AQP5 |  |
|  | DUSP6 |  |
|  | ARSB |  |
|  | MYO5A |  |
|  | IFIH1 |  |
|  | MB |  |
|  | FLT1 |  |
|  | LTF |  |
|  | TAC3 |  |
|  | DSG2 |  |
|  | H19 |  |
|  | TEK |  |
|  | CHGA |  |
|  | AXL |  |
|  | TNFRSF1B |  |
|  | MAPK8 |  |
|  | GAA |  |
|  | HS6ST1 |  |
|  | MALAT1 |  |
|  | CD79A |  |
|  | TNFRSF13B |  |
|  | HSPA5 |  |
|  | PRKCA |  |
|  | SDCCAG8 |  |
|  | PF4 |  |
|  | APEX1 |  |
|  | GLI3 |  |
|  | ADRB1 |  |
|  | USB1 |  |
|  | TRIM21 |  |
|  | IRF1 |  |
|  | TLR5 |  |
|  | ATF6 |  |
|  | TNC |  |
|  | MYL2 |  |
|  | TMEM43 |  |
|  | PROK2 |  |
|  | PIK3C2A |  |
|  | CD86 |  |
|  | LTBP2 |  |
|  | ZEB2 |  |
|  | DNAJB1 |  |
|  | SMAD1 |  |
|  | CD14 |  |
|  | RIT1 |  |
|  | UMOD |  |
|  | CHIT1 |  |
|  | TNFSF13B |  |
|  | ASPM |  |
|  | TAFAZZIN |  |
|  | ITGA3 |  |
|  | DYNC2I1 |  |
|  | IL15 |  |
|  | RCBTB1 |  |
|  | H2AC18 |  |
|  | TLL1 |  |
|  | BBIP1 |  |
|  | SGK1 |  |
|  | PITX2 |  |
|  | KITLG |  |
|  | SLC4A4 |  |
|  | CYCS |  |
|  | COPA |  |
|  | TOR1A |  |
|  | GGT1 |  |
|  | ZMPSTE24 |  |
|  | AP3D1 |  |
|  | DAAM2 |  |
|  | BDNF |  |
|  | XBP1 |  |
|  | MUC4 |  |
|  | LACTB |  |
|  | BPI |  |
|  | CD274 |  |
|  | HJV |  |
|  | TGM1 |  |
|  | TLR3 |  |
|  | ADAMTS13 |  |
|  | FOXJ1 |  |
|  | FADD |  |
|  | TLR1 |  |
|  | TNFRSF11A |  |
|  | MECP2 |  |
|  | SLC7A7 |  |
|  | AKAP13 |  |
|  | HLA-C |  |
|  | COL4A3 |  |
|  | IL11 |  |
|  | RAD21 |  |
|  | SLC25A13 |  |
|  | MIR196A2 |  |
|  | LBR |  |
|  | GHSR |  |
|  | FRMD7 |  |
|  | HTR2B |  |
|  | ADGRG6 |  |
|  | C4A |  |
|  | MT-ND1 |  |
|  | PDPN |  |
|  | NTS |  |
|  | WNT3A |  |
|  | ITGB3 |  |
|  | APC |  |
|  | MYLK |  |
|  | MT-ATP6 |  |
|  | CDKN2A |  |
|  | BBS2 |  |
|  | SEMA3E |  |
|  | NCF1 |  |
|  | PLA2G7 |  |
|  | DNAH9 |  |
|  | CAPN3 |  |
|  | TRPV1 |  |
|  | CA4 |  |
|  | COX5A |  |
|  | SDHA |  |
|  | ACTN4 |  |
|  | TPH1 |  |
|  | SNCA |  |
|  | DEFB4A |  |
|  | ALK |  |
|  | SERPINF2 |  |
|  | ABCC6 |  |
|  | HSPA1A |  |
|  | KIF20A |  |
|  | IL6R |  |
|  | ASCL1 |  |
|  | SLC26A4 |  |
|  | SGCD |  |
|  | PPARA |  |
|  | NPPC |  |
|  | PSMB8 |  |
|  | BACH2 |  |
|  | ADORA2B |  |
|  | HMGA2 |  |
|  | IREB2 |  |
|  | CYP1A2 |  |
|  | CRELD1 |  |
|  | DHCR24 |  |
|  | CDC42 |  |
|  | KL |  |
|  | LEPR |  |
|  | CLCN1 |  |
|  | SLC26A3 |  |
|  | COL4A5 |  |
|  | CMA1 |  |
|  | TAP2 |  |
|  | HABP2 |  |
|  | EXOSC3 |  |
|  | F8 |  |
|  | TCIRG1 |  |
|  | PTPRO |  |
|  | ATP5F1A |  |
|  | PIEZO1 |  |
|  | SBDS |  |
|  | IL23R |  |
|  | BBS10 |  |
|  | DNMT1 |  |
|  | FLNB |  |
|  | SRA1 |  |
|  | RPS27A |  |
|  | FTL |  |
|  | DNMT3B |  |
|  | CACNA1C |  |
|  | FOXC1 |  |
|  | PRG2 |  |
|  | DCN |  |
|  | MIR15A |  |
|  | GSR |  |
|  | MUC5B-AS1 |  |
|  | SCARB2 |  |
|  | DNAH11 |  |
|  | PTGIR |  |
|  | MT-ND5 |  |
|  | VEGFC |  |
|  | EDN3 |  |
|  | RPL36A-HNRNPH2 |  |
|  | RRM2B |  |
|  | EZH2 |  |
|  | TAMM41 |  |
|  | TNNC1 |  |
|  | F2R |  |
|  | UNC119 |  |
|  | COMT |  |
|  | LOC107133510 |  |
|  | NEXN |  |
|  | SEMA3A |  |
|  | SOCS3 |  |
|  | PSMC4 |  |
|  | ABCC8 |  |
|  | PGR |  |
|  | XDH |  |
|  | UTS2 |  |
|  | ENTPD1 |  |
|  | CADM1 |  |
|  | PPP1CB |  |
|  | ECE1 |  |
|  | TYMP |  |
|  | MKRN3 |  |
|  | CYBB |  |
|  | CBS |  |
|  | ADAM33 |  |
|  | GATAD1 |  |
|  | CAV3 |  |
|  | IGF1R |  |
|  | FABP3 |  |
|  | PAX2 |  |
|  | FLCN |  |
|  | FGB |  |
|  | SMARCAL1 |  |
|  | CDKN1B |  |
|  | ANXA6 |  |
|  | P2RY2 |  |
|  | ALDH2 |  |
|  | ENO2 |  |
|  | CD163 |  |
|  | SCYL1 |  |
|  | CDH5 |  |
|  | NPC1 |  |
|  | IFRD1 |  |
|  | HTR1B |  |
|  | STAC3 |  |
|  | SLC4A1 |  |
|  | VHL |  |
|  | CBL |  |
|  | CST3 |  |
|  | RASGRP1 |  |
|  | LRRK2 |  |
|  | ATP13A3 |  |
|  | SNAI2 |  |
|  | CYBA |  |
|  | BLOC1S6 |  |
|  | TRB |  |
|  | KCNJ2 |  |
|  | CFH |  |
|  | NAGLU |  |
|  | TPO |  |
|  | ADK |  |
|  | MME |  |
|  | ACADVL |  |
|  | HBG2 |  |
|  | CSF3R |  |
|  | NLRC4 |  |
|  | LRP5 |  |
|  | APOB |  |
|  | IL4R |  |
|  | CCR1 |  |
|  | CRYAB |  |
|  | HSPH1 |  |
|  | PLOD1 |  |
|  | CX3CR1 |  |
|  | DNMT3A |  |
|  | PKP2 |  |
|  | ADORA1 |  |
|  | PAH |  |
|  | TRPM4 |  |
|  | XRCC1 |  |
|  | LPCAT1 |  |
|  | SCN4A |  |
|  | CEP104 |  |
|  | TIMP3 |  |
|  | TYR |  |
|  | STUB1 |  |
|  | AR |  |
|  | ADA2 |  |
|  | CCL20 |  |
|  | PDE4D |  |
|  | MAPKBP1 |  |
|  | GNAQ |  |
|  | NAMPT |  |
|  | GJC1 |  |
|  | TSLP |  |
|  | AQP3 |  |
|  | TGIF1 |  |
|  | RAB27A |  |
|  | PGF |  |
|  | KCNJ11 |  |
|  | PIK3CG |  |
|  | FURIN |  |
|  | TCF4 |  |
|  | BLOC1S3 |  |
|  | COL5A1 |  |
|  | APOL1 |  |
|  | PLA2R1 |  |
|  | FKRP |  |
|  | L1CAM |  |
|  | KDM6A |  |
|  | CREBBP |  |
|  | LPL |  |
|  | ICOS |  |
|  | TP63 |  |
|  | OXT |  |
|  | MMP14 |  |
|  | TNFSF15 |  |
|  | SETD2 |  |
|  | HBA1 |  |
|  | AMBP |  |
|  | ITGAL |  |
|  | MYD88 |  |
|  | CASP1 |  |
|  | ERCC6 |  |
|  | S100A12 |  |
|  | TNFRSF6B |  |
|  | MT-TL1 |  |
|  | DPP4 |  |
|  | HAVCR2 |  |
|  | CACNB4 |  |
|  | CFEOM3C |  |
|  | CFM1 |  |
|  | CHRNA3 |  |
|  | CHRNA5 |  |
|  | COL25A1 |  |
|  | CTEPH1 |  |
|  | DLEC1 |  |
|  | EIF4G1 |  |
|  | EIG1 |  |
|  | EIG2 |  |
|  | EIG3 |  |
|  | EIG4 |  |
|  | EIG5 |  |
|  | EIG7 |  |
|  | FARSA |  |
|  | FMO2 |  |
|  | GABRD |  |
|  | GNAI2 |  |
|  | HCN2 |  |
|  | IGFBP7 |  |
|  | IS1 |  |
|  | IS2 |  |
|  | IS4 |  |
|  | IS5 |  |
|  | JAM2 |  |
|  | KIF21A |  |
|  | LNCR1 |  |
|  | LNCR3 |  |
|  | LNCR4 |  |
|  | LNCR5 |  |
|  | LUCAT1 |  |
|  | MADH9 |  |
|  | MAP3K8 |  |
|  | MARS1 |  |
|  | MYORG |  |
|  | PHOX2A |  |
|  | PLF |  |
|  | PPP2R1B |  |
|  | RB1 |  |
|  | RORB |  |
|  | RPA1 |  |
|  | SCLC1 |  |
|  | SHOXY |  |
|  | SLC12A5 |  |
|  | SLC22A1L |  |
|  | SM2 |  |
|  | SNTG1 |  |
|  | TAPVR1 |  |
|  | TSG11 |  |
|  | ABC3 |  |
|  | ABCC7 |  |
|  | AILJK |  |
|  | AIS |  |
|  | AIS2 |  |
|  | ARIX |  |
|  | BCNG2 |  |
|  | BSCL3 |  |
|  | BWSCR1A |  |
|  | C20orf41 |  |
|  | CAML1 |  |
|  | CD49C |  |
|  | CKN2 |  |
|  | CLAC |  |
|  | CLG |  |
|  | CLOVE |  |
|  | CML33 |  |
|  | COT |  |
|  | CYP2A3 |  |
|  | DAN |  |
|  | DLC1 |  |
|  | DPD1 |  |
|  | EGMA |  |
|  | EIF4G |  |
|  | EIG6 |  |
|  | EJM2 |  |
|  | EJM6 |  |
|  | FARSLB |  |
|  | FEOM4 |  |
|  | FKHL5 |  |
|  | GCFX |  |
|  | GCN2 |  |
|  | GEFSP5 |  |
|  | GLUT1 |  |
|  | GNAI2B |  |
|  | HHC1 |  |
|  | HMOX1D |  |
|  | ICPPS |  |
|  | IGFR2 |  |
|  | KCC2 |  |
|  | KIAA1708 |  |
|  | KRAS2 |  |
|  | LNCR2 |  |
|  | MAC25 |  |
|  | MAR |  |
|  | MARS |  |
|  | MCH5 |  |
|  | MLVAR |  |
|  | NDNL2 |  |
|  | NET37 |  |
|  | NGL |  |
|  | NISBD2 |  |
|  | NS7 |  |
|  | PARK2 |  |
|  | PDGFR |  |
|  | PFBMFT5 |  |
|  | PFBMFT6 |  |
|  | PHN |  |
|  | POIKTMP |  |
|  | PPH1 |  |
|  | PRO1073 |  |
|  | PULAM |  |
|  | RZRB |  |
|  | SCAL1 |  |
|  | SFTB3 |  |
|  | SFTP1 |  |
|  | SFTP2 |  |
|  | SFTP4 |  |
|  | SIS |  |
|  | SLO |  |
|  | SMDP4 |  |
|  | SMDP5 |  |
|  | SPA2 |  |
|  | SSS2 |  |
|  | SYG1 |  |
|  | SYN4 |  |
|  | TASK |  |
|  | TCS1 |  |
|  | TNFSF6 |  |
|  | TRC3 |  |
|  | TUBB4 |  |
|  | VEJAM |  |
|  | ZNF163 |  |
|  | ACDMPV |  |
|  | ALPS2B |  |
|  | APT1LG1 |  |
|  | BRGDA8 |  |
|  | CD32 |  |
|  | CED |  |
|  | CF |  |
|  | CFEOM2 |  |
|  | CFEOM3A |  |
|  | CFEOM5 |  |
|  | CGL3 |  |
|  | COFS1 |  |
|  | COLEC5 |  |
|  | CYP2A |  |
|  | DKCB6 |  |
|  | EA5 |  |
|  | ECA2 |  |
|  | ECA6 |  |
|  | EIG10 |  |
|  | EIG15 |  |
|  | EST |  |
|  | EST2 |  |
|  | FEOM1 |  |
|  | GAPB3 |  |
|  | GEFSP11 |  |
|  | GIP |  |
|  | GLVR2 |  |
|  | HSAS1 |  |
|  | HTLVR |  |
|  | IBGC4 |  |
|  | IBGC5 |  |
|  | IBGC6 |  |
|  | IBGC8 |  |
|  | ILD1 |  |
|  | IMPT1 |  |
|  | KIAA1161 |  |
|  | KIAA1176 |  |
|  | KIAA1338 |  |
|  | MADH6 |  |
|  | MAGEG1 |  |
|  | MCAP |  |
|  | MTRNS |  |
|  | NEU |  |
|  | NHL |  |
|  | PAOD2 |  |
|  | PAPPAS |  |
|  | PARK18 |  |
|  | PCAR1 |  |
|  | PDJ |  |
|  | PNKD3 |  |
|  | POVD1 |  |
|  | PPH4 |  |
|  | RAMSVPS |  |
|  | RASK2 |  |
|  | RILDBC1 |  |
|  | RILDBC2 |  |
|  | SCN2 |  |
|  | SMDP1 |  |
|  | SMDP2 |  |
|  | SMDP3 |  |
|  | SS |  |
|  | TR |  |
|  | BAIPRCK |  |
|  | CADEDS |  |
|  | CDCBM1 |  |
|  | CFEOM1 |  |
|  | CSB |  |
|  | DEE34 |  |
|  | DKCA1 |  |
|  | DKCA2 |  |
|  | DYT18 |  |
|  | EGI11 |  |
|  | EIG18 |  |
|  | EIG9 |  |
|  | EJM7 |  |
|  | 44594 |  |
|  | FIH |  |
|  | HALD4 |  |
|  | HER2 |  |
|  | IBDIMDE |  |
|  | IBGC1 |  |
|  | IBGC7 |  |
|  | ILD2 |  |
|  | IMF1 |  |
|  | JEB7 |  |
|  | KIAA1088 |  |
|  | LICS |  |
|  | MASA |  |
|  | MCM |  |
|  | METRS |  |
|  | MRP7 |  |
|  | NS |  |
|  | P450C2A |  |
|  | PFBMFT4 |  |
|  | PHOG |  |
|  | PPH2 |  |
|  | PPH3 |  |
|  | PVOD2 |  |
|  | TPL2 |  |
|  | ALPS1B |  |
|  | ARMD5 |  |
|  | CFC2 |  |
|  | CFEOM3B |  |
|  | DKCB4 |  |
|  | DKCB5 |  |
|  | EIG14 |  |
|  | EIG17 |  |
|  | EIG8 |  |
|  | EJM8 |  |
|  | IBGC2 |  |
|  | IEG16 |  |
|  | ILLD |  |
|  | LCCNS |  |
|  | MCMTC |  |
|  | PED |  |
|  | PENTT |  |
|  | PFBMFT2 |  |
|  | SPG1 |  |
|  | VSCN2 |  |
|  | CMT2U |  |
|  | CWS5 |  |
|  | DKCA4 |  |
|  | GLUT1DS |  |
|  | HYPOC1 |  |
|  | KOGS |  |
|  | LIWAS |  |
|  | LKPAT |  |
|  | PFBMFT1 |  |
|  | RALD |  |
|  | UVSS1 |  |
|  | CLAPO |  |
|  | CMM9 |  |
|  | EIG12 |  |
|  | HALD2 |  |
|  | OES |  |
|  | PFBMFT3 |  |
|  | POF11 |  |
|  | TTD9 |  |
|  | CCM4 |  |
|  | DYT9 |  |
|  | SDCHCN |  |
|  | D2R |  |
|  | GPR40 |  |
|  | CTGF |  |
|  | PFKFB3 |  |
|  | NPTX2 |  |
|  | ITGAV |  |
|  | ITGB6 |  |
|  | JNK |  |
|  | PDHK1 |  |
|  | GPR84 |  |
|  | PDE3 |  |
|  | VEGFR |  |

| ***Supplementary Material***  **Table S5 Information Regarding the Crucial Gene Cluster** | | | |
| --- | --- | --- | --- |
| **Gene** | **BetweennessCentrality** | **ClosenessCentrality** | **Degree** |
| AKT1 | 0.09446316 | 0.66666667 | 74 |
| HSP90AA1 | 0.06300924 | 0.62857143 | 61 |
| SRC | 0.05991731 | 0.65024631 | 67 |
| PTGS2 | 0.05843314 | 0.61111111 | 58 |
| EGFR | 0.05442597 | 0.63461538 | 65 |
| NOS3 | 0.05202916 | 0.56896552 | 41 |
| ESR1 | 0.04393368 | 0.62264151 | 58 |
| MMP9 | 0.0429378 | 0.59192825 | 52 |
| MAPK3 | 0.03590675 | 0.61971831 | 61 |
| PPARA | 0.02662108 | 0.54771784 | 36 |
| MTOR | 0.0239604 | 0.60273973 | 55 |
| JAK2 | 0.02241288 | 0.54545455 | 36 |
| HIF1A | 0.02115287 | 0.59459459 | 53 |
| CYP3A4 | 0.01929129 | 0.53441296 | 29 |
| NR3C1 | 0.01912151 | 0.53658537 | 31 |
| REN | 0.0182888 | 0.53658537 | 30 |
| MAPK14 | 0.01748064 | 0.54320988 | 34 |
| F2 | 0.01608235 | 0.52380952 | 24 |
| SIRT1 | 0.01592359 | 0.55932203 | 38 |
| ADRB2 | 0.01577115 | 0.51968504 | 19 |
| MAPK1 | 0.01521367 | 0.57641921 | 47 |
| MAPT | 0.01516357 | 0.4962406 | 22 |
| CCND1 | 0.01491121 | 0.57391304 | 47 |
| ABCB1 | 0.0128448 | 0.52380952 | 25 |
| MMP1 | 0.01214002 | 0.49438202 | 19 |
| MMP2 | 0.01167072 | 0.55 | 36 |
| MPO | 0.0110804 | 0.515625 | 25 |
| PGR | 0.01058713 | 0.53225806 | 31 |
| CYP2C9 | 0.01027706 | 0.4962406 | 20 |
| TERT | 0.00857152 | 0.4962406 | 25 |
| ITGB3 | 0.00753941 | 0.52173913 | 22 |
| GSK3B | 0.00749699 | 0.54098361 | 32 |
| KIT | 0.00722214 | 0.53658537 | 34 |
| KDR | 0.00631376 | 0.53877551 | 33 |
| PIK3CA | 0.00587913 | 0.55 | 42 |
| MDM2 | 0.00439459 | 0.53441296 | 35 |
| PTGS1 | 0.00434455 | 0.4765343 | 15 |
| MMP3 | 0.00399369 | 0.4962406 | 19 |
| MAPK8 | 0.00393228 | 0.54545455 | 33 |
| CREBBP | 0.00358862 | 0.51162791 | 23 |
| PRKCA | 0.00357878 | 0.52173913 | 25 |
| NOS1 | 0.00355824 | 0.48888889 | 15 |
| LRRK2 | 0.00316069 | 0.48 | 15 |
| JAK3 | 0.00311915 | 0.4765343 | 18 |
| CYP19A1 | 0.00298052 | 0.49070632 | 19 |
| AR | 0.00260173 | 0.52589641 | 31 |
| AKT1 | 0.09446316 | 0.66666667 | 74 |
| HSP90AA1 | 0.06300924 | 0.62857143 | 61 |
| SRC | 0.05991731 | 0.65024631 | 67 |
| PTGS2 | 0.05843314 | 0.61111111 | 58 |
| EGFR | 0.05442597 | 0.63461538 | 65 |
| NOS3 | 0.05202916 | 0.56896552 | 41 |
| ESR1 | 0.04393368 | 0.62264151 | 58 |
| MMP9 | 0.0429378 | 0.59192825 | 52 |
| MAPK3 | 0.03590675 | 0.61971831 | 61 |
| PPARA | 0.02662108 | 0.54771784 | 36 |
| MTOR | 0.0239604 | 0.60273973 | 55 |
| JAK2 | 0.02241288 | 0.54545455 | 36 |
| HIF1A | 0.02115287 | 0.59459459 | 53 |
| CYP3A4 | 0.01929129 | 0.53441296 | 29 |
| NR3C1 | 0.01912151 | 0.53658537 | 31 |
| REN | 0.0182888 | 0.53658537 | 30 |
| MAPK14 | 0.01748064 | 0.54320988 | 34 |
| F2 | 0.01608235 | 0.52380952 | 24 |
| SIRT1 | 0.01592359 | 0.55932203 | 38 |
| ADRB2 | 0.01577115 | 0.51968504 | 19 |
| MAPK1 | 0.01521367 | 0.57641921 | 47 |
| MAPT | 0.01516357 | 0.4962406 | 22 |
| CCND1 | 0.01491121 | 0.57391304 | 47 |
| ABCB1 | 0.0128448 | 0.52380952 | 25 |
| MMP1 | 0.01214002 | 0.49438202 | 19 |
| MMP2 | 0.01167072 | 0.55 | 36 |
| MPO | 0.0110804 | 0.515625 | 25 |
| PGR | 0.01058713 | 0.53225806 | 31 |
| CYP2C9 | 0.01027706 | 0.4962406 | 20 |
| TERT | 0.00857152 | 0.4962406 | 25 |
| ITGB3 | 0.00753941 | 0.52173913 | 22 |
| GSK3B | 0.00749699 | 0.54098361 | 32 |
| KIT | 0.00722214 | 0.53658537 | 34 |
| KDR | 0.00631376 | 0.53877551 | 33 |
| PIK3CA | 0.00587913 | 0.55 | 42 |
| MDM2 | 0.00439459 | 0.53441296 | 35 |
| PTGS1 | 0.00434455 | 0.4765343 | 15 |
| MMP3 | 0.00399369 | 0.4962406 | 19 |
| MAPK8 | 0.00393228 | 0.54545455 | 33 |
| CREBBP | 0.00358862 | 0.51162791 | 23 |
| PRKCA | 0.00357878 | 0.52173913 | 25 |
| NOS1 | 0.00355824 | 0.48888889 | 15 |
| LRRK2 | 0.00316069 | 0.48 | 15 |
| JAK3 | 0.00311915 | 0.4765343 | 18 |
| CYP19A1 | 0.00298052 | 0.49070632 | 19 |
| AR | 0.00260173 | 0.52589641 | 31 |
